# Examining variations in prescribing safety in UK general practice: cross sectional study using the Clinical Practice Research Datalink

**DOI:** 10.1136/bmj.h5501

**Published:** 2015-11-03

**Authors:** S Jill Stocks, Evangelos Kontopantelis, Artur Akbarov, Sarah Rodgers, Anthony J Avery, Darren M Ashcroft

**Affiliations:** 1 NIHR Greater Manchester Primary Care Patient Safety Translational Research Centre, Centre for Primary Care, Institute of Population Health, University of Manchester, Manchester M13 9PL, UK; 2 NIHR School for Primary Care Research, Centre for Primary Care, Institute of Population Health, University of Manchester, Manchester, UK; 3 Centre for Health Informatics, Institute of Population Health, University of Manchester, Manchester, UK; 4 Division of Primary Care, University of Nottingham Medical School, Queen’s Medical Centre, Nottingham, UK; 5 Centre for Pharmacoepidemiology and Drug Safety, Manchester Pharmacy School, University of Manchester, Manchester Academic Health Sciences Centre (MAHSC), Manchester, UK

## Abstract

**Study question** What is the prevalence of different types of potentially hazardous prescribing in general practice in the United Kingdom, and what is the variation between practices?

**Methods** A cross sectional study included all adult patients potentially at risk of a prescribing or monitoring error defined by a combination of diagnoses and prescriptions in 526 general practices contributing to the Clinical Practice Research Datalink (CPRD) up to 1 April 2013. Primary outcomes were the prevalence of potentially hazardous prescriptions of anticoagulants, anti-platelets, NSAIDs, β blockers, glitazones, metformin, digoxin, antipsychotics, combined hormonal contraceptives, and oestrogens and monitoring by blood test less frequently than recommended for patients with repeated prescriptions of angiotensin converting enzyme inhibitors and loop diuretics, amiodarone, methotrexate, lithium, or warfarin.

**Study answer and limitations** 49 927 of 949 552 patients at risk triggered at least one prescribing indicator (5.26%, 95% confidence interval 5.21% to 5.30%) and 21 501 of 182 721 (11.8%, 11.6% to 11.9%) triggered at least one monitoring indicator. The prevalence of different types of potentially hazardous prescribing ranged from almost zero to 10.2%, and for inadequate monitoring ranged from 10.4% to 41.9%. Older patients and those prescribed multiple repeat medications had significantly higher risks of triggering a prescribing indicator whereas younger patients with fewer repeat prescriptions had significantly higher risk of triggering a monitoring indicator. There was high variation between practices for some indicators. Though prescribing safety indicators describe prescribing patterns that can increase the risk of harm to the patient and should generally be avoided, there will always be exceptions where the indicator is clinically justified. Furthermore there is the possibility that some information is not captured by CPRD for some practices—for example, INR results in patients receiving warfarin.

**What this study adds** The high prevalence for certain indicators emphasises existing prescribing risks and the need for their appropriate consideration within primary care, particularly for older patients and those taking multiple medications. The high variation between practices indicates potential for improvement through targeted practice level intervention.

**Funding, competing interests, data sharing **National Institute for Health Research through the Greater Manchester Primary Care Patient Safety Translational Research Centre (grant No GMPSTRC-2012-1). Data from CPRD cannot be shared because of licensing restrictions.

## Introduction

Prescribing errors in primary care can cause considerable harm, with adverse drug events accounting for around 7% of hospital admissions in the United Kingdom, and half of these are judged to be preventable.^1^ ^2^ ^3^ In 2012 the Practice Study found that one in 20 prescription items was associated with a clinically important error and one in 550 was associated with a serious error.^4^ Prescribing safety indicators (indicators) define prescribing patterns that can increase the risk of harm to the patient and should generally be avoided, though there will always be exceptions where the indicator is justified for clinical reasons.

Several sets of indicators have been developed for use in primary care in the UK.^5^ ^6^ ^7^ ^8^ ^9^ ^10^ The widespread use of electronic prescribing systems and primary care patient records facilitates the analysis of aggregate patient records to estimate the prevalence of indicators and their variation in prevalence between practices and to examine the patient and practice factors that influence their occurrence. In Scotland, a set of indicators was developed through consensus between GPs and pharmacists and their prevalence was measured in 315 general practices.^9^ In England, a pharmacist led intervention was found to reduce the frequency of indicators in general practice (PINCER trial),^10^ while in Scotland, validated measures of high risk prescribing were the outcome measures in a trial of an intervention to reduce high risk prescribing of non-steroidal anti-inflammatory drugs (NSAIDs) and antiplatelet agents in general practice.^11^ The set of indicators used for the PINCER trial was developed through consensus among GPs^8^ and has been expanded and made available for GPs to use as an audit tool since 2013.^8^ ^10^ ^12^ ^13^ Currently GPs in the UK are encouraged to report clinical audit or quality improvement projects as part of their revalidation portfolio.^14^

If these indicators are to be used by GPs as an audit tool there needs to be an agreement about what constitutes a clinically acceptable prevalence of high risk prescribing and whether there are potential exceptions. Furthermore, the ability of indicators to discriminate between practices and the relation between their prevalence and patient and/or practice characteristics needs to be understood; identifying groups of patients at increased risk of indicators will enable a targeted approach to reducing their occurrence. As far as we know, indicators have not been investigated in a large sample of UK-wide general practices.

We used anonymised patient level data from the Clinical Practice Research Datalink (CPRD)^15^ to investigate the prevalence and predictors of previously described indicators^8^ ^10^ ^12^ ^13^ in UK general practice. We measured the prevalence of indicators identifying patients at increased risk of a prescribing error during a time period leading up to 1 April 2013; examined the variation in the prevalence of the above indicators between practices using intraclass correlation coefficients and the meaning for the individual practice using reliability estimates; and examined the associations between the indicators and patient characteristics (age, sex, number of repeat medications) and practice characteristics (list size, practice level deprivation, geographical location).

## Methods

We included in the analysis all patients aged 18 or over who were registered with one of the CPRD practices that had uploaded data of research quality on or after 1 April 2013. Each indicator consisted of a denominator and a numerator (table 1). The denominator included all patients with the potential to trigger an indicator because of an existing diagnosis or prescribing pattern (during a time period specific to the indicator definition and the audit date, table 1). The numerator consisted of those patients who actually triggered the indicator by receiving the potentially unsafe prescription or having no record of the required monitoring during the time period leading up to the audit date (table 1. Therefore the prevalence of an indicator describes the proportion of patients with the potential to trigger an indicator who actually did so. (A full list of codes to define each indicator has been uploaded to www.clinicalcodes.org.^16^) For the main analysis the prevalence of each indicator was measured relative to 1 April 2013 (the audit date), and the study is cross sectional at this date. In a further analysis, however, we used a rolling time window of audit dates representing the 1st of the month from May to September 2013 to check whether the results were sensitive to the choice of audit date. For long term conditions, or a diagnosis associated with contraindicated prescribing, the cumulative number of patients since their first recorded diagnosis formed the denominator. For asthma diagnoses, patients were excluded from the denominator after an asthma resolved code but would return to the denominator if this were superseded by a new asthma diagnosis code (P1, table 1, fig 1). When the condition or diagnosis was reversible, such as requiring monitoring with repeat prescriptions, the denominator was the number of patients prescribed a repeat medication within a fixed time window before the audit date. For example, the denominator for repeat prescribing of angiotensin converting enzyme inhibitor (ACEI) or loop diuretic (M1, table 1, fig 1) includes patients with at least one prescription of (ACEI) or loop diuretic between 6 and 15 months before the audit date and a repeat prescription between 0 and 6 months before the audit date. Polypharmacy was defined as the number of medications with at least two prescriptions within the 12 months leading up to 1 April 2013.

**Table 1 tbl1:** Definition of each prescribing safety indicator in general practice

ID	Patients at risk of prescribing safety indicator (denominator)	Patients receiving prescribing safety indicator (numerator)
P1	Has asthma Read code at least 6 months before audit date and no asthma resolved code since latest asthma code	Had β blocker prescription within 6 months leading up to audit date
P2	Has asthma Read code at least 3 months before audit date and prescribed LABA inhaler within 3 months leading up to audit date	Not prescribed ICS or combined LABA-ICS within 3 months leading up to audit date
P3	Has Read code for peptic ulcer at least 6 months before audit date and not prescribed PPI, misoprostol, or H2 antagonist within 6 months leading up to audit date	Prescribed aspirin or clopidogrel within 6 months leading up to audit date
P4	Has Read code for peptic ulcer at least 6 months before audit date and not prescribed PPI, misoprostol, or H2 antagonist within 6 months leading up to audit date	Prescribed non-selective NSAID within 6 months leading up to audit date
P5	Has Read code for heart failure at least 3 months before audit date	Prescribed glitazone within 3 months leading up to audit date
P6	Has Read code for heart failure at least 3 months before audit date	Prescribed NSAID within 3 months leading up to audit date
P7	Has Read code for chronic kidney disease stage 3B, 4, or 5 at least 3 months before audit date	Prescribed NSAID within 3 months leading up to audit date
P8	Has Read code for chronic kidney disease stage 4 or 5 at least 3 months before audit date	Prescribed metformin within 3 months leading up to audit date
P9	Has Read code for chronic kidney disease stage 3B, 4 or 5 at least 3 months before audit date	Prescribed digoxin at daily dose >125 µg within 3 months leading up to audit date
P10	Has a prescription for warfarin and not prescribed PPI, misoprostol or H2 antagonist within 6 months leading up to audit date	Prescribed NSAID within 6 months leading up to audit date
P11	Has a prescription for warfarin and not prescribed PPI, misoprostol or H2 antagonist within 6 months leading up to audit date	Prescribed aspirin within 6 months leading up to audit date
P12	Aged ≥65 on audit date and has Read code for dementia and no Read code for psychosis at least 6 months before audit date	Prescribed antipsychotics at least twice and 3 months apart within 6 months leading up to audit date
P13	Aged ≥65 on audit date and not prescribed PPI, misoprostol or H2 antagonist within 6 months leading up to audit date	Prescribed NSAID within 6 months leading up to audit date
P14	Has Read code for chronic kidney disease stage 3B, 4, or 5 and prescribed ACEI	Prescribed NSAID within 3 months leading up to audit date
P15	Has Read code for chronic kidney disease stage 3B, 4, or 5 and prescribed ACEI and loop diuretic	Prescribed NSAID within 3 months leading up to audit date
P16	Female and has Read code for venous or arterial thrombosis at least 6 months before audit date	Prescribed CHC within 6 months leading up to audit date
P17	Female and Read code for BMI >40 between 66 and 6 months before audit date and without more recent Read code for BMI <40	Prescribed CHC within 6 months leading up to audit date
P18	Female, aged >35, and most recent Read code for smoking status is current smoker at least 6 months before audit date	Prescribed CHC within 6 months leading up to audit date
P19	Female and has Read code for breast cancer at least 6 months before audit date	Prescribed oral or transdermal oestrogens within 6 months leading up to audit date
M1	Aged ≥75 and prescribed ACEI or loop diuretic 15-6 months before audit date and again within 6 months leading up to audit date	Have NOT had renal function or electrolytes test in 15 months leading up to audit date
M2	Prescribed amiodarone 6-12 months before audit date and again within 6 months leading up to audit date	Have NOT had thyroid function test in 6 months leading up to audit date
M3	Prescribed methotrexate 3-6 months before audit date and again within 3 months leading up to audit date	Have NOT had liver function test or full blood count in 3 months leading up to audit date
M4	Prescribed lithium 3-6 months before audit date and again within 3 months leading up to audit date	Have NOT had lithium level test in 3 months leading up to audit date
M5	Prescribed warfarin 3-6 months before audit date and again within 3 months leading up to audit date	Have NOT had INR check in 3 months leading up to audit date

ACEI=angiotensin converting enzyme inhibitor; BMI=body mass index; CHC=combined hormone contraceptive; ICS=inhaled corticosteroid; LABA=long acting β2 agonist; NSAID=non-steroidal anti-inflammatory drug (non-selective); PPI=proton pump inhibitor; INR=international normalised ratio.

**Figure fig1:**
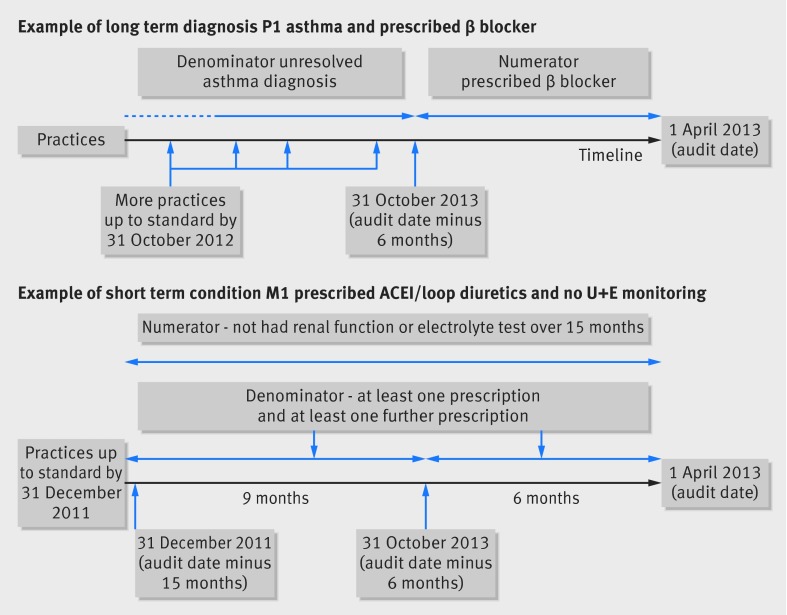
**Fig 1** Examples of indicator definitions

### Statistical methods

We calculated the proportion of patients triggering each indicator (with 95% confidence intervals from a binomial distribution) for each indicator and for a composite prescribing (prescribing indicator, P1-P13) and monitoring (monitoring indicator, M1-M4) indicator. A further composite indicator also included the indicators relevant only to women in (P1-P13 plus P16-P19). Each patient counted just once in the composite indicator with a triggered indicator taking priority if a patient entered the denominator for more than one indicator. Therefore the composite indicator describes the number of patients triggering at least one indicator divided by all patients with the potential to trigger an indicator. The variation in prevalence between practices was quantified for each indicator by an empty mixed effects two level logistic regression model with patients nested within practices (using the xtmelogit command in Stata). The outcome was a binary variable (1 or 0) with patients triggering an indicator designated “1” and those with potential to trigger an indicator but not doing so designated “0.” We used the post-estimation estat command in Stata to derive the intraclass correlation coefficients (ICCs) for each indicator. The intraclass correlation coefficient quantifies the proportion of the total variation in an indicator that is accounted for by the variation between practices (τ^2^)—that is, indicators with low values of intraclass correlation coefficient show that there is more variability within practices (σ^2^) rather than between them (τ^2^), in relation to the outcome (fig 2).[Fig fig2]

**Figure fig2:**
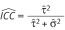
**Fig 2** Formula for proportion of total variation in indicator that is accounted for by variation between practices

The within-variance in this binary outcome context is non-intuitive and fixed in the logistic regression models, and it does not reflect variability in the performance of health professionals within a practice. Ideally, rather than being empty, the model would be adjusted by patient level variables reflecting the case mix before estimation of the intraclass correlation coefficient. As the adjusted model did not converge for some of the indicators, the empty model was the best option for consistency across all indicators. Adjustment for age, sex, and polypharmacy did not alter the intraclass correlation coefficient for most of the indicators where the model did converge. The intraclass correlation coefficient does not intuitively translate into an understanding of the implications for the individual practice. Therefore, to help with interpretation of the ICC, we calculated the reliability for different numbers of patients in the denominator using the Spearman-Brown prophecy formula as described previously (fig 3).^9^

**Figure fig3:**
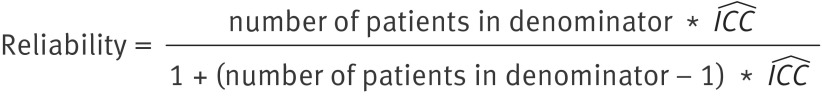
**Fig 3** Spearman-Brown prophecy formula

For many situations a reliability of 0.7 is considered acceptable, but higher values might be preferable in comparisons of prescribing safety between practices.^9^ We calculated the reliability for a hypothetical practice with the median number of patients in the denominator, and appendix 1 shows the number of practices with reliabilities greater than 0.7, 0.8, and 0.9. However, the intraclass correlation coefficient, and therefore reliability, cannot provide insight on the underlying causes of the variation between practices that might, or might not, be clinical in nature. To further describe the heterogeneity between practices we also reported the prevalence predicted by the regression model for each indicator with 95% prediction intervals as described elsewhere.^18^ The prediction intervals describe the expected range of prevalence for a new practice (with 95% confidence).

The composite indicators were further analysed with the same mixed effects two level logistic regression model. As described above each patient counted just once in this analysis with the outcome variable “1” designating patients triggering at least one indicator and “0” designating patients with the potential to trigger at least one indicator but not doing so. We analysed indicators related to prescribing (prescribing indicators) separately from indicators related to monitoring (monitoring indicators). The practice level predictors were list size, intervals of the index of multiple deprivation (IMD) based on 2010 estimates,^17^ and location of practice by country or region of the UK. Patient level predictors were age, sex, and polypharmacy.

### Patient involvement

Given our specific aims, no patients were involved in setting the research question or the outcome measures, nor were they involved in the design and implementation of the study. We will be working with the Research User Group at the NIHR Greater Manchester Primary Care Patient Safety Translational Research Centre to advise on plans for dissemination of these findings.

## Results

Data were available from 526 practices and almost five million patients. The mean list size between 1 April 2012 and 31 March 2013 was 9410 (standard deviation 4971). Most practices were in England (384, 73%), followed by Scotland (72, 14%), Wales (49, 9%), and Northern Ireland (21, 4%). More details on the make-up of the CPRD practices and their representativeness of the UK primary care have been provided elsewhere.^19^

The choice of audit date did not greatly affect the prevalence of the indicator (comparing audit date 1 April 2013 with a rolling time window 1 May-1 September 2013) except for M4 (prescription of lithium without a lithium level test). The lower prevalence when we used the 1 April audit date was because of an increased frequency of lithium level tests during January 2013. Analysis that used earlier audit dates showed that the January increase in tests occurred every year as far back as 2005 (data available from authors).

Tables 2 and 3 and figure 4 summarise the observed prevalence, predicted prevalence, and ICC for each indicator and the composite indicators. The distribution of the observed prevalence for each indicator by practices and by list size is summarised in appendix 1. The prevalence of the monitoring indicators was consistently higher than prescribing indicators (fig 4). The prescribing indicators with a higher prevalence were mainly those related to the prescribing of NSAIDs and aspirin without gastroprotection (P3-P4, P11, P13, fig 4), whereas the prescribing of combined hormone contraceptives (CHC) and oestrogens was associated with a lower prevalence (P16-P19, fig 4).

**Table 2 tbl2:** Summary of prevalence and variation between practices for each prescribing safety indicator

Indicator and (short) description	Numerator/ denominator (1 April 2013)	Observed prevalence (95% CI)	ICC* (95% CI)	Rel†	Predicted prevalence (95% prediction intervals)‡	Prevalence (95% CI) 1 May-1 Sept
P1: asthma diagnosis (unresolved) and prescribed β blockers	11 599/308 624	3.76% (3.69 to 3.83)	0.03 (0.02 to 0.04)	0.94	3.6% (1.9 to 6.6)	3.81% (3.78 to 3.84)
P2: asthma diagnosis and prescribed a LABA inhaler without ICS	1076/75 089	1.43% (1.35 to 1.52)	0.18 (0.14 to 0.22)	0.88	1.1% (0.2 to 5.4)	1.39% (1.36 to 1.42)
P3: history of peptic ulceration or GI bleed, not prescribed gastro-protection and prescribed aspirin or clopidogrel	1470/14 392	10.21% (9.73 to 10.72)	0.04 (0.02 to 0.06)	0.44	9.5% (5.0 to 17.3)	10.23% (10.03 to 10.43)
P4: history of peptic ulceration or GI bleed, not prescribed gastro-protection and prescribed NSAID	592/14 392	4.11% (3.80 to 4.45)	0.06 (0.03 to 0.10)	0.57	3.7% (1.6 to 8.5)	4.13% (4.00 to 4.27)
P5: history of heart failure and prescribed glitazone	113/30 171	0.37% (0.31 to 0.45)	0.16 (0.07 to 0.33)	0.91	0.3% (0.1 to 0.4)	0.35% (0.32 to 0.38)
P6: history of heart failure and prescribed NSAID	854/30 171	2.83% (2.65 to 3.02)	0.05 (0.03 to 0.08)	0.72	2.6% (1.2 to 5.7)	2.87% (2.80 to 2.95)
P7: diagnosis of CKD stage 3B, 4 or 5 and prescribed NSAID	776/22 640	3.43% (3.20 to 3.67)	0.08 (0.05 to 0.12)	0.70	2.7% (0.9 to 7.4)	3.49% (3.39 to 3.59)
P8: diagnosis of CKD stage 4 or 5 and prescribed metformin	1567/20 185	7.76% (7.40 to 8.14)	0.17 (0.13 to 0.21)	0.84	4.3% (0.9 to 18.7)	7.78% (7.63 to 7.93)
P9: diagnosis of CKD stage 3B, 4 or 5 and prescribed digoxin, daily dose >125 µg	209/22 640	0.92% (0.81 to 1.06)	0.13 (0.07 to 0.22)	0.79	0.7% (0.2 to 2.9)	0.88% (0.83 to 0.93)
P10: prescribed warfarin, not prescribed gastro-protection and prescribed NSAID	581/40 441	1.44% (1.33 to 1.56)	0.10 (0.07 to 0.15)	0.89	1.2% (0.4 to 4.0)	1.37% (1.33 to 1.42)
P11: prescribed warfarin, not prescribed gastro-protection and prescribed aspirin	3072/40 441	7.60% (7.34 to 7.86)	0.03 (0.02 to 0.04)	0.66	7.4% (4.2 to 12.6)	7.46% (7.35 to 7.56)
P12: diagnosis of dementia, age >65, no psychosis diagnosis and prescribed antipsychotics	2244/27 763	8.08% (7.77 to 8.41)	0.10 (0.08 to 0.12)	0.82	7.0% (2.3 to 19.5)	8.08% (7.95 to 8.22)
P13: aged ≥65, not prescribed gastro- protection and prescribed NSAID	27 381/611 603	4.48% (4.43 to 4.53)	0.09 (0.07 to 0.10)	0.99	4.1% (1.4 to 11.5)	4.46% (4.44 to 4.48)
Summary for P1-P13:						
P1-P13: at least one prescribing indicator	49 927/949 552	5.26% (5.21 to 5.30)	0.04 (0.03 to 0.05)	0.99	5.1% (2.5 to 10.3)	5.25% (5.24 to 5.28)
P1-P12: as above and excluding P13	23 500/430 005	5.47% (5.40–5.53)	0.025 (0.02 to 0.03)	0.95	5.3% (3.1 to 9.1)	5.48% (5.45 to 5.55)
P14: diagnosis of CKD stage 3B, 4 or 5 and prescribed ACEI (+ comb loop) and NSAID	443/12 128	3.65% (3.33 to 4.00)	0.07 (0.04 to 0.12)	0.49	3.1% (1.2 to 7.9)	3.64% (3.51 to 3.77)
P15: diagnosis of CKD stage 3B, 4 or 5 and ACEI and loop diuretic and NSAID	123/4351	2.83% (2.37 to 3.36)	0.04 (0.01 to 0.25)	0.19	2.6% (1.2 to 5.6)	2.94% (2.75 to 3.14)
P16: history of venous or arterial thrombosis and prescribed CHC	204/72 666	0.28% (0.24 to 0.32)	0.20 (0.13 to 0.29)	0.97	0.2% (0.0 to 1.2)	0.28% (0.26 to 0.29)
P17: BMI ≥40 and prescribed CHC	546/54 138	1.00% (0.93 to 1.10)	0.15 (0.11 to 0.21)	0.47	0.8% (0.2 to 3.5)	0.96% (0.93 to 1.00)
P18: current smoker and prescribed CHC	1674/213 673	0.78% (0.75 to 0.82)	0.09 (0.07 to 0.11)	0.97	0.7% (0.2 to 2.1)	0.71% (0.70 to 0.72)
P19: history of breast cancer and prescribed oestrogens	372/34 238	1.09% (0.98 to 1.20)	0.11 (0.07 to 0.18)	0.88	0.9% (0.2 to 3.0)	1.12% (1.07 to 1.16)
Summary for P16-P19:						
P16-P19: At least one female prescribing indicator	2771/349 552	0.79% (0.76 to 0.82)	0.07 (0.06 to 0.09)	0.98	0.7% (0.3 to 2.0)	0.74% (0.73 to 0.76)
P1-P13 and P16-P19: at least one prescribing indicator including female indicators	52 671/1 195 408	4.41% (4.37 to 4.44)	0.04 (0.03 to 0.04)	0.99	4.3% (2.2 to 8.2)	4.40% (4.38 to 4.41)

ACEI=angiotensin converting enzyme inhibitor; BMI=body mass index; CHC=combined hormone contraceptive; CKD=chronic kidney disease; FBC=full blood count; GI=gastrointestinal; ICS=inhaled corticosteroid; INR=international normalised ratio; LABA=long acting β2 agonist; NSAID=non-steroidal anti-inflammatory drug (non-selective); TFT=thyroid function test.

*Intraclass correlation coefficient.

†Reliability for practice with median number of patients at risk of triggering indicator.

‡Predicted prevalence in new practice with 95% prediction intervals using logistic regression model;

**Table 3 tbl3:** Summary of prevalence and variation between practices for each monitoring safety indicator

Indicator and (short) description	Numerator/ denominator (1 April 2013)	Observed prevalence (95% CI)	ICC* (95% CI)	Rel†	Predicted prevalence (95% prediction intervals)‡	Prevalence (95% CI) 1 May-1 Sept
M1: aged 75 and ACEI or loop diuretic without U+E monitoring	17 092/164 630	10.4% (10.2 to 10.5)	0.21 (0.19 to 0.24)	0.99	8.3% (1.4 to 36.8)	10.9% (10.9 to 11.0)
M2: repeat amiodarone without TFT	1596/3809	41.9% (40.3 to 43.5)	0.13 (0.10 to 0.17)	0.47	41.5% (15.2 to 73.7)	42.2% (41.6 to 42.9)
M3: repeat methotrexate without LFT or FBC	2117/13 231	16.0% (15.4 to 16.6)	0.41 (0.36 to 0.45)	0.94	11.1% (0.6 to 70.5)	16.7% (16.4 to 17.0)
M4: repeat lithium without lithium concentration check	798/4126	19.3% (18.2 to 20.6)	0.12 (0.08 to 0.17)	0.49	17.5% (5.4 to 44.3)	33.9% (33.3 to 34.5)
M5: repeat warfarin without INR	16 463/51 008	32.3% (31.9 to 32.7)	0.78 (0.75 to 0.80)	1.00	19.6% (0.0 to 99.5)	32.4% (32.2 to 32.6)
M1-M4: at least one monitoring indicator	21 501/182 721	11.8% (11.6 to 11.9)	0.17 (0.15 to 0.19)	0.98	10.2% (2.2 to 36.0)	11.7% (11.6 to 11.7)

ACEI=angiotensin converting enzyme inhibitor; U+E=urea and electrolyte monitoring; FBC=full blood count; INR=international normalised ratio; TFT=thyroid function test; LFT=liver function test.

*Intraclass correlation coefficient.

†Reliability for practice with median number of patients at risk of triggering indicator.

‡Predicted prevalence in new practice with 95% prediction intervals using logistic regression model.

**Figure fig4:**
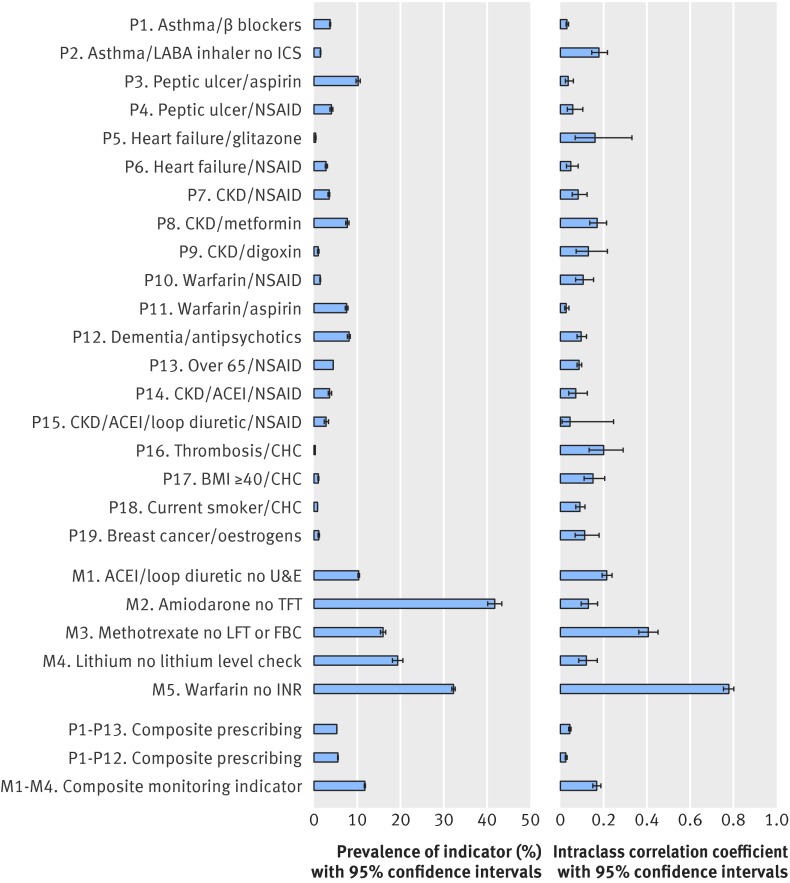
**Fig 4** Prevalence and intraclass correlation coefficients for individual and composite indicators of potentially hazardous prescribing in general practice

Overall, 49 927 of 949 552 (5.26%, 95% confidence interval 5.21% to 5.30%; table 2) patients triggered at least one prescribing indicator (P1-13). We excluded two indicators from the prescribing composite indicator (P1-P13, table 2) as they were nested within P7 and dealt with subgroup populations (P14-P15). Four were excluded because they were relevant only to women (P16-P19) and we wanted to evaluate the relation between sex and overall risk in a set of indicators that was relevant across both sexes. An alternative composite indicator included the indicators relevant to women (P1-P13 and P16-P19) and was triggered by 52 671 of 1 195 408 patients (4.41%, 4.37% to 4.44%; table 2).The corresponding composite monitoring indicator (M1-M4 excluding M5) was triggered by 21 501 of 182 721 patients (11.8%, 11.6% to 11.9%) (table 3). The decision to exclude M5 (warfarin without international normalised ratio (INR) testing) from the composite monitoring indicator was based on the large variation between practices (ICC=0.78) that was judged to reflect INR results not being recorded in CPRD (possibly because of different models of service delivery^20^) rather than real differences in INR testing. Previous work found that INR tests might be recorded in secondary, rather than primary, care records.^21^

Most patients triggered just one indicator (48 504 (97.1%), prescribing indicator; 21 399 (99.5%), monitoring indicator), but a few triggered two indicators (1253 (2.5%) prescribing indicator; 102 (0.5%) monitoring indicator) and 170 (0. 3%) patients triggered three or more prescribing indicator. Many practices had a prevalence of prescribing indicators over the 90th centile for just one prescribing indicator (158 (30%), P1-P13), but some practices were repeatedly over the 90th centile for different prescribing indicator; 100 (19%) practices were over the 90th centile twice, 79 (15%) three to four times, and 10 (2%) five to six times. For monitoring indicators, 166 (32%, M1-M4) practices had a prevalence over the 90th centile for just one indicator, 29 (6%) twice, and six (1%) three to four times. Monitoring indicators had larger ICCs than prescribing indicators; 0.17 (0.15 to 0.19) for the composite monitoring indicator and 0.04 (0.03 to 0.05) for the composite prescribing indicator (table 2, fig 4). There was low between-practice variation in prescribing β blockers to patients with asthma (ICC P1: 0.03, 0.02 to 0.04), aspirin to patients with peptic ulcer without gastroprotection (ICC P3: 0.04, 0.02 to 0.06), or co-prescription of warfarin and aspirin without gastroprotection (ICC P11: 0.03, 0.02 to 0.04) (table 2, fig 4). The reliability provides guidance about how confidently we can compare an individual practice with a known number of denominator patients with the prevalence over all practices. The implication is that for an indicator with lower ICC, higher numbers of denominator patients are required for a reliable comparison. For this reason, although the indicator related to prescribing β blockers to patients with asthma (P1) has a low ICC, the reliability is high because of the large denominator (number of patients diagnosed with asthma, table 2). Conversely prescribing amiodarone without a thyroid function test (M2) has a higher ICC but the small denominator (number of patients prescribed amiodarone) means that the reliability is low (table 3).

Tables 4 and 5 show the prevalence of patients triggering at least one prescribing indicator or monitoring indicator (composite indicators) by characteristics of patients and practices, as well as unadjusted and adjusted odds ratios derived from the multilevel mixed effects logistic regression model. Polypharmacy was strongly associated with increased risk of triggering the composite prescribing indicator (1.3% of patients with zero or one repeat medication compared with 12.1% of patients with 11 or more long term medications, table 4). The opposite was observed for the composite monitoring indicator where increasing repeat medications was associated with decreased risk (25.7% of patients with zero or one repeat medication compared with 8.8% of patients with 11 or more long term medications, table 5). Increasing age was associated with increasing prevalence of the composite prescribing indicator (1.5% for patients aged ≤50 compared with 7.0% for patients aged 71-80; table 4), though there was a protective effect for the oldest patients (4.6% for patients aged >80; table 4). Again the opposite trend was observed for the composite monitoring indicator as prevalence decreased with age (22.1% for patients aged ≤50 compared with 10.2% for patients aged 71-80; table 5). Women were more likely to trigger a monitoring indicator (12.4% compared with 10.8% in men; table 5) but less likely to trigger a prescribing indicator (5.1% compared with 5.5% in men; table 4). Practice level variables had much less influence than patient level variables; there was no effect of list size on composite prescribing or monitoring indicators. Practice level index of multiple deprivation showed a small significantly increased prevalence for the composite prescribing indicator for practices in more deprived areas in the unadjusted model (table 4). For the composite monitoring indicator there was no significant association with practice level index of multiple deprivation in the unadjusted model but the adjusted model showed small significant increases for the practices above the third quintile and below the fifth quintile but not the most deprived (above the fifth quintile (table 5). Northern Ireland had a significantly higher prevalence for the composite prescribing indicator relative to all other regions in the unadjusted model, and some regions in England were significantly lower (East of England, South Central, London, South West, South East Coast); none remained significant in the adjusted model (table 4). For the composite monitoring indicator North East England had significantly lower prevalence whereas South Central and London were significantly higher (table 5).

**Table 4 tbl4:** Prevalence of patients receiving at least one high risk prescribing indicator and multilevel unadjusted and adjusted odds ratios (P1 to P13)

Variable (No at risk)	% Prevalence (95% CI)	Odds ratio (95% CI)	
		Unadjusted	Adjusted
Age (years):			
18-50 (197 180)	1.5 (1.4 to 1.6)	1	1
51-60 (54 147)	4.9 (4.7 to 5.1)	3.28 (3.11 to 3.46)	1.95 (1.84 to 2.01)
61-70 (227 961)	7.2 (7.1 to 7.4)	5.20 (5.00 to 5.41)	3.57 (3.43 to 3.72)
71-80 (257 528)	7.0 (6.9 to 7.1)	4.98 (4.79 to 5.18)	2.84 (2.72 to 2.96)
>80 (212 736)	4.6 (4.5 to 4.7)	3.39 (3.26 to 3.54)	1.71 (1.64 to 1.78)
No of drugs on repeat prescription in past 12 months:			
0-1 (344 254)	1.3 (1.2 to 1.4)	1	1
2-4 (234 715)	4.8 (4.7 to 4.9)	3.70 (3.58 to 3.84)	3.39 (3.27 to 3.51)
5-7 (161 580)	7.1 (7.0 to 7.2)	5.64 (5.45 to 5.84)	4.99 (4.81 to 5.17)
8-10 (96 100)	9.4 (9.3 to 9.6)	7.71 (7.43 to 8.00)	6.95 (6.70 to 7.22)
>10 (112 903)	12.0 (11. 9 to 12.3)	10.17 (9.82 to 10.53)	9.40 (9.07 to 9.74)
Sex (18 missing):			
Male (431 280)	5.5 (5.4 to 5.6)	1	1
Female (518 254)	5.1 (5.0 to 5.2)	0.93 (0.91 to 0.95)	0.92 (0.90 to 0.93)
List size (fourths):			
<5700 (99 426)	5.5 (5.4 to 5.7)	1	1
5701-8600 (183 403)	5.8 (5.6 to 5.9)	1.05 (0.95 to 1.15)	1.03 (0.95 to 1.13)
8601-12 100 (265 806)	5.4 (5.3 to 5.5)	0.97 (0.88 to 1.07)	1.00 (0.92 to 1.09)
>12 100 (400 917)	4.9 (4.8 to 5.0)	0.90 (0.81 to 0.99)	0.99 (0.91 to 1.08)
Practice level index of multiple deprivation:			
1 least deprived (192 176)	5.0 (4.9 to 5.1)	1	1
2 (190 625)	5.0 (4.9 to 5.2)	1.04 (0.93 to 1.17)	1.00 (0.91 to 1.10)
3 (199 197)	5. 5 (5.4 to 5.6)	1.14 (1.01 to 1.27)	1.07 (0.97 to 1.18)
4 (195 128)	5.4 (5.3 to 5.5)	1.14 (1.02 to 1.28)	1.03 (0.94 to 1.14)
5 most deprived (172 426)	5.5 (5.4 to 5.6)	1.14 (1.02 to 1.28)	1.01 (0.92 to 1.12)
Region:			
North West (113 105)	5.9 (5.7 to 6.0)	1	1
North East (14 527)	5.0 (4.6 to 5.3)	0.75 (0.56 to 1.00)	0.76 (0.59 to 0.99)
Yorkshire/Humber (10 988)	7.9 (7.4 to 8.5)	1.34 (0.96 to 1.87)	1.35 (1.00 to 1.83)
East Midlands (6040)	5.0 (4.5 to 5.6)	0.81 (0.52 to 1.24)	0.81 (0.55 to 1.20)
West Midlands (89 358)	5.3 (5.1 to 5.4)	0.89 (0.77 to 1.03)	0.90 (0.79 to 1.02)
East of England (69 486)	4.9 (4.7 to 5.0)	0.80 (0.68 to 0.94)	0.89 (0.77 to 1.03)
South West (95 920)	5.0 (4.8 to 5.1)	0.80 (0.69 to 0.93)	0.83 (0.73 to 0.95)
South Central (119 249)	5.1 (5.0 to 5.2)	0.84 (0.73 to 0.96)	0.94 (0.83 to 1.07)
London (101 801)	4.3 (4.2 to 4.4)	0.69 (0.61 to 0.78)	0.77 (0.69 to 0.87)
South East coast (108 655)	4.8 (4.6 to 4. 9)	0.78 (0.68 to 0.89)	0.86 (0.76 to 0.97)
Northern Ireland (28 924)	7.5 (7.2 to 7.8)	1.26 (1.05 to 1.52)	1.15 (0.97 to 1.36)
Scotland (99 912)	5.5 (5.3 to 5.6)	0.92 (0.81 to 1.04)	0.92 (0.82 to 1.04)
Wales (91 587)	5.7 (5.6 to 5. 9)	0.97 (0.85 to 1.12)	0.90 (0.79 to 1.02)

**Table 5 tbl5:** Prevalence of patients receiving at least one high risk monitoring indicator and multilevel unadjusted and adjusted odds ratios (M1 to M4)

Variable (No at risk)	% Prevalence (95% CI)	Odds ratio (95% CI)	
		Unadjusted	Adjusted
Age (years):			
18-50 (3979)	22.1 (20.9 to 23.4)	1	1
51-60 (4070)	19.9 (18.8 to 21.1)	0.84 (0.75 to 0.94)	0.92 (0.82 to 1.04)
61-70 (5317)	19.0 (18.0 to 20.1)	0.80 (0.72 to 0.89)	0.97 (0.87 to 1.08)
71-80 (67 538)	10.2 (10.0 to 10.5)	0.36 (0.33 to 0.39)	0.44 (0.40 to 0.48)
>80 (101 817)	11.7 (11.5 to 11.9)	0.42 (0.39 to 0.46)	0.53 (0.49 to 0.58)
No of drugs on repeat prescription in past 12 months:			
0-1 (10 490)	25.7 (23.5 to 28.1)	1	1
2-4 (23 404)	18.3 (17.9 to 18.8)	0.64 (0.56 to 0.72)	0.68 (0.60 to 0.78)
5-7 (43 957)	13.3 (12.9 to 13.6)	0.42 (0.37 to 0.48)	0.47 (0.41 to 0.53)
8-10 (40 993)	10.7 (10.4 to 11.0)	0.33 (0.29 to 0.37)	0.36 (0.32 to 0.42)
>10 (63877)	8.8 (8.6 to 9.0)	0.26 (0.23 to 0.30)	0.29 (0.25 to 0.33)
Sex:			
Male (75 210)	10.8 (10.6 to 11.1)	1	1
Female (107 511)	12.4 (12.2 to 12.6)	1.17 (1.13 to 1.20)	1.16 (1.12 to 1.20)
List size (fourths):			
<5700 (20 378)	13.3 (12.8 to 13.8)	1	1
5701-8600 (36 770)	12.1 (11.8 to 12.5)	0.97 (0.79 to 1.19)	0.97 (0.79 to 1.19)
8601-12 100 (52 639)	11.6 (11.4 to 11.9)	0.93 (0.76 to 1.15)	0.90 (0.73 to 1.11)
>12 100 (72 934)	11.2 (11.0 to 11.5)	0.93 (0.76 to 1.15)	0.95 (0.77 to 1.18)
Practice level index of multiple deprivation:			
1 least deprived (34 890)	10.8 (10.5 to 11.2)	1	1
2 (39 087)	11.9 (11.6 to 12.3)	1.16 (0.91 to 1.46)	1.14 (0.90 to 1.44)
3 (39 699)	12.3 (12.0 to 12.6)	1.25 (0.99 to 1.58)	1.37 (1.09 to 1.73)
4 (37 944)	12.3 (11.9 to 12.6)	1.25 (0.99 to 1.58)	1.32 (1.04 to 1.67)
5 most deprived (31 101)	11.3 (11.0 to 11.7)	1.11 (0.88 to 1.41)	1.27 (0.99 to 1.62)
Region:			
North West (20 587)	10.6 (10.2 to 11.1)	1	1
North East (2744)	5.8 (5.0 to 6.8)	0.43 (0.22 to 0.82)	0.43 (0.22 to 0.83)
Yorkshire/Humber (1975)	12.5 (11.1 to 14.0)	1.40 (0.67 to 2.89)	1.38 (0.66 to 2.88)
East Midlands (1204)	13.9 (12.0 to 15.9)	0.93 (0.36 to 2.37)	0.95 (0.37 to 2.47)
West Midlands (17 801)	10.8 (10.3 to 11.2)	1.09 (0.80 to 1.49)	1.10 (0.81 to 1.50)
East of England (12 761)	10.8 (10.3 to 11.4)	1.08 (0.77 to 1.53)	1.08 (0.76 to 1.54)
South West (18 662)	8.4 (8.0 to 8.8)	0.75 (0.55 to 1.02)	0.70 (0.51 to 0.96)
South Central (22 679)	14.0 (13.6 to 14.5)	1.37 (1.02 to 1.84)	1.37 (1.00 to 1.86)
London (18 328)	14.6 (14.0 to 15.0)	1.52 (1.16 to 1.99)	1.47 (1.11 to 1.94)
South East Coast (22 230)	12.0 (11.6 to 12.5)	1.23 (0.92 to 1.64)	1.17 (0.87 to 1.57)
Northern Ireland (6276)	10.2 (9.5 to 11.0)	1.01 (0.68 to 1.51)	1.03 (0.68 to 1.56)
Scotland (19 365)	14.4 (14.0 to 14.9)	1.16 (0.88 to 1.54)	1.14 (0.86 to 1.51)
Wales (18 109)	10.6 (10.2 to 11.0)	0.89 (0.66 to 1.21)	0.89 (0.65 to 1.20)

We excluded the indicators relevant only to women from the composite prescribing indicator (P1-P13) to allow meaningful comparisons between sexes; however, this is not to say that the indicators relevant to women are less important. Table A in appendix 2 shows the odds ratios derived from the multilevel mixed effects logistic regression model for the composite prescribing indicator that included the indicators for women (P1-P13 plus P16-P19). There were few differences between the predictors for these two composite prescribing indicators (P1-P13 *v* P1-P13 plus P16-P19) except, as expected, on sex and age (as a result of combined hormone contraceptive prescribing occurring mostly in the younger age group). Additionally as both composite prescribing and monitoring indicators included a single indicator with a large denominator that could have driven the observations, we ran the model separately for P1-P12 and P13 (the indicator related to prescribing NSAID to a patient aged >65) and M1 (the indicator related to prescribing angiotensin converting enzyme inhibitor without urea and electrolyte tests) and M2-M4. Tables B-E in appendix 2 show the results. For the composite prescribing indicator the effect size of polypharmacy in the adjusted model was driven largely by P1-P12. The likelihood of triggering P13 (patients aged >65 prescribed NSAIDs) increased with two to four repeated prescriptions but did not increase further with increasing number of prescriptions (table B and C in appendix 2). For the monitoring indicator, the composite was strongly influenced by M1: exclusion of M1 from the composite (M2-M4) reversed the direction of sex, with women becoming less likely to trigger the composite monitoring indicator (tables D and E in appendix 2). Furthermore, the decrease in prevalence with increasing repeat medications occurred in the two to four repeated prescriptions group and did not increase further with increasing number of prescriptions.

Figure 5 shows the prevalence of composite prescribing and monitoring indicators for each practice. A high prevalence for the composite prescribing indicator does not predict a high prevalence for the composite monitoring indicator or vice versa.

**Figure fig5:**
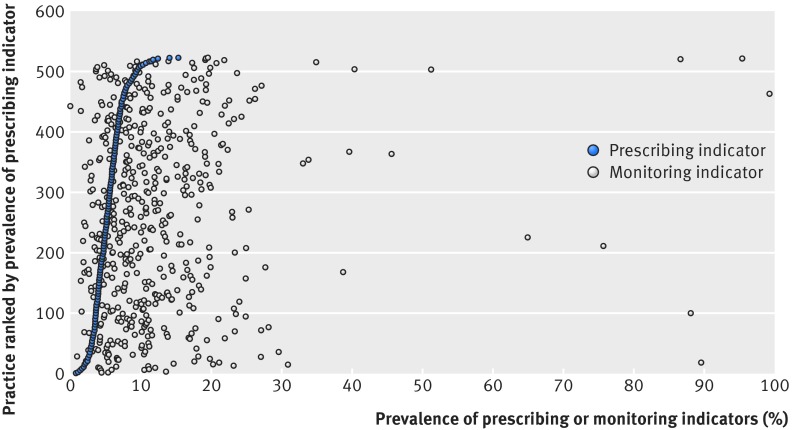
**Fig 5** Prevalence of composite prescribing and monitoring indicators in individual practices ranked by prescribing indicators

## Discussion

### Principal findings

There is a high variation in prevalence between general practices for indicators of potentially hazardous prescribing and missed monitoring tests. It is unlikely that variation in case mix could explain the large differences between practices, though prescribing indicators can occur for valid clinical reasons. There is no parallel argument that a monitoring indicator might occur for clinical reasons but patient behaviour might be an important factor. Furthermore, monitoring indicators can occur because of poor recording of test results, whereas most prescribing is systematically recorded through the electronic prescribing system and is known to be accurate (reflected in the larger intraclass correlation coefficients (ICC) for monitoring indicators).^22^ Many practices, however, do have zero prevalence for some indicators, and these results might help to set reasonable expectations for individual practices.

An important question is whether or not it is appropriate to use these indicators to compare prescribing safety between practices. The ICC suggests that the differences between practices are an important source of variation in the prevalence of the indicators, especially for monitoring indicators, but the ICC is a statistical concept and further work is needed to estimate how often indicators result from oversight or error rather than a clinical decision. The reliability estimates (derived from the ICC) show that some practices have insufficient numbers of patients in the denominator for reliable prevalence estimates for some indicators and these practices should not be compared with other practices. For the composite indicators, however, the reliability was good across all practices (>0.8, appendix 1), suggesting that comparisons between all practices should focus on the composite indicators.

High levels of variation between practices might also reflect differences in service organisation—for example, M5 (prescribed warfarin and no INR) has an ICC of 0.78, possibly because in some practices that prescribe warfarin the INR results might be recorded outside the practice electronic record system and are not uploaded to CPRD.^20^ ^21^ One way to identify practices with high risk prescribing might be to look for those with a consistently higher prevalence across several different indicators; 17% of practices had prevalence over the 90th centile for three or more of the 13 prescribing indicators (P1-P13) and 7% of practices for two or more of the four monitoring indicators (M1-M4).

If a lower prevalence of these indicators is a measure of good practice then we would expect consistency between prescribing and monitoring indicators within practices. An alternative hypothesis is that since prescribing indicators are the culmination of a series of actions, whereas monitoring indicators result from inaction on the part of the practice or the patient, practices with a lower prevalence of monitoring indicators would have a higher prevalence of prescribing indicators. In fact there was no clear relation between the two types of indicator within practices (fig 5). There were differences, however, in the type of patient triggering prescribing or monitoring indicators. Older patients and those receiving multiple repeat prescriptions had the highest risk of triggering the composite prescribing indicator, whereas younger patients with fewer repeat prescriptions had a higher risk of triggering the composite monitoring indicator. The implication is that different types of intervention might be required to deal with the two types of high risk prescribing.

Practice level variables had much smaller effects than patient level variables; patients attending practices in more deprived areas were more likely to trigger the composite prescribing indicator, possibly reflecting more prescribing within the practice leading to higher potential for a prescribing indicator. For the composite monitoring indicator, however, modest significant effects of practice level deprivation were seen only in the adjusted model, suggesting that patients attending practices in deprived areas have no increased likelihood of missing monitoring tests. The definition of the composite prescribing indicator did not substantially alter the predictions made by the model but M1 (prescribing ACEI without urea and electrolyte monitoring) was the major driver for a monitoring indicator and, given the number of patients at risk, might be a better marker of prescribing safety.

In this group of 526 practices, about 5% of patients with the potential to trigger a prescribing indicator did so and about 12% triggered a monitoring indicator. Whether or not these prevalence estimates might be generalised to the UK depends on how representative the practices participating in CPRD are in terms of characteristics of patients and practices. Practices in CPRD are slightly larger than the UK average (this might not cause bias as list size was not a significant predictor of prevalence) but are representative in terms of patient level deprivation and ethnicity.^23^ All CPRD practices in this study use Vision software to manage their patient data, and the choice of software has been associated with differences in recorded clinical data.^24^ The regional differences in the choice of software impact on the geographical representativeness of CPRD; Yorkshire, Humberside, and North East England are under-represented.^23^ Given this, it is reassuring that there are few regional differences in the prevalence of the indicator. The lower prevalence of monitoring indicator in the North East and higher prevalence in London might simply reflect selection bias given the small number of practices. While there might be an argument for generalising these prevalence estimates to the UK, we need to be more cautious in making the same case for the reliability estimates. The variability between practices, from which both the ICC and reliability are derived, might be specific to this group of practices and hence a generalisation to the whole of the UK primary care might be difficult to justify.

### Comparison with other studies

It is useful to compare these findings with studies in other UK practices. Consideration, however, must be given to the date of analysis as the quality of electronic data has improved over recent years. The audit date chosen here is a compromise between being as recent as possible but also allowing time for most practices to have had their data uploaded. The prevalence of these indicators was not sensitive to the choice of audit date, suggesting that seasonality and the Quality Outcomes Framework (QOF) return date had little impact. (The QOF incentive scheme provides UK GPs with financial incentives related to performance indicators and practices can review their patient records before the annual return in April^19^). The only exception was M4 (prescribed lithium without lithium level check), where the increase in lithium level tests during January might relate to preparation for the QOF return or the end of the financial year. The PINCER trial found similar prevalences for P1, P4, P16, M1, and M2 (using identical definitions) in 72 practices in Nottinghamshire, Staffordshire, and Cheshire during 2006-07.^10^ There were, however, some differences; the prevalence for M3 (methotrexate without full blood count or liver function test) was 35-42% in the PINCER trial but 16% in the CPRD; and for M5 (warfarin and INR) 6-7% in PINCER and 32% in the CPRD. (CPRD data might not capture all INR test results as discussed above, whereas the PINCER trial excluded practices that were known to undertake their own INR monitoring from this indicator). A study of 315 practices in Scotland used different indicator definitions measured relative to 31 March 2007; there were similar findings for the comparable indicators.^9^ The prescription of gastroprotectants, however, was treated differently. We excluded patients from the denominator if they had been prescribed gastroprotection, whereas the Scottish indicator excluded them from the numerator.^9^ If the same definition as the Scottish indicator for prescribing NSAIDS to elderly patients was applied, the prevalence was similar between the two datasets (data available from authors).

### Limitations of the study

While it is relatively straight forward for GPs within the UK to compare their practice with these results, it is a weakness of this study that international comparisons with these data would be challenging. Such comparisons would require access to routinely collected patient data (even CPRD might be missing some data—for example, INR tests^20^ ^21^). In many countries healthcare data are collected primarily for payment or insurance purposes and the clinical utility of the indicators, and their representativeness of the general population, might be uncertain.

### Conclusions and policy implications

These results emphasise the need to give due consideration to the risks of prescribing multiple medications and the importance of regular medication reviews, especially for patients with multiple morbidity. Here we provide a baseline prevalence from which to determine whether or not prescribing safety is improving.

These findings are also relevant to policy makers looking for ways to compare and potentially reward practices with respect to prescribing safety, although careful consideration needs to be given to any such initiative. Prescribing tends to be an individual rather than practice responsibility and prescriptions might be initiated in secondary care rather than by the GP. Further work would also be needed to estimate clearly how frequently indicators result from a clinical decision rather than an oversight or error and which indicators pose the most risk to the patient.

**What is already known on this topic**

Prescribing safety indicators have been developed to identify patients at increased risk of hazardous prescribing in primary care
Although these prescribing safety indicators have been investigated in experimental settings, they have not been assessed in a large UK-wide primary care database

**What this study adds**

Variation in the prevalence of potentially high risk prescribing and lack of appropriate monitoring tests between practices was high, even after adjustment for patient and practice level variables, pointing towards important targets for improving patient safety in primary care
In a broadly representative sample of 526 UK general practices, about 5% of patients at risk were found to have received a potentially inappropriate prescription and about 12% had no record of appropriate monitoring
Older patients and those receiving multiple repeat prescriptions had the highest risk of triggering a prescribing safety indicator, whereas younger patients with fewer repeat prescriptions had higher risk of triggering a monitoring indicator

## Notes

This study is based on data from the Clinical Practice Research Datalink obtained under licence from the UK Medicines and Healthcare Products Regulatory Agency. The interpretation and conclusions contained in this paper are those of the authors alone.

Ethical approval: The study was approved by the independent scientific advisory committee (ISAC) for Clinical Practice Research Datalink research (reference No 14_049R). No further ethics approval was required for the analysis of the data.

Transparency declaration: SJS affirms that this manuscript is an honest, accurate, and transparent account of the study being reported; that no important aspects of the study have been omitted; and that any discrepancies from the study as planned have been explained.

Data sharing: Clinical Practice Research Datalink data cannot be shared because of licensing restrictions.

## Notes

Cite this as: *BMJ* 2015;351:h5501
