# Supplementary material for: Examining variations in prescribing safety in UK general practice: cross sectional study using the Clinical Practice Research Datalink
Source: BMJ. 2015 Nov 3;351:h5501. doi: 10.1136/bmj.h5501 (PMC4632209; doi:10.1136/bmj.h5501)

Online Appendix 1. Results for composite and individual prescribing indicators

Prescribing composite indicator P1-P13

Denominator – group at risk	Numerator – increased risk identified
All patients at risk of a prescribing indicator P1-P13	Had at least one prescribing indicator

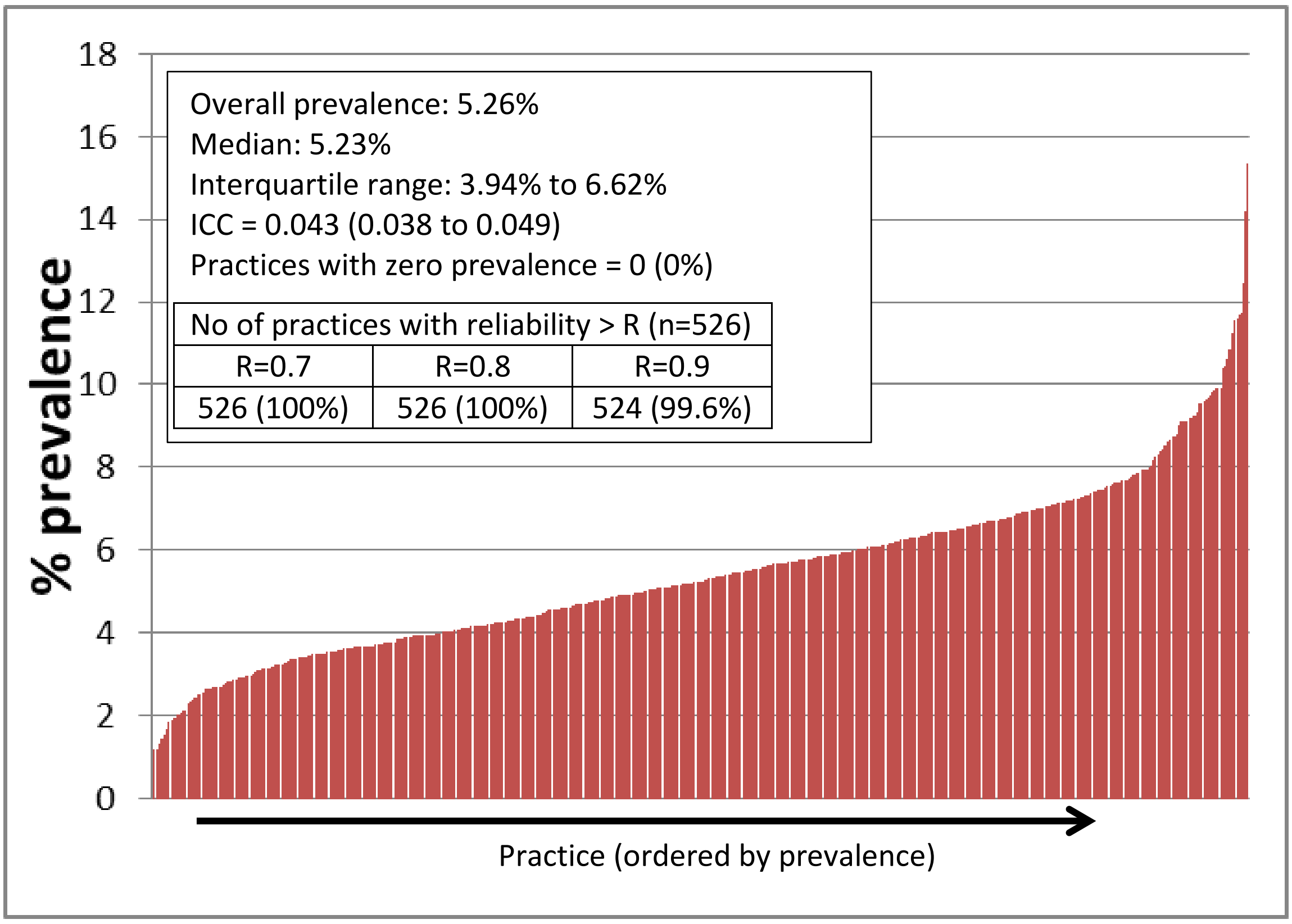

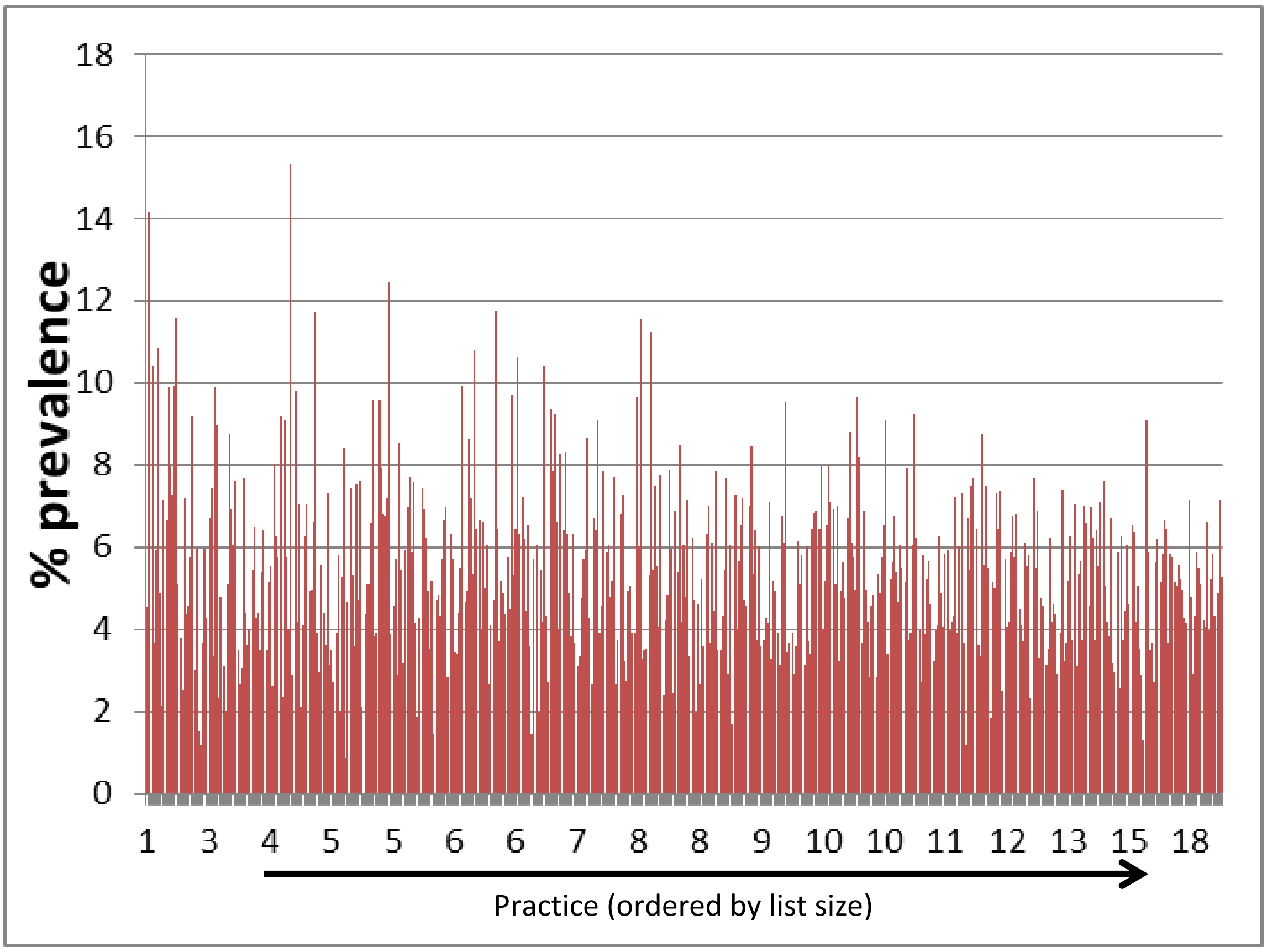

Prescribing composite indicator P1-P12

Denominator – group at risk	Numerator – increased risk identified
All patients at risk of a prescribing indicator P1-P12	Had at least one prescribing indicator

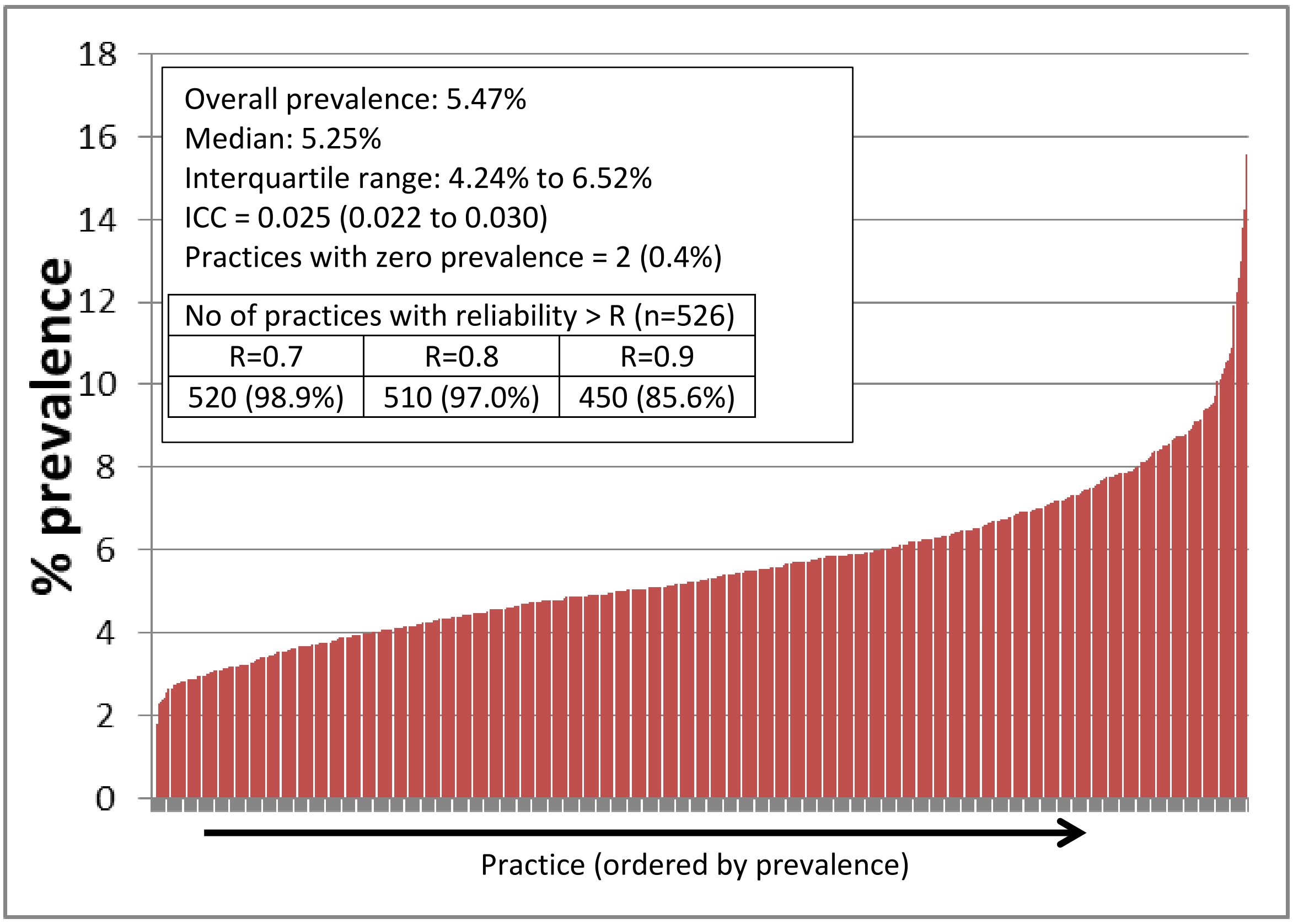

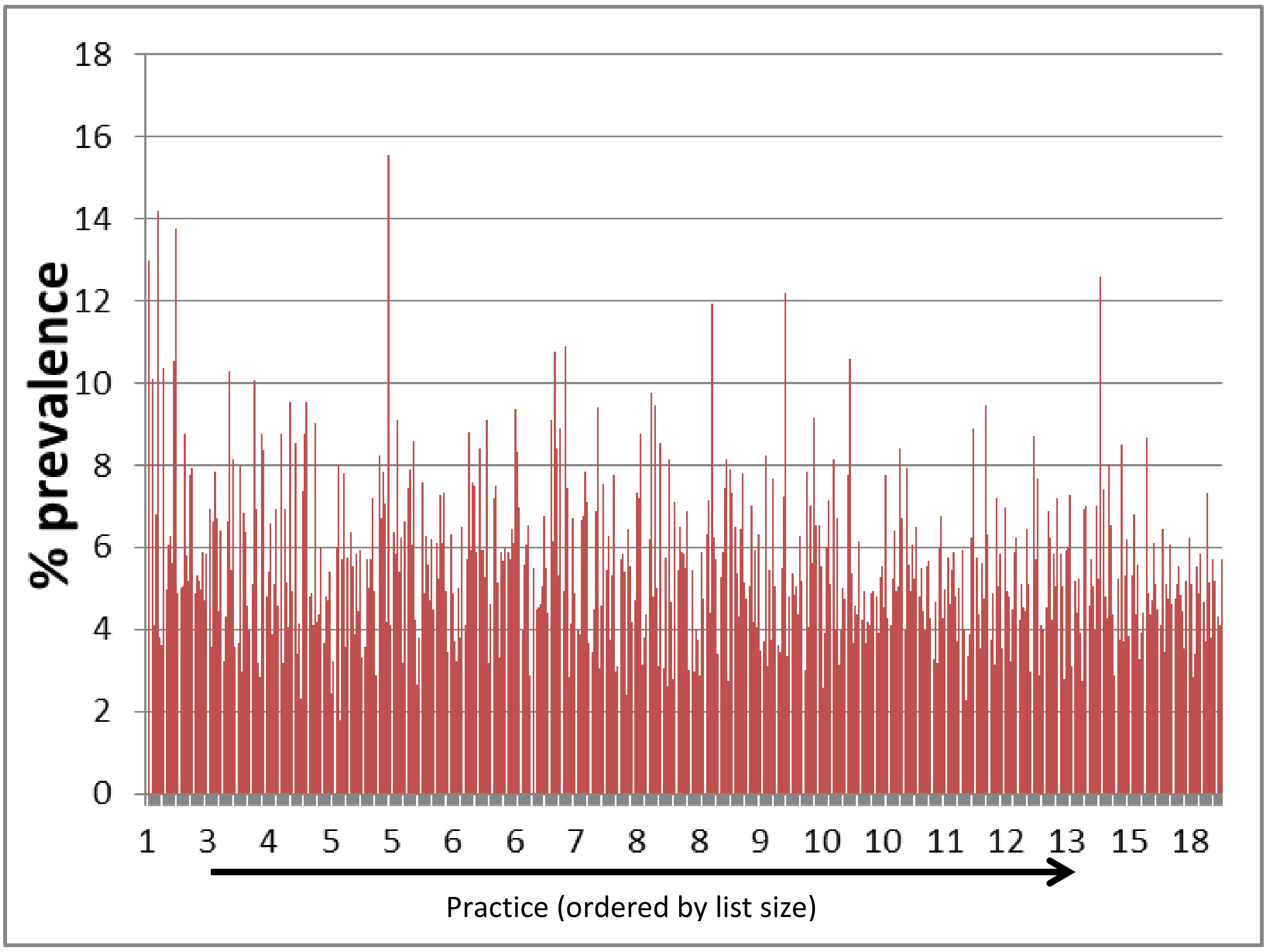

Monitoring composite indicator M1-M4

Denominator – group at risk	Numerator – increased risk identified
All patients at risk of a monitoring indicator M1-M4	Had at least one monitoring indicator

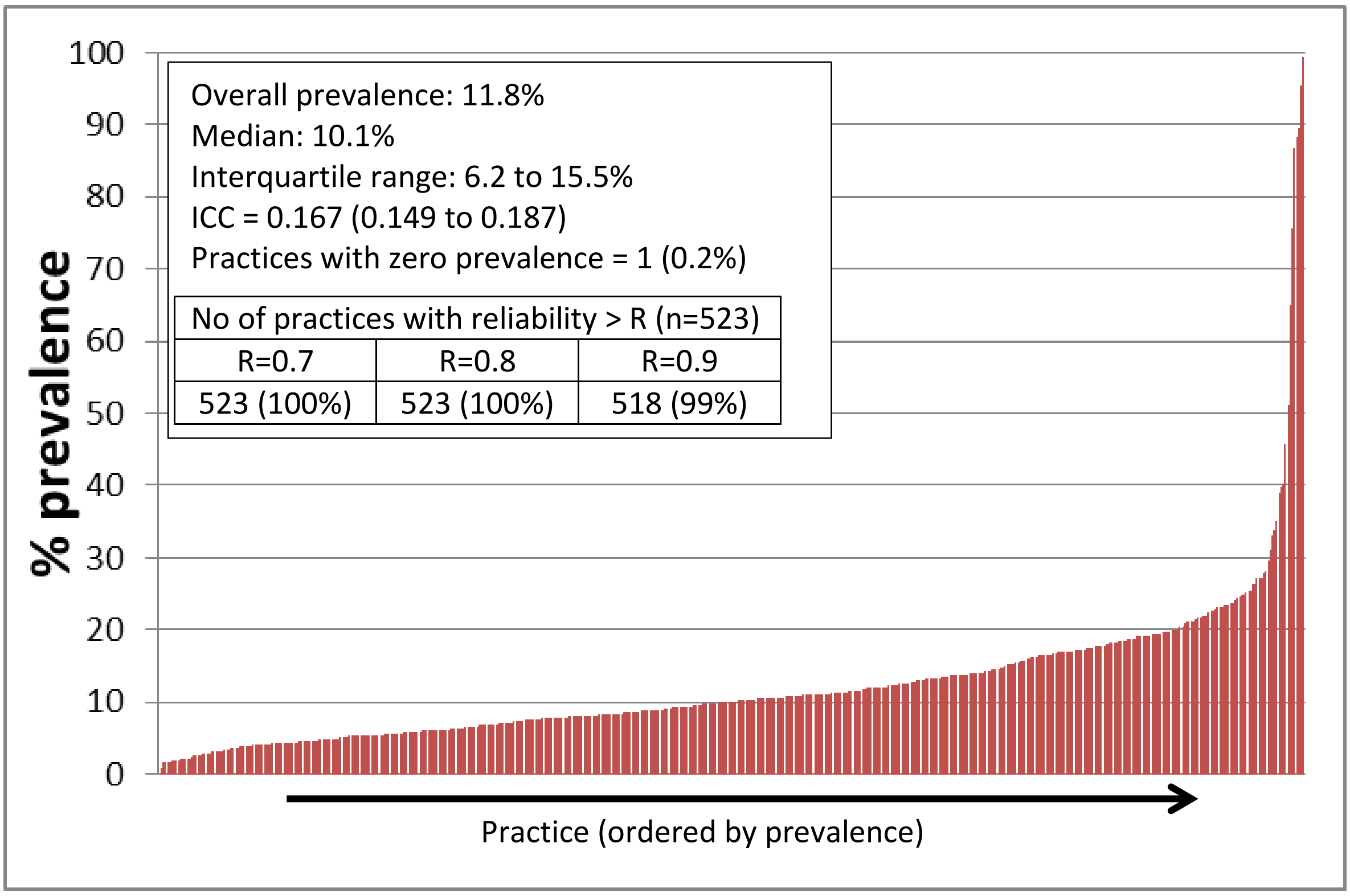

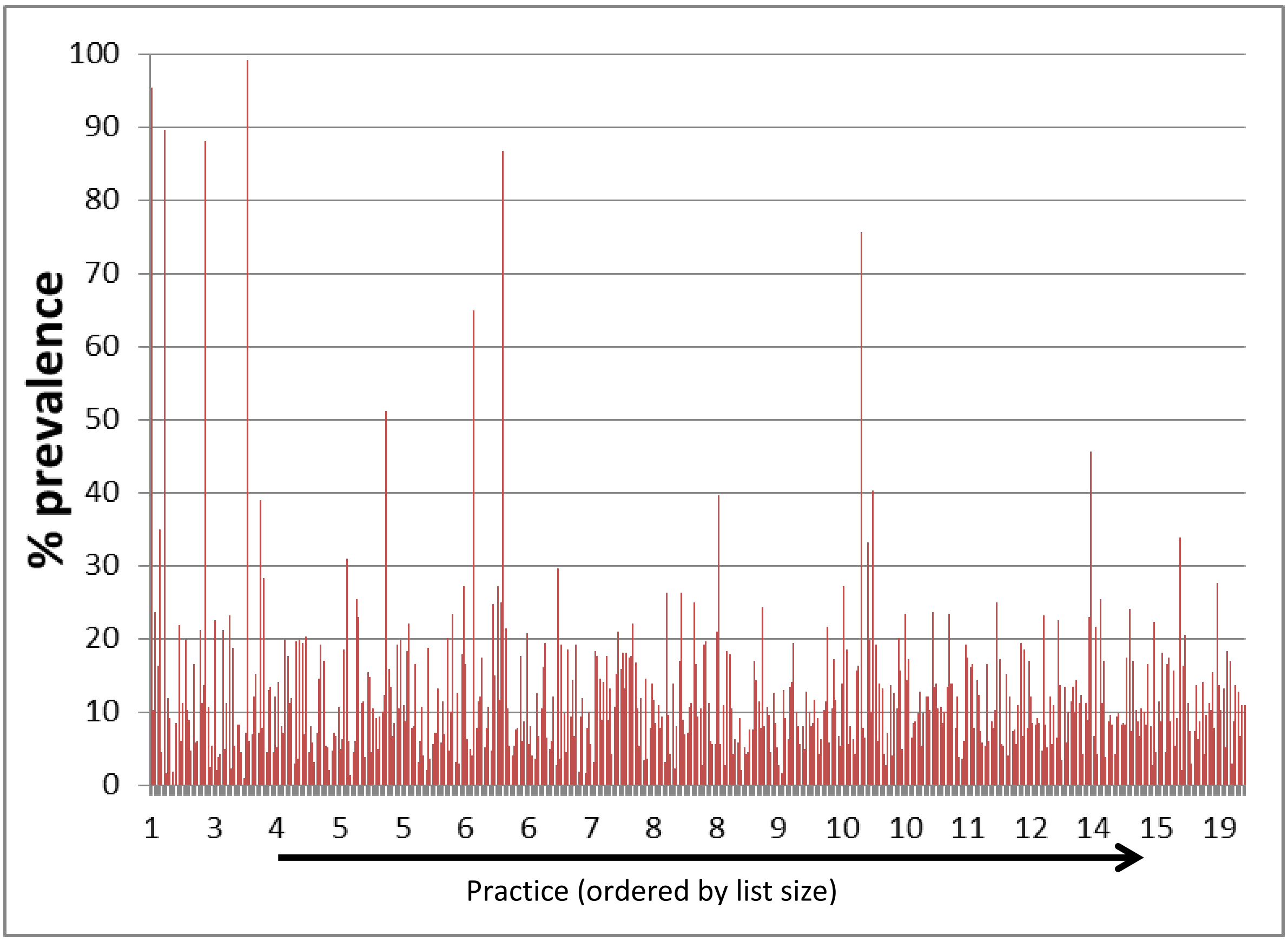

P1. Asthma and prescribed β-blockers

Denominator – group at risk	Numerator – increased risk identified
Has asthma read code dated at least 6 months before audit date and no asthma resolved code since latest asthma code	Had a β-blockers prescription within the 6 months leading up to the audit date

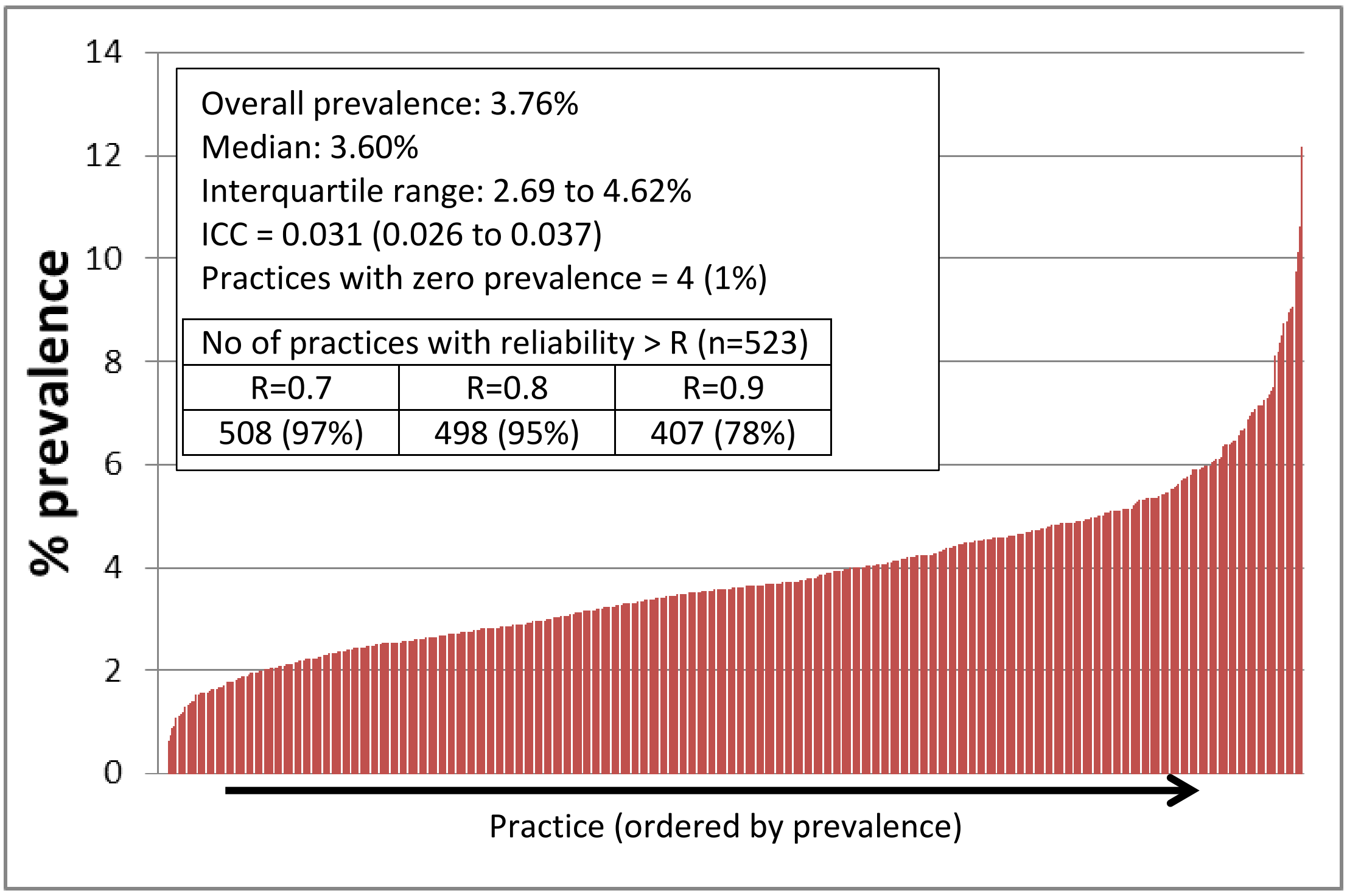

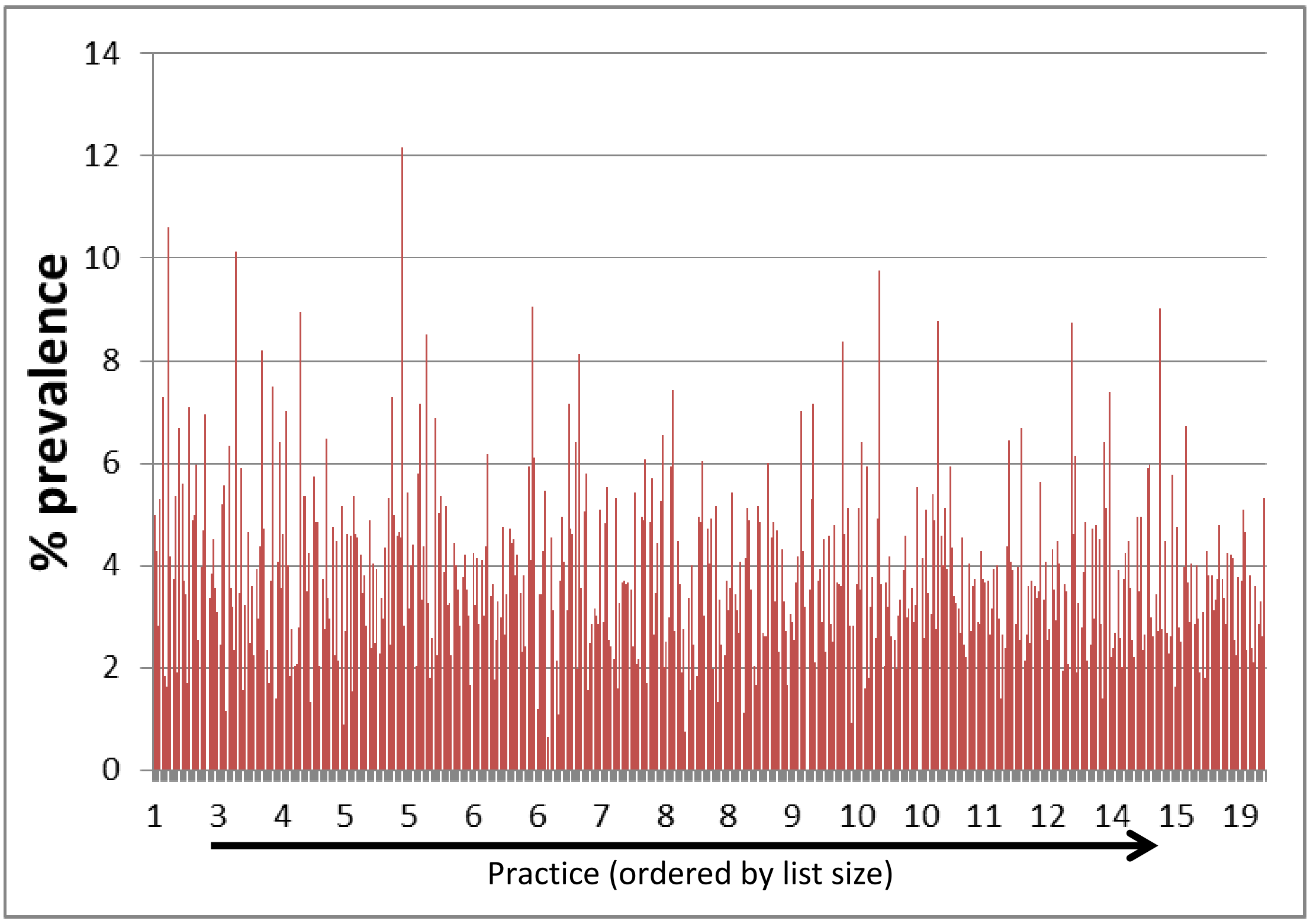

P2. Asthma and prescribed LABA without ICS

Denominator – group at risk	Numerator – increased risk identified
Has asthma read code dated at least 3 months before audit date & prescribed a long-acting beta-2 agonist inhaler (LABA) within the 3 months leading up to the audit date	Not prescribed an inhaled corticosteroid (ICS) or a combined LABA-ICS within the 3 months leading up to the audit date

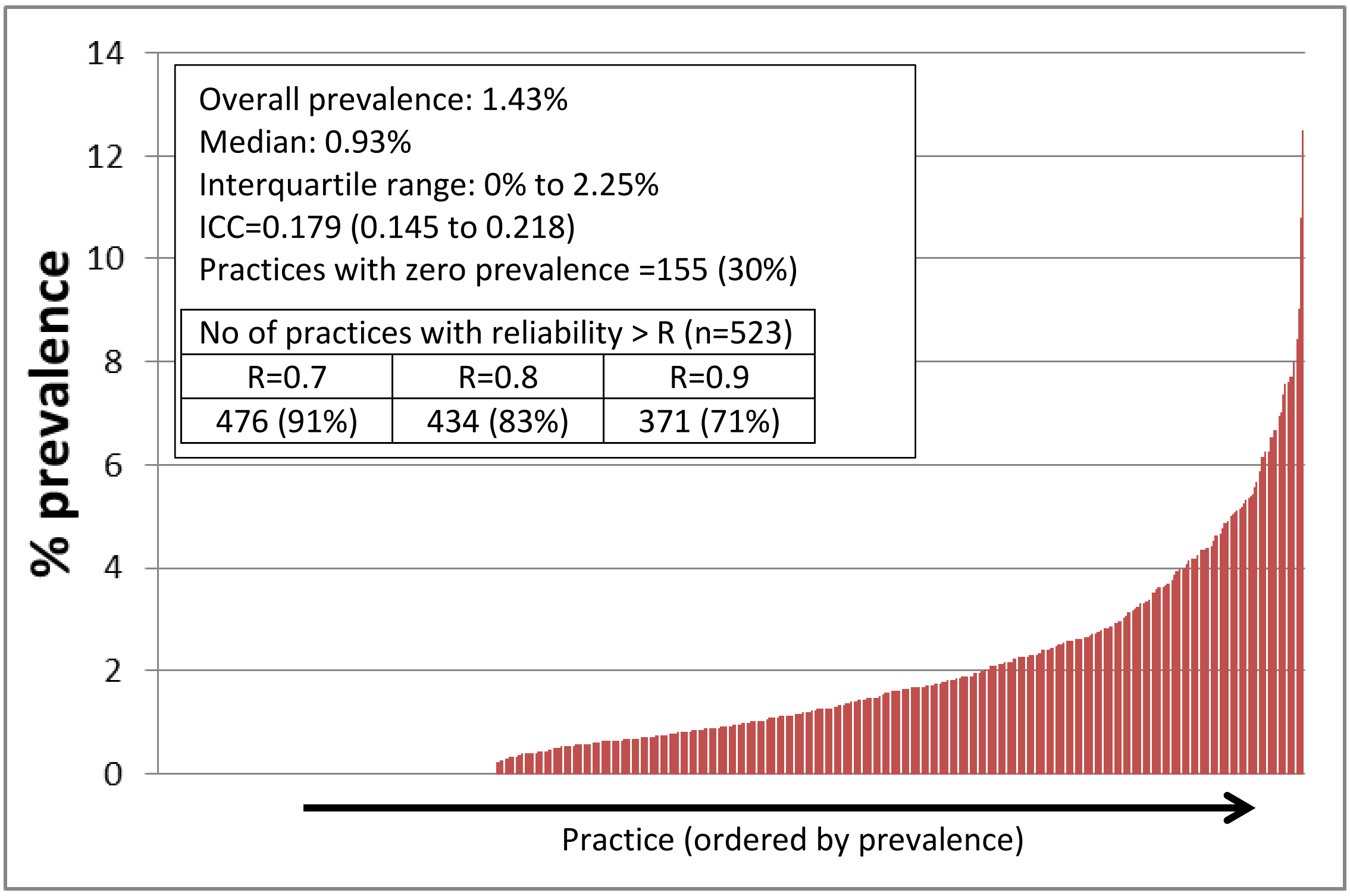

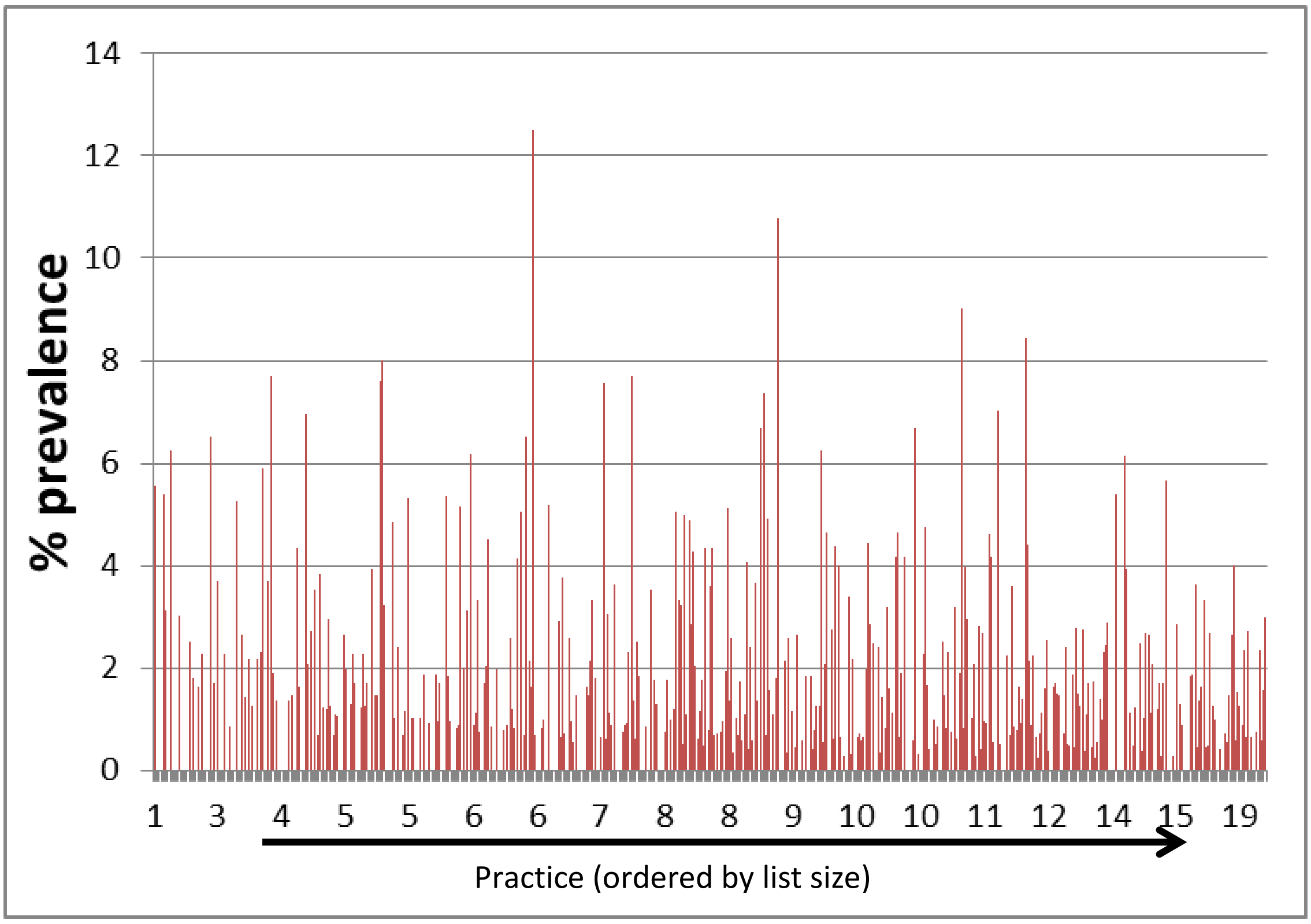

P3. Peptic ulcer, no gastroprotection and prescribed aspirin or clopidogrel

Denominator – group at risk	Numerator – increased risk identified
Has read code for peptic ulcer or peptic surgery dated at least 6 months before audit date and not prescribed PPI, misoprostol or H2 antagonist within the 6 months leading up to the audit date	Prescribed aspirin or clopidogrel within the 6 months leading up to the audit date

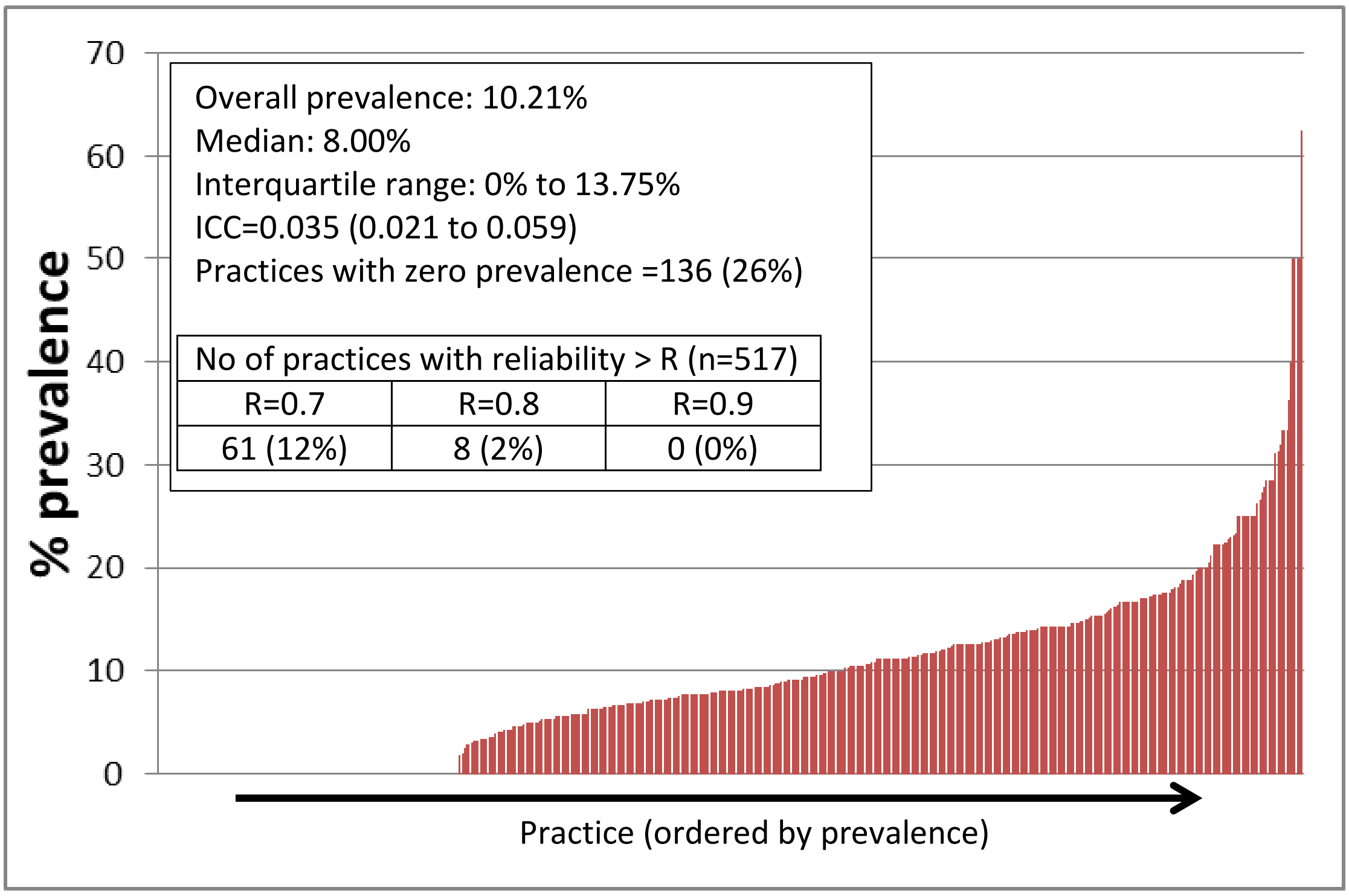

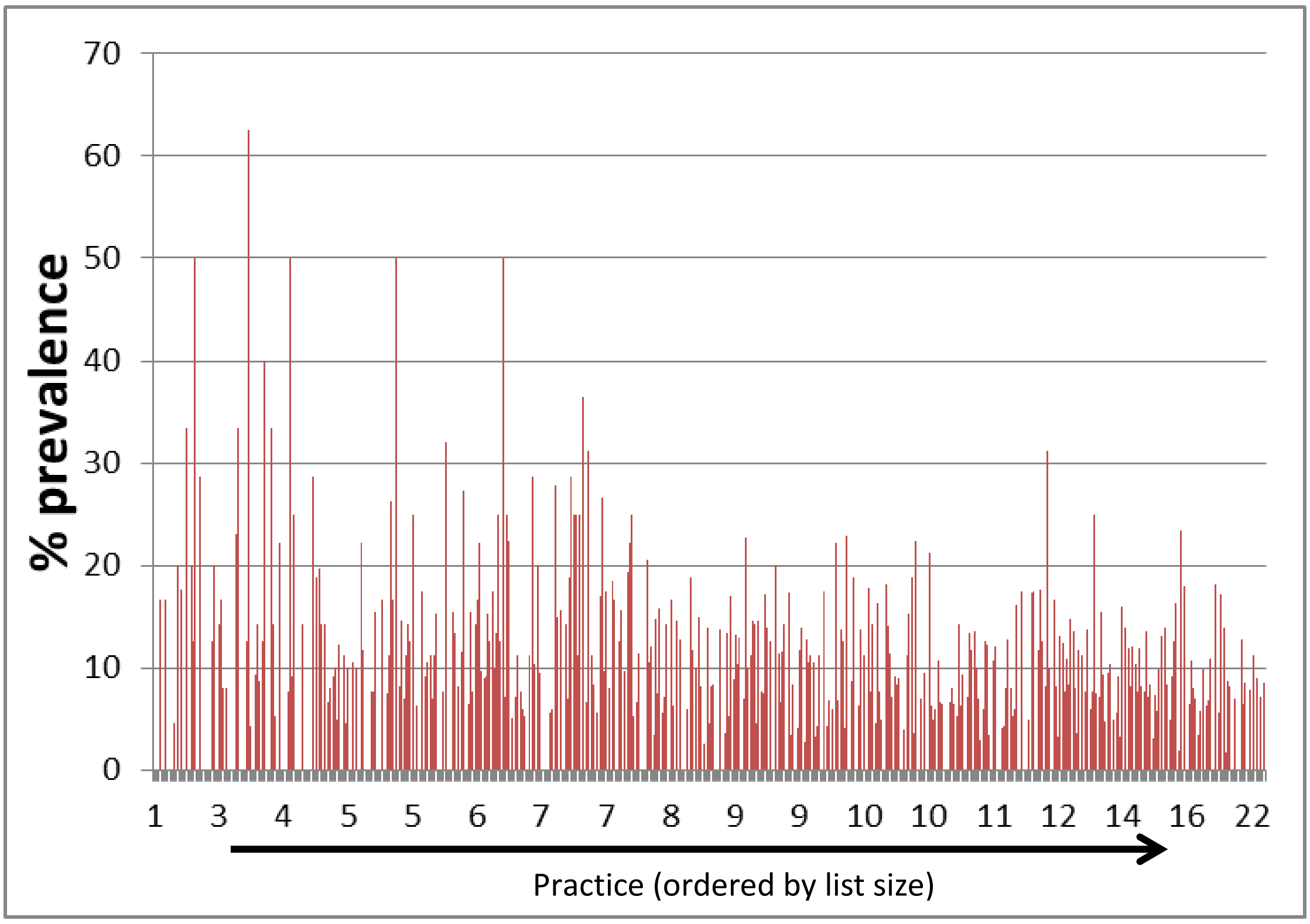

P4. Peptic ulcer, no gastroprotection and prescribed NSAID

Denominator – group at risk	Numerator – increased risk identified
Has read code for peptic ulcer or peptic surgery dated at least 6 months before audit date and not prescribed PPI, misoprostol or H2 antagonist within the 6 months leading up to the audit date	Prescribed NSAID within the 6 months leading up to the audit date

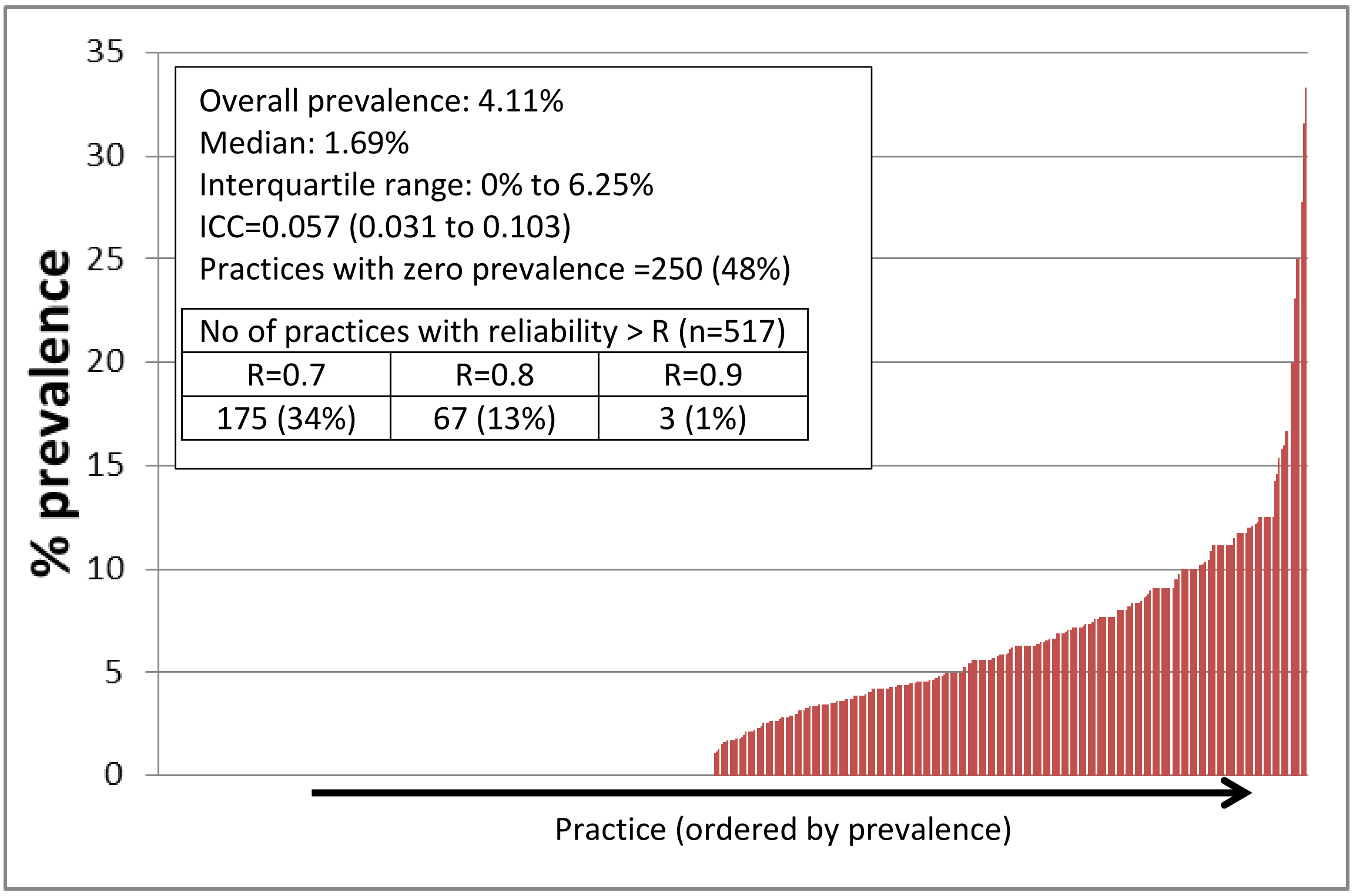

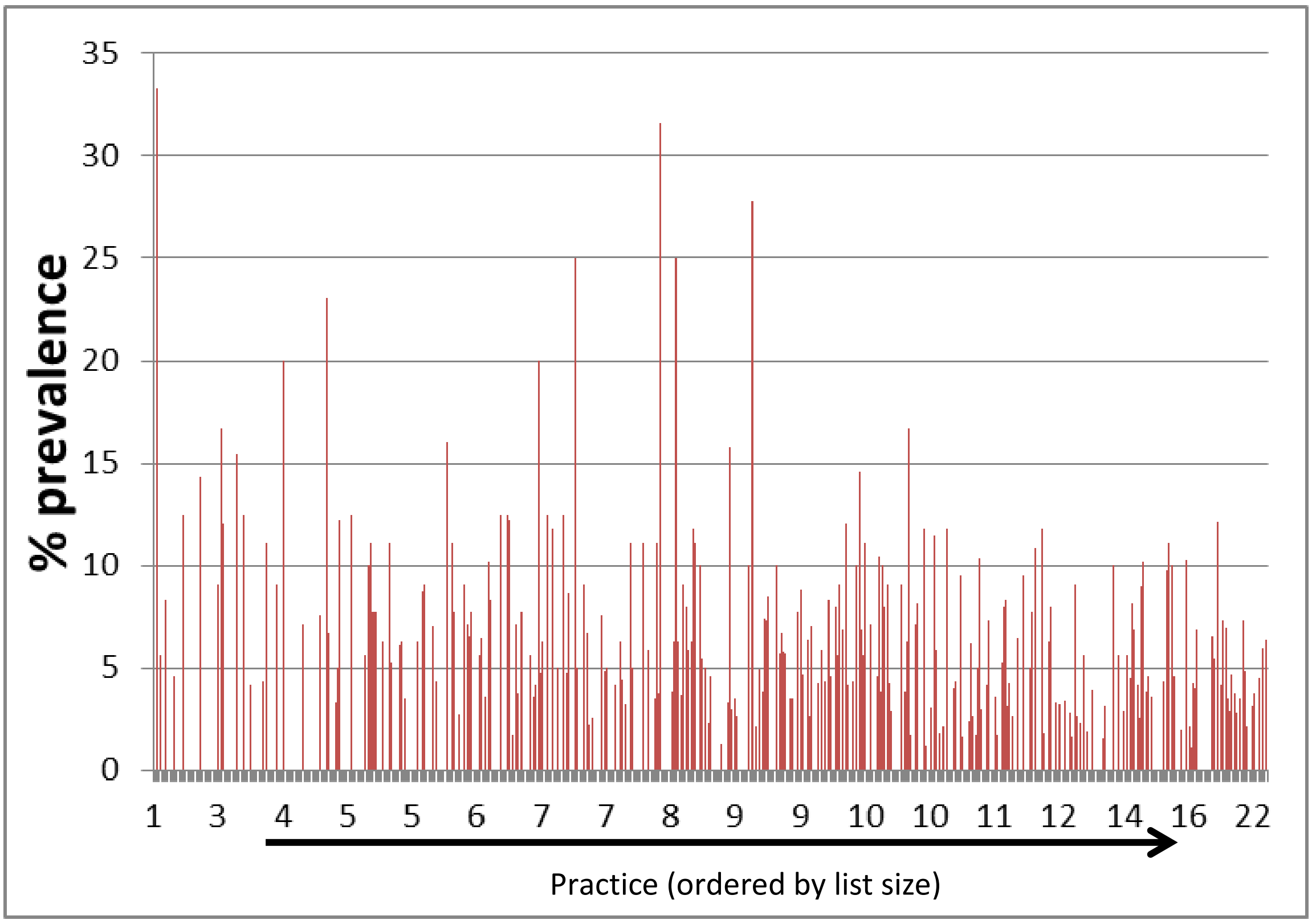

P5. Heart failure and prescribed glitazone

Denominator – group at risk	Numerator – increased risk identified
Has read code for heart failure dated at least 3 months before audit date	Prescribed glitazone within the 3 months leading up to the audit date

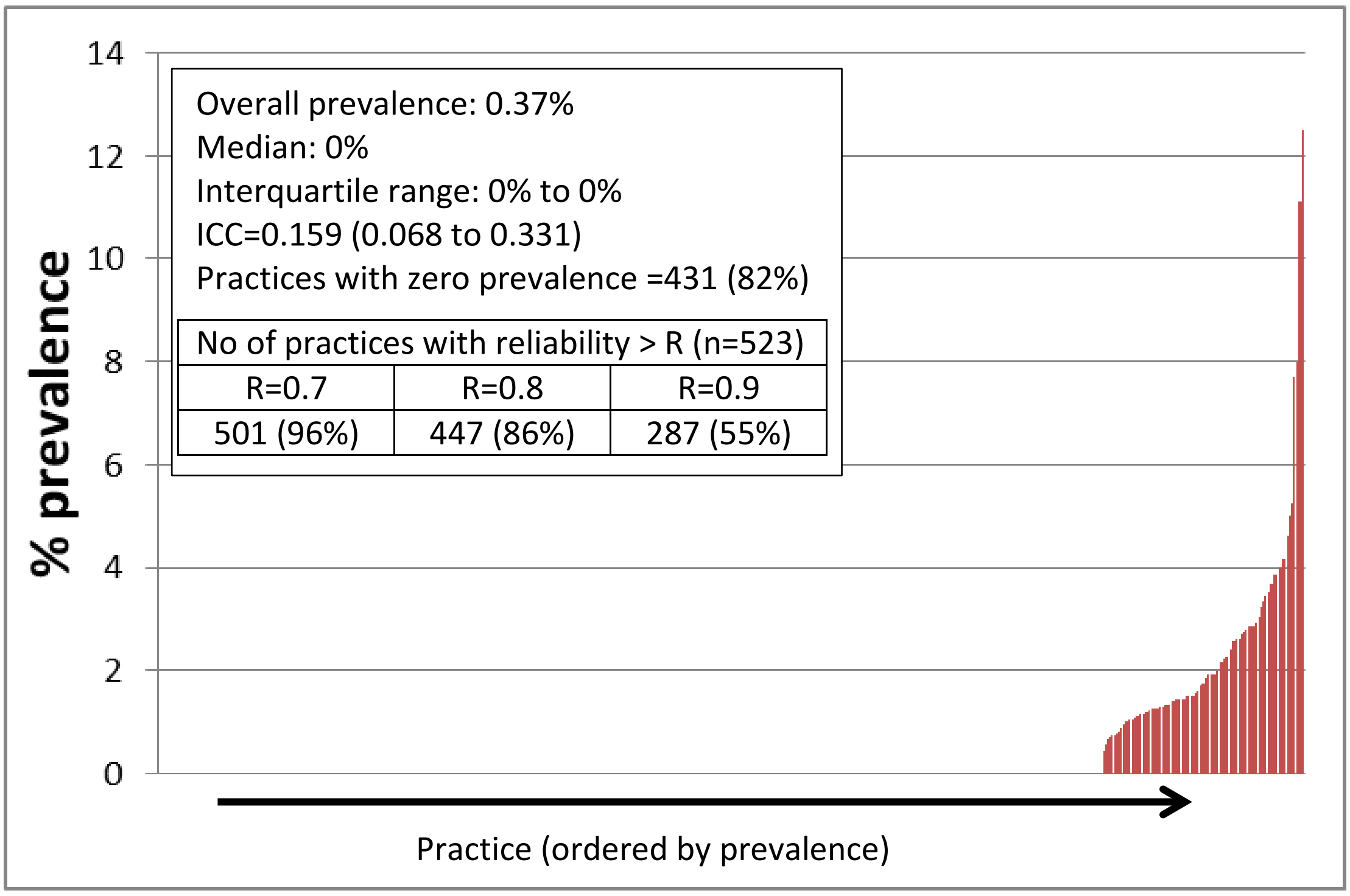

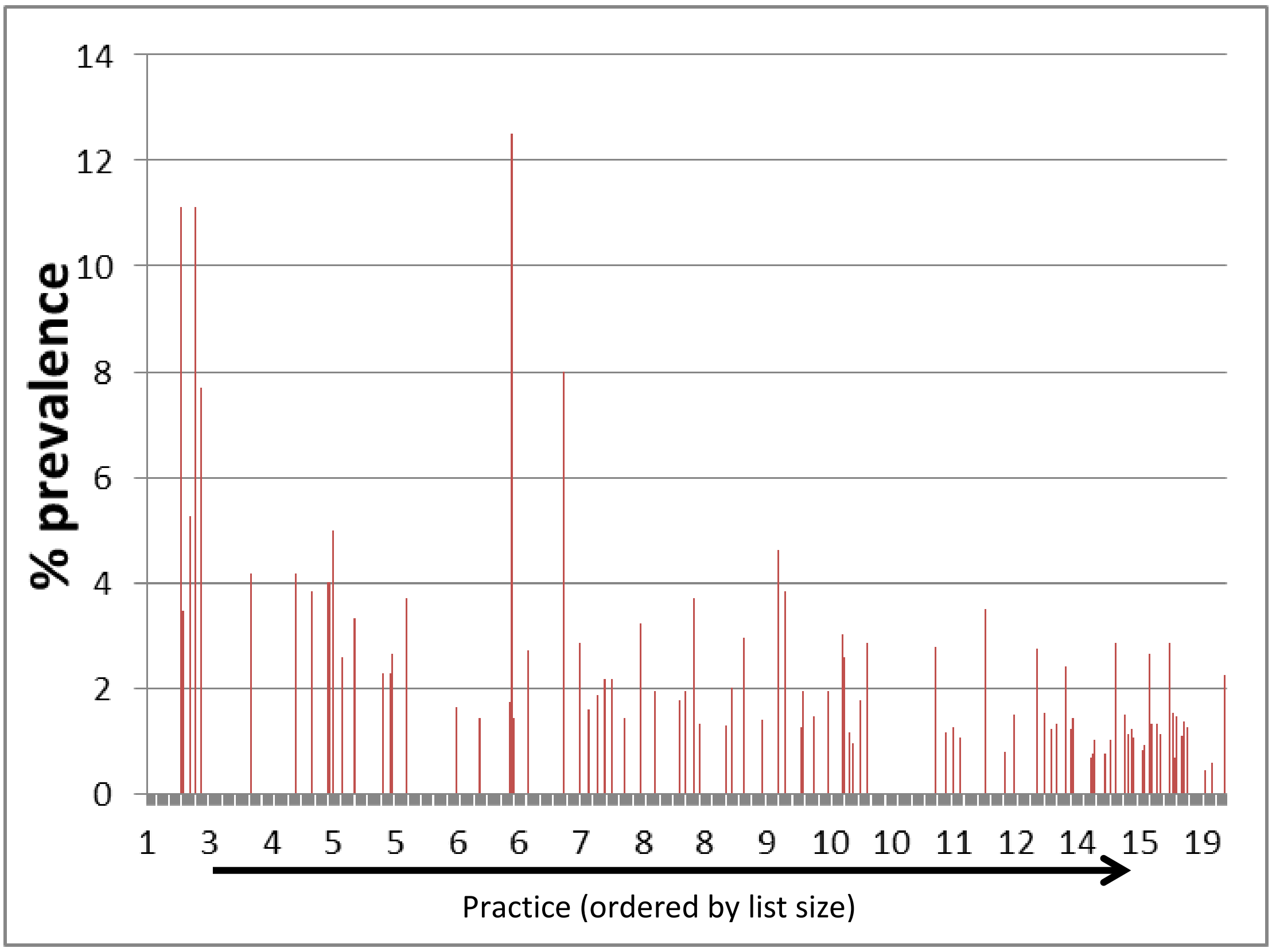

P6. Heart failure and prescribed NSAID

Denominator – group at risk	Numerator – increased risk identified
Has read code for heart failure dated at least 3 months before audit date	Prescribed NSAID within the 3 months leading up to the audit date

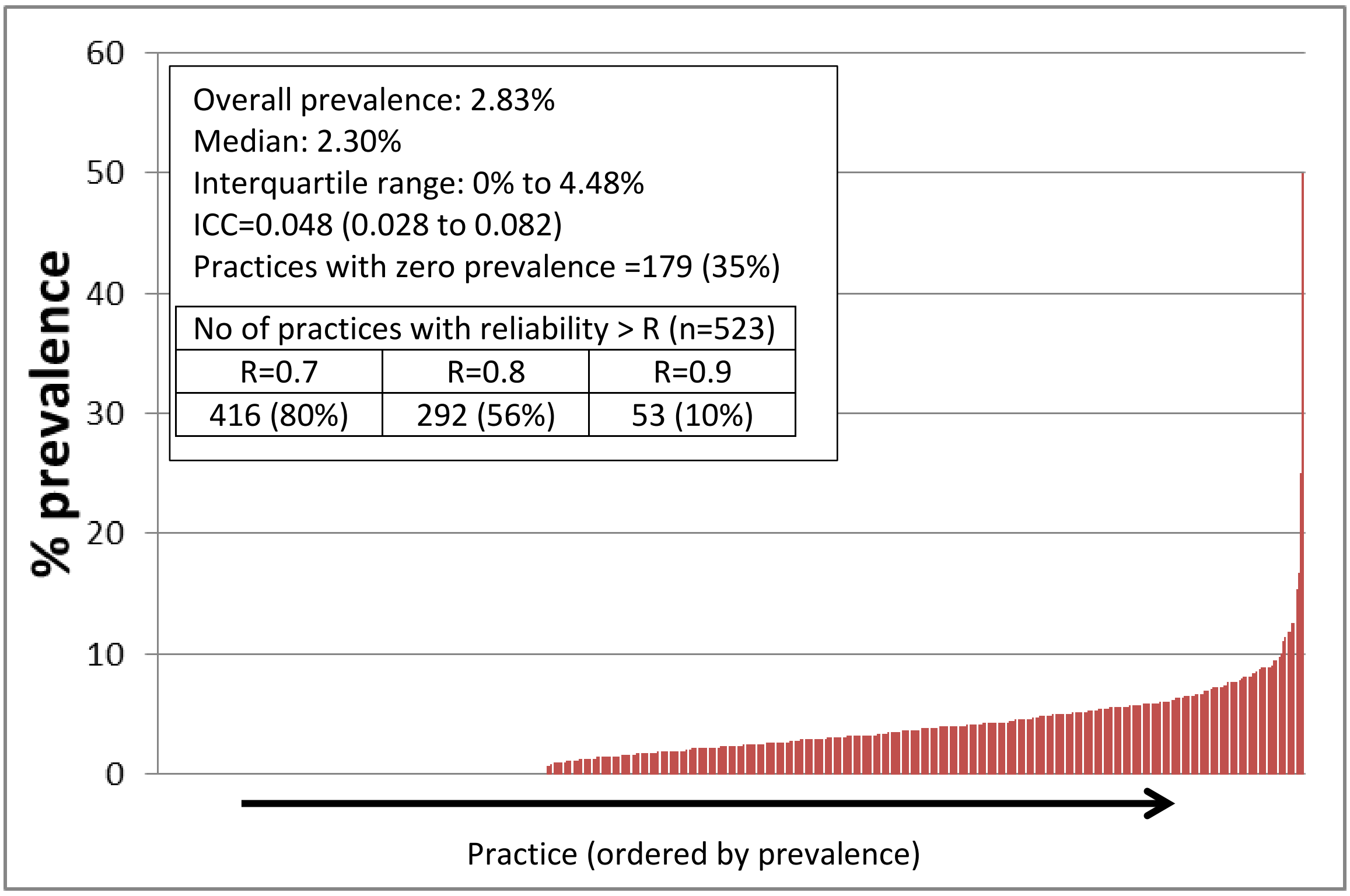

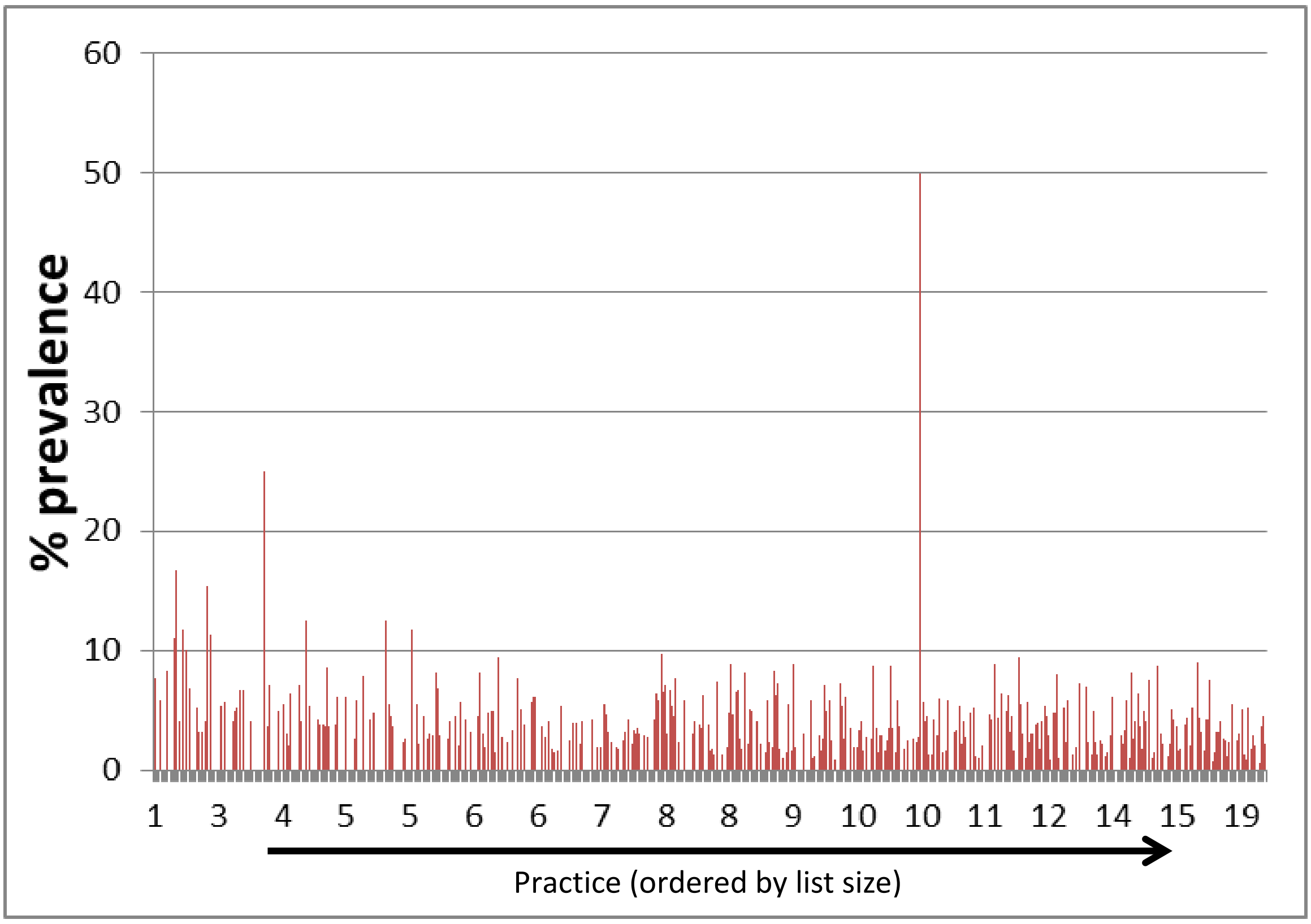

P7. CKD stage 3B, 4 or 5 and prescribed NSAID

Denominator – group at risk	Numerator – increased risk identified
Has read code for CKD stage 3B, 4 or 5 at least 3 months before audit date	Prescribed NSAID within the 3 months leading up to the audit date

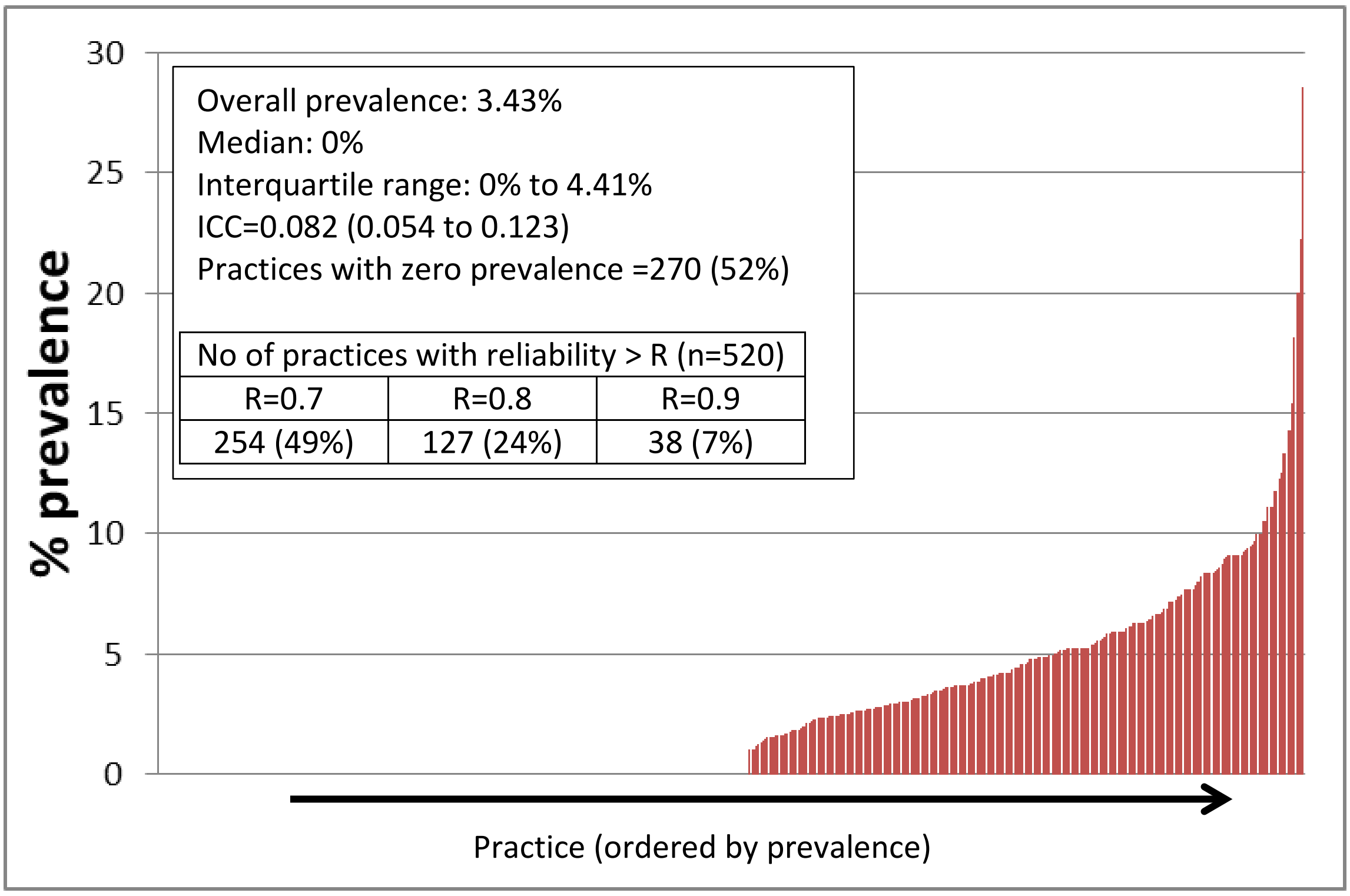

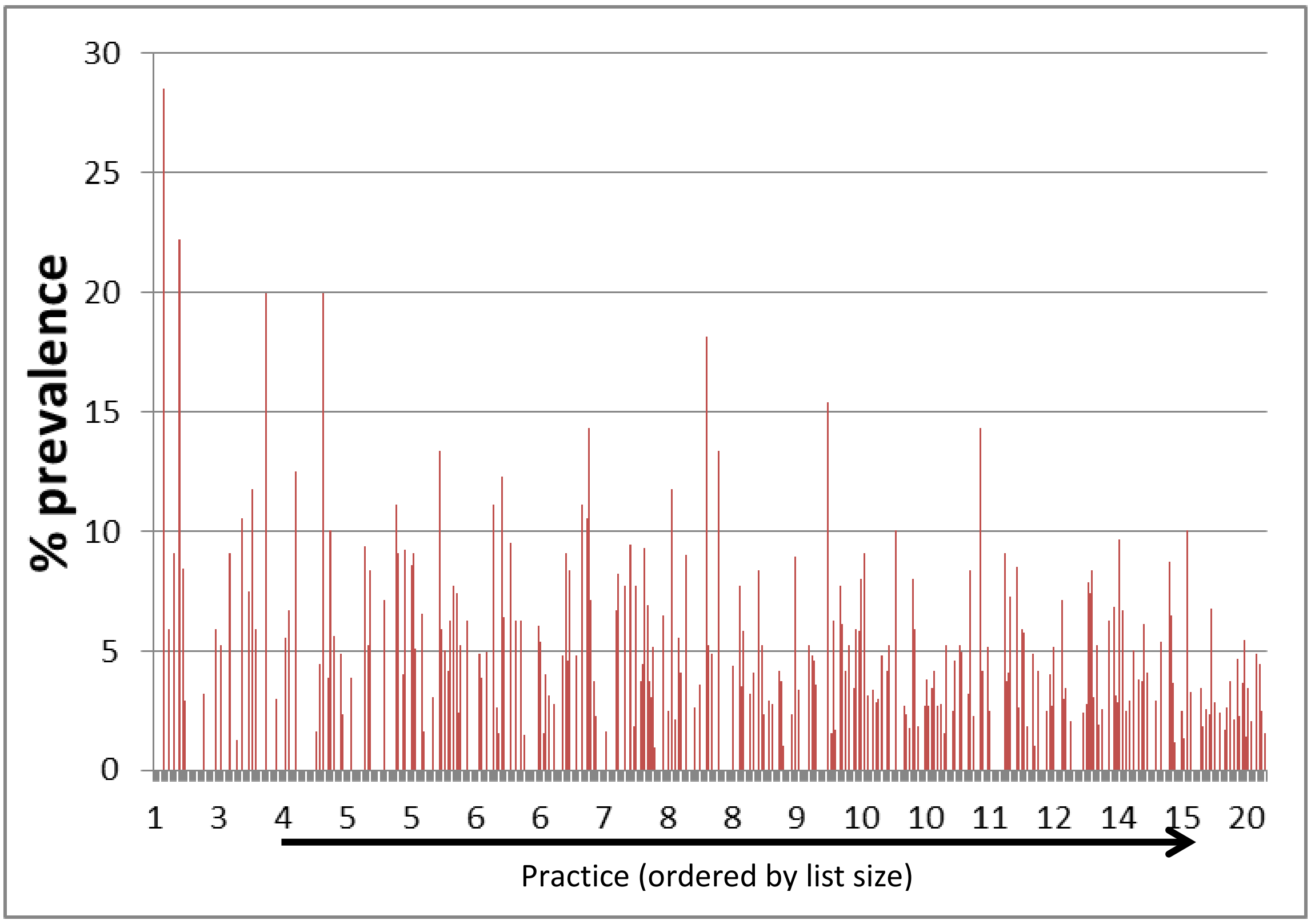

P8. CKD stage 4 or 5 and prescribed metformin

Denominator – group at risk	Numerator – increased risk identified
Has read code for CKD stage 4 or 5 at least 3 months before audit date	Prescribed metformin within the 3 months leading up to the audit date

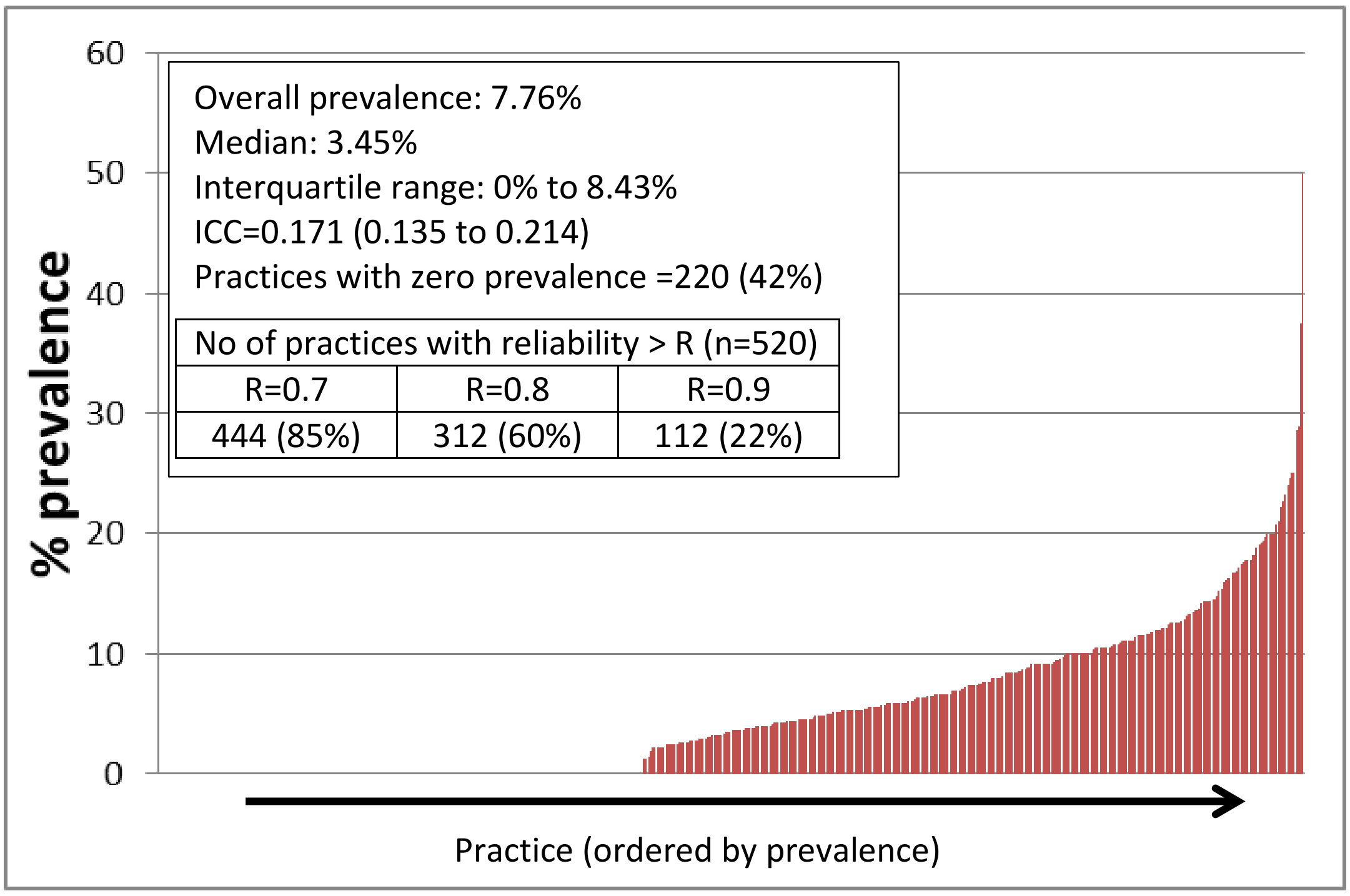

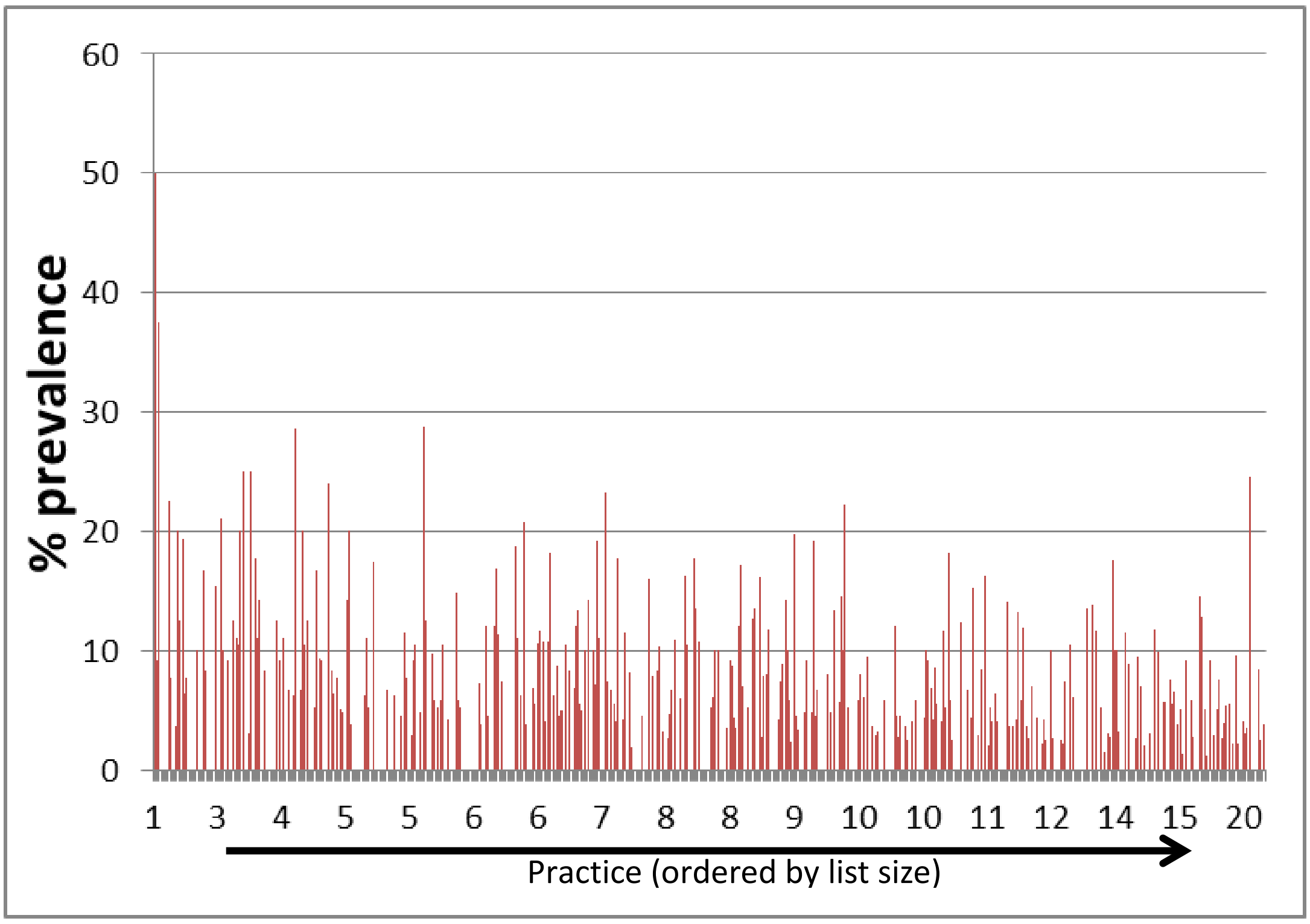

P9. CKD stage 3B, 4 or 5 and prescribed digoxin

Denominator – group at risk	Numerator – increased risk identified
Has read code for CKD stage 3B, 4 or 5 at least 3 months before audit date	Prescribed digoxin at a daily dose > 125 µg within the 3 months leading up to the audit date

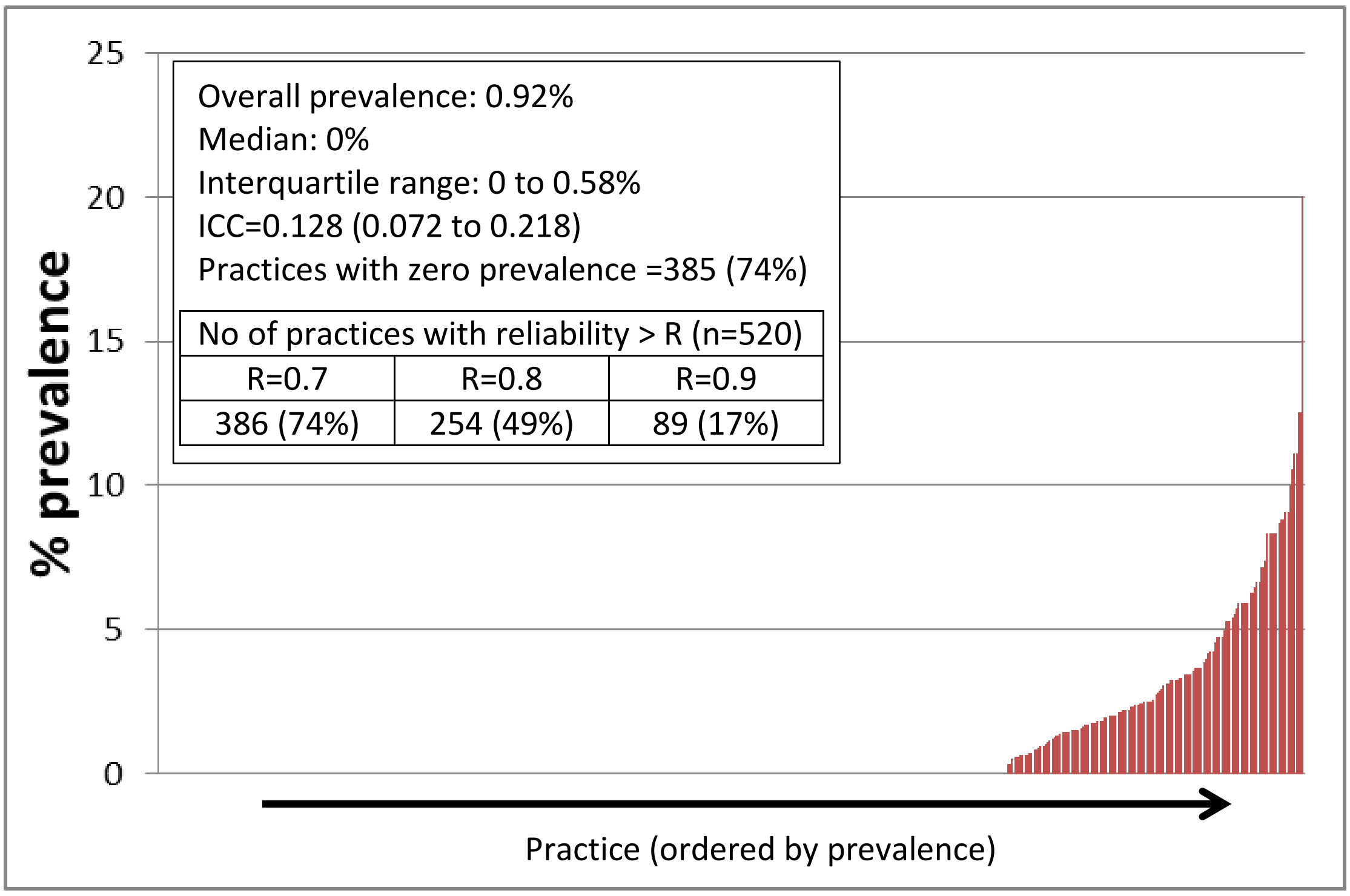

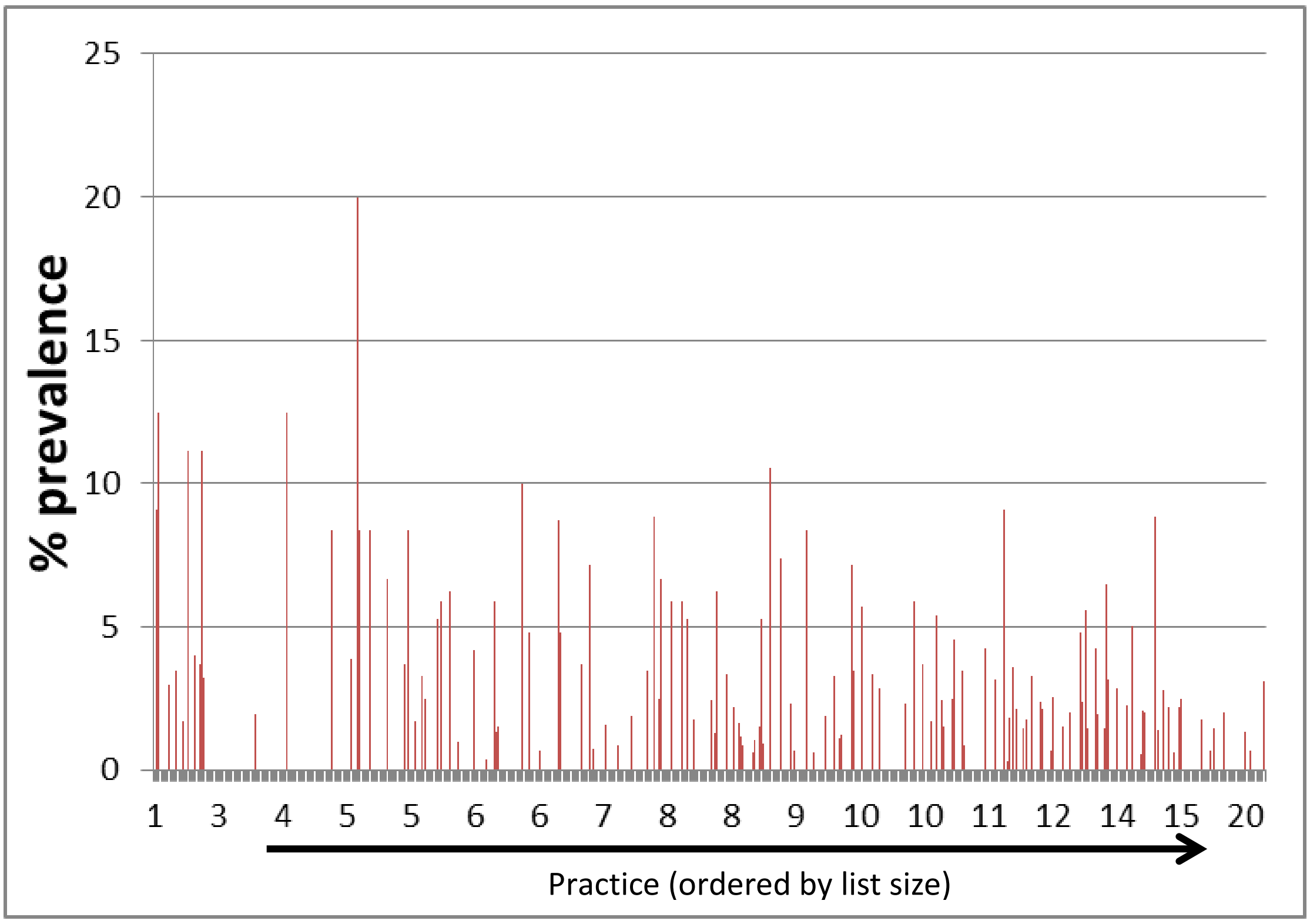

P10. Prescribed warfarin without gastroprotection & NSAID

Denominator – group at risk	Numerator – increased risk identified
Has a prescription for warfarin and no gastroprotection within the 6 months leading up to the audit date	Prescribed NSAID within the 6 months leading up to the audit date

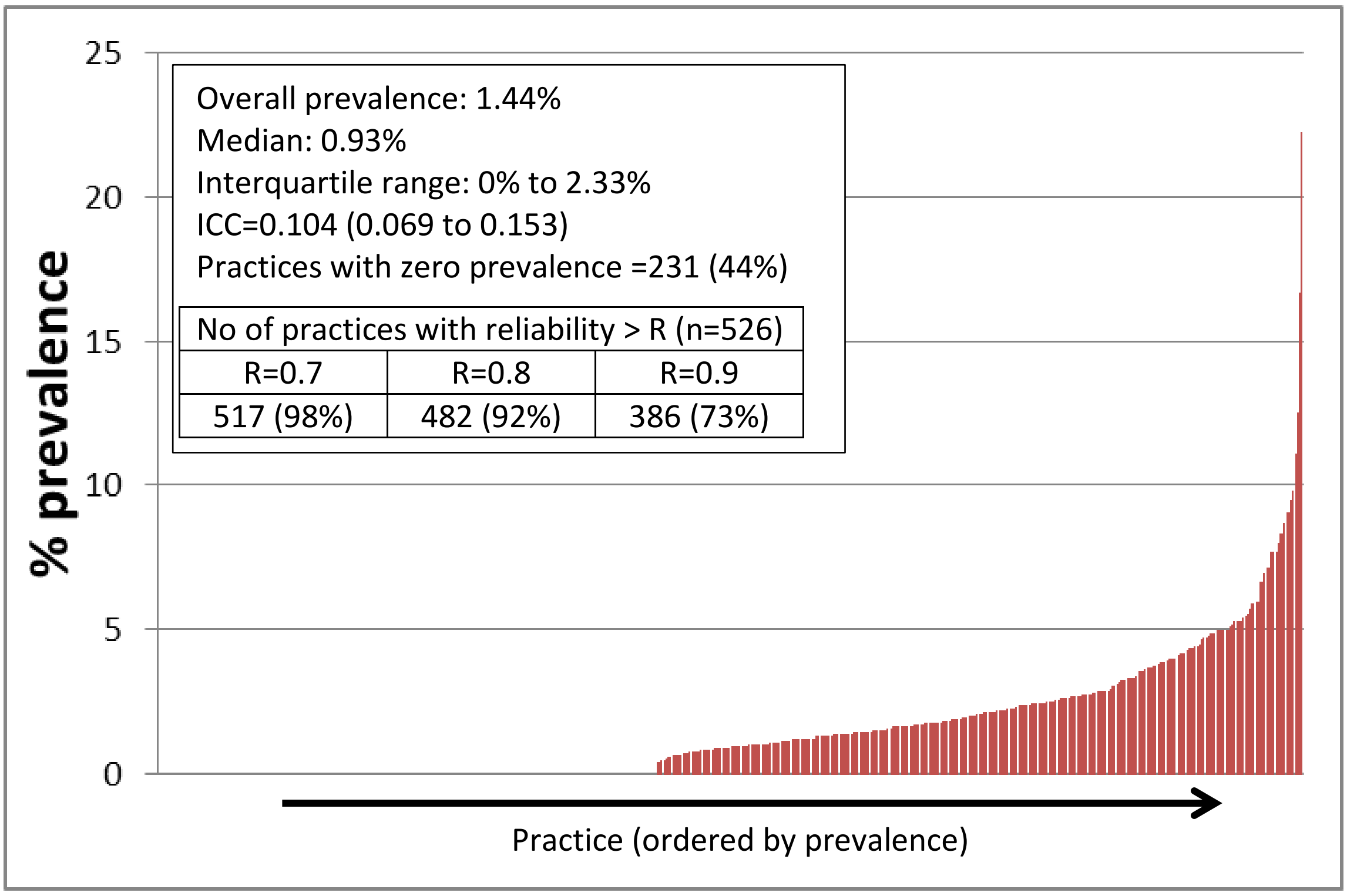

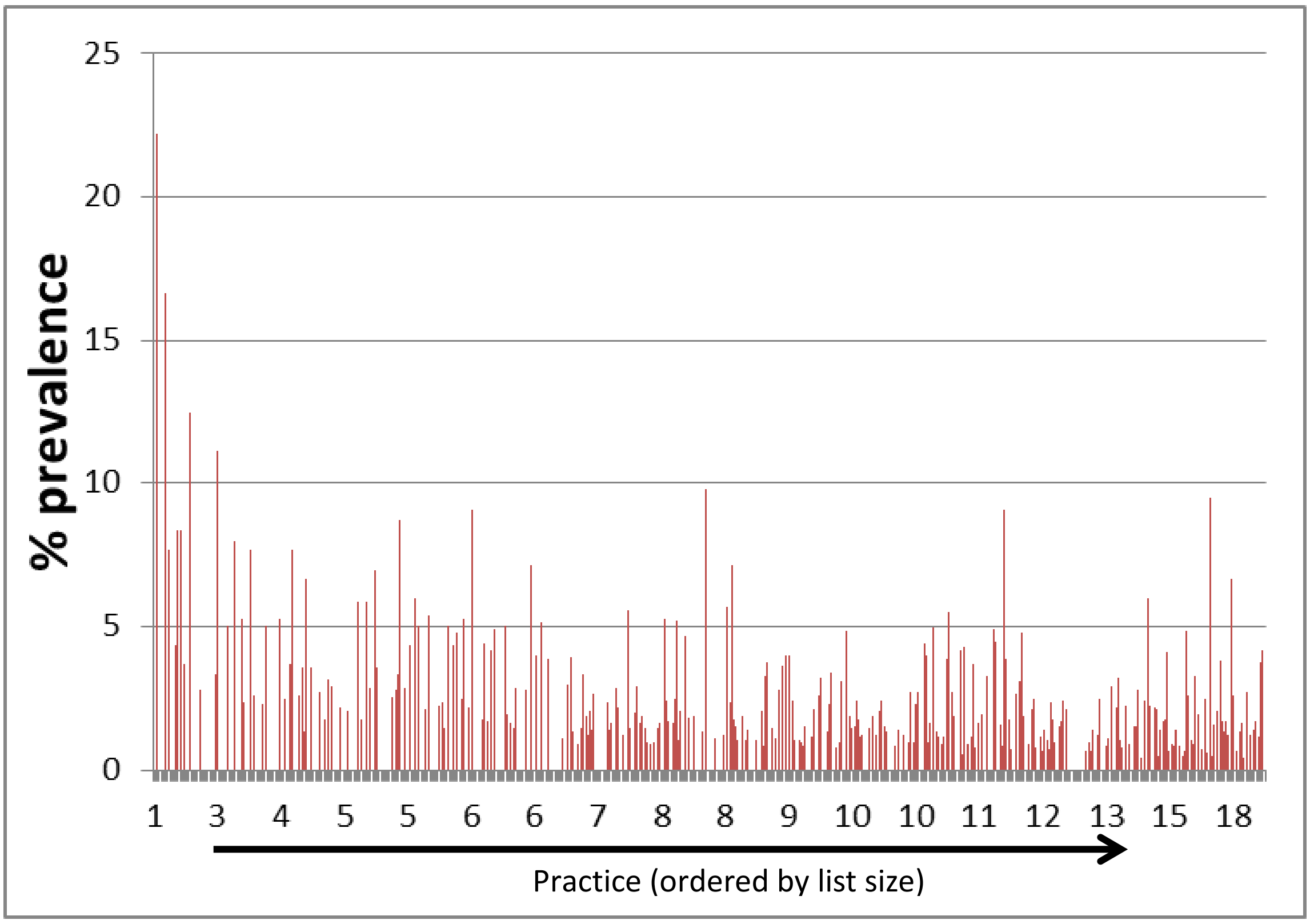

P11. Prescribed warfarin without gastroprotection & aspirin

Denominator – group at risk	Numerator – increased risk identified
Has a prescription for warfarin and no gastroprotection within the 6 months leading up to the audit date	Prescribed aspirin within the 6 months leading up to the audit date

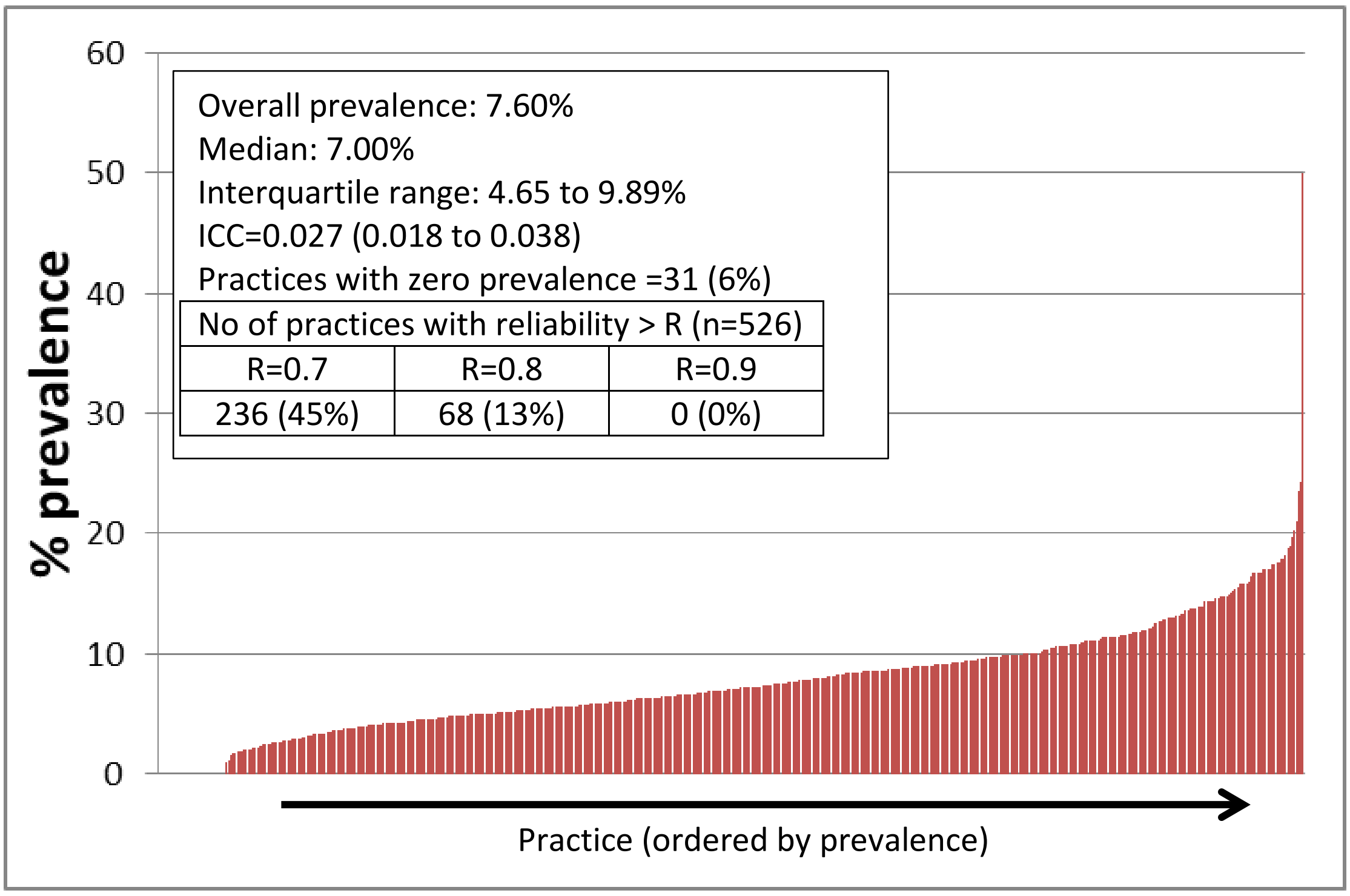

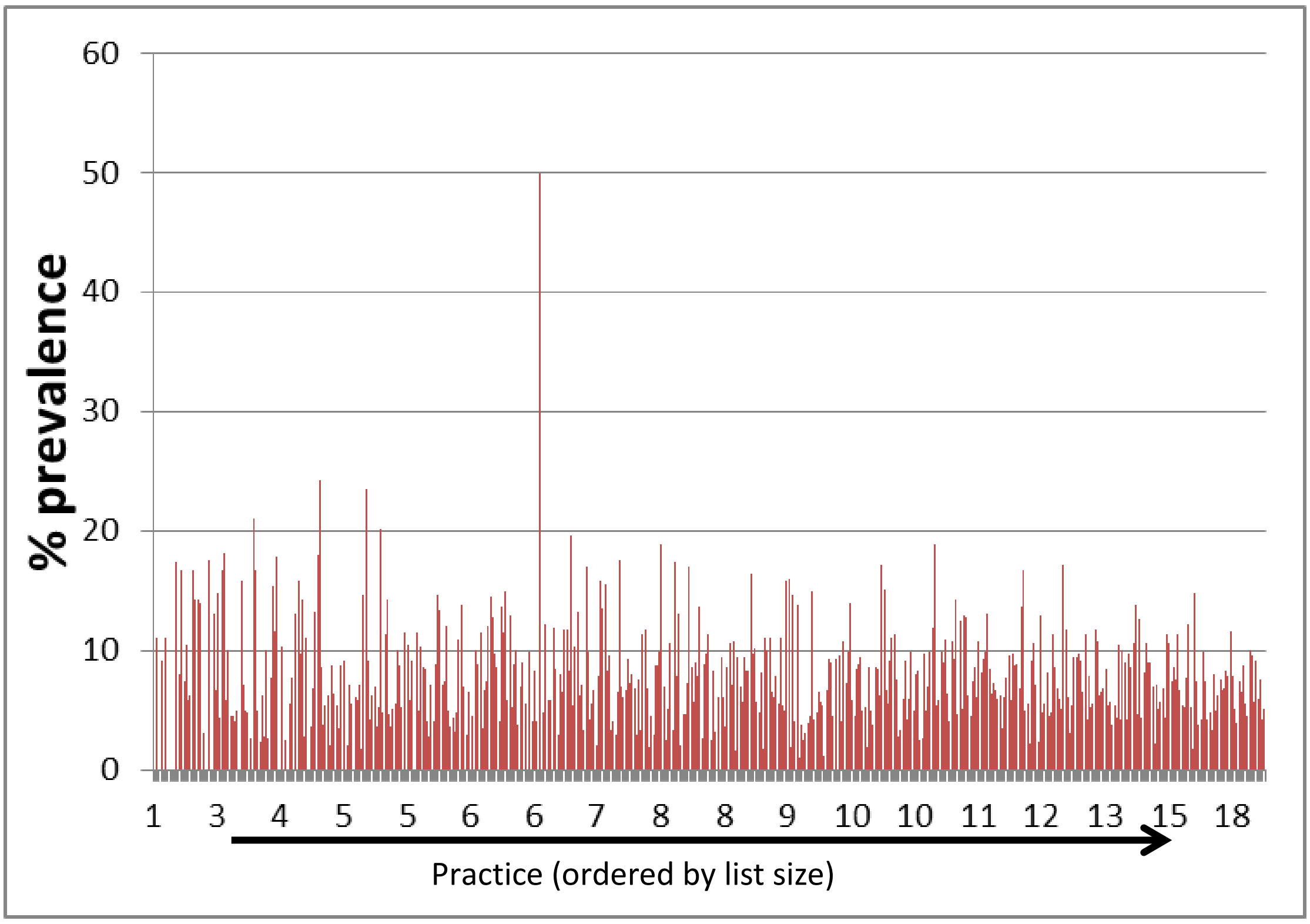

P12. Aged 65 or over, dementia & no psychosis & prescribed antipsychotics

Denominator – group at risk	Numerator – increased risk identified
Aged 65 or over on the audit date and have a read code for dementia and no read code for psychosis at least 6 months before audit date	Prescribed antipsychotics at least twice within the 6 months leading up to the audit date

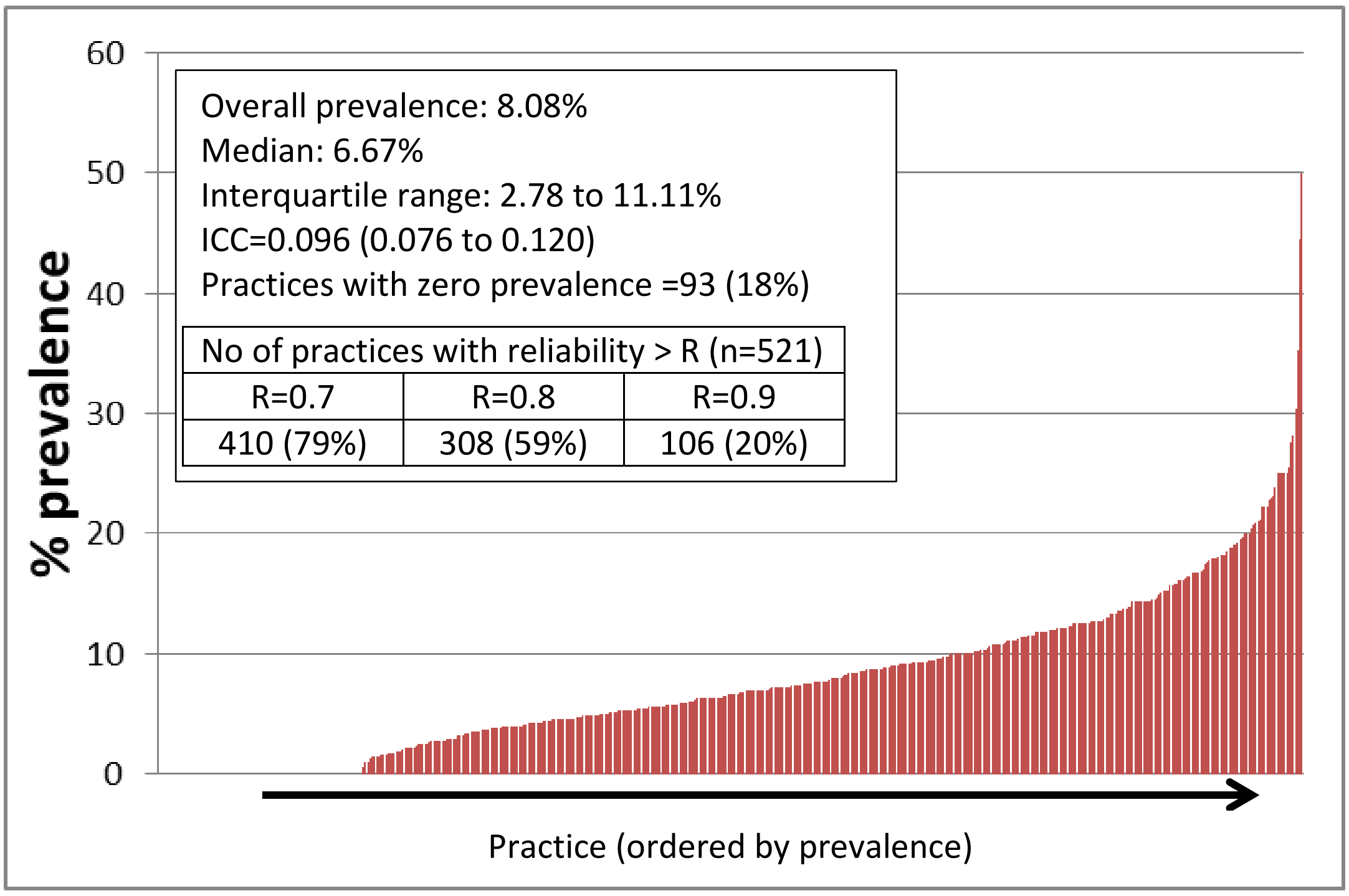

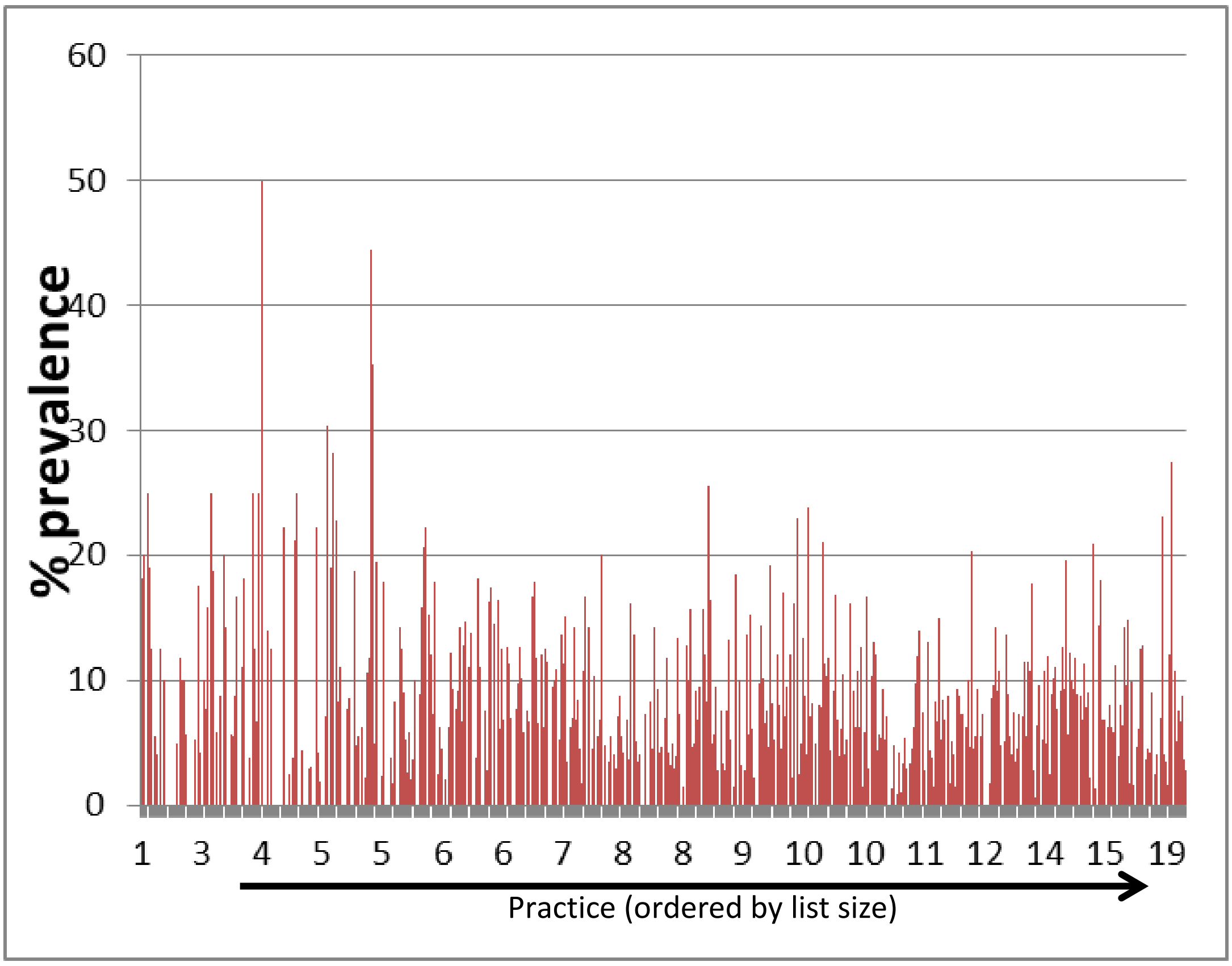

P13. Aged 65 or over, no gastroprotection and prescribed NSAID

Denominator – group at risk	Numerator – increased risk identified
Aged 65 or over on the audit date and not prescribed PPI, misoprostol or H2 antagonist within the 6 months leading up to the audit date	Prescribed NSAID within the 6 months leading up to the audit date

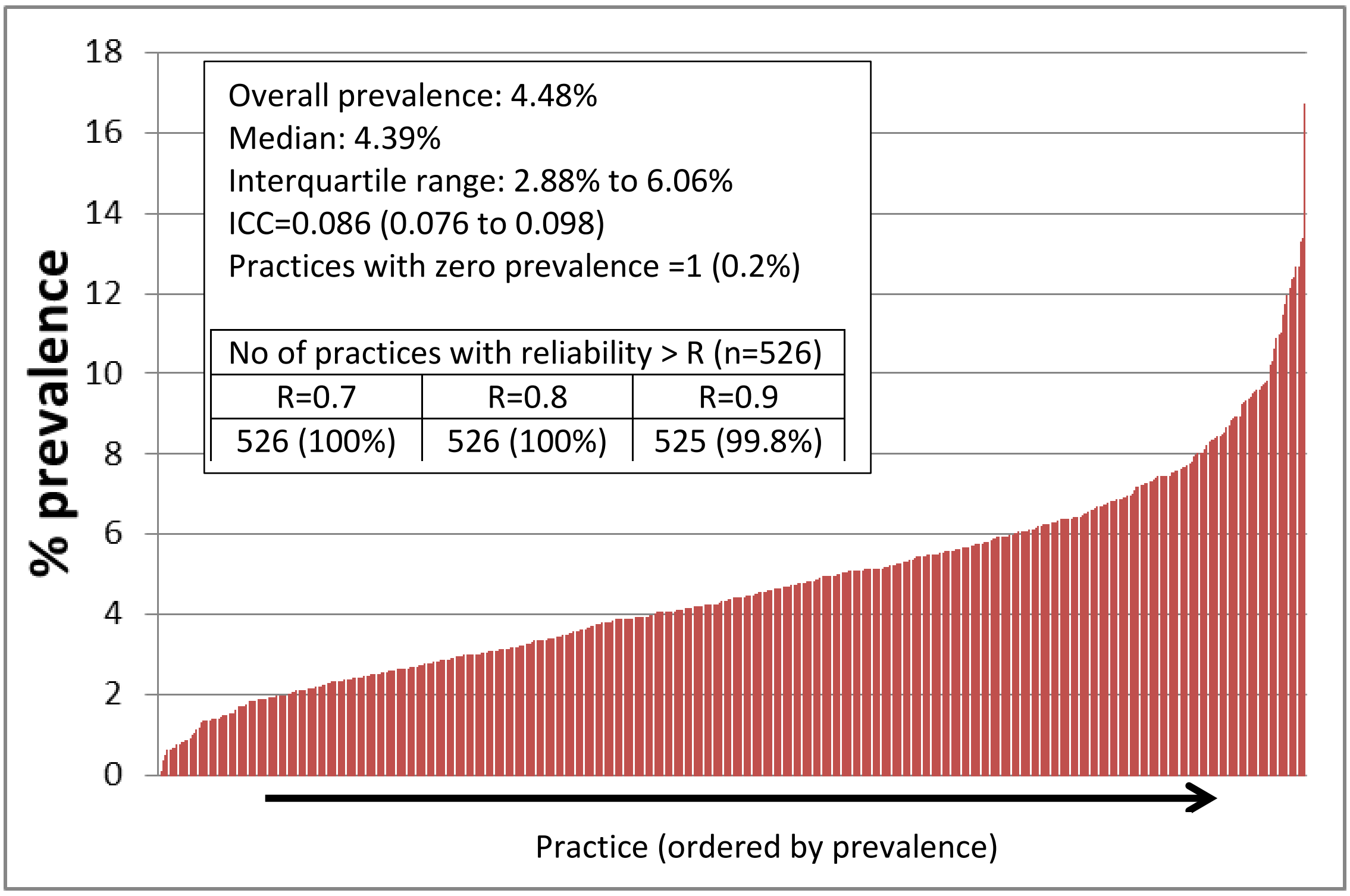

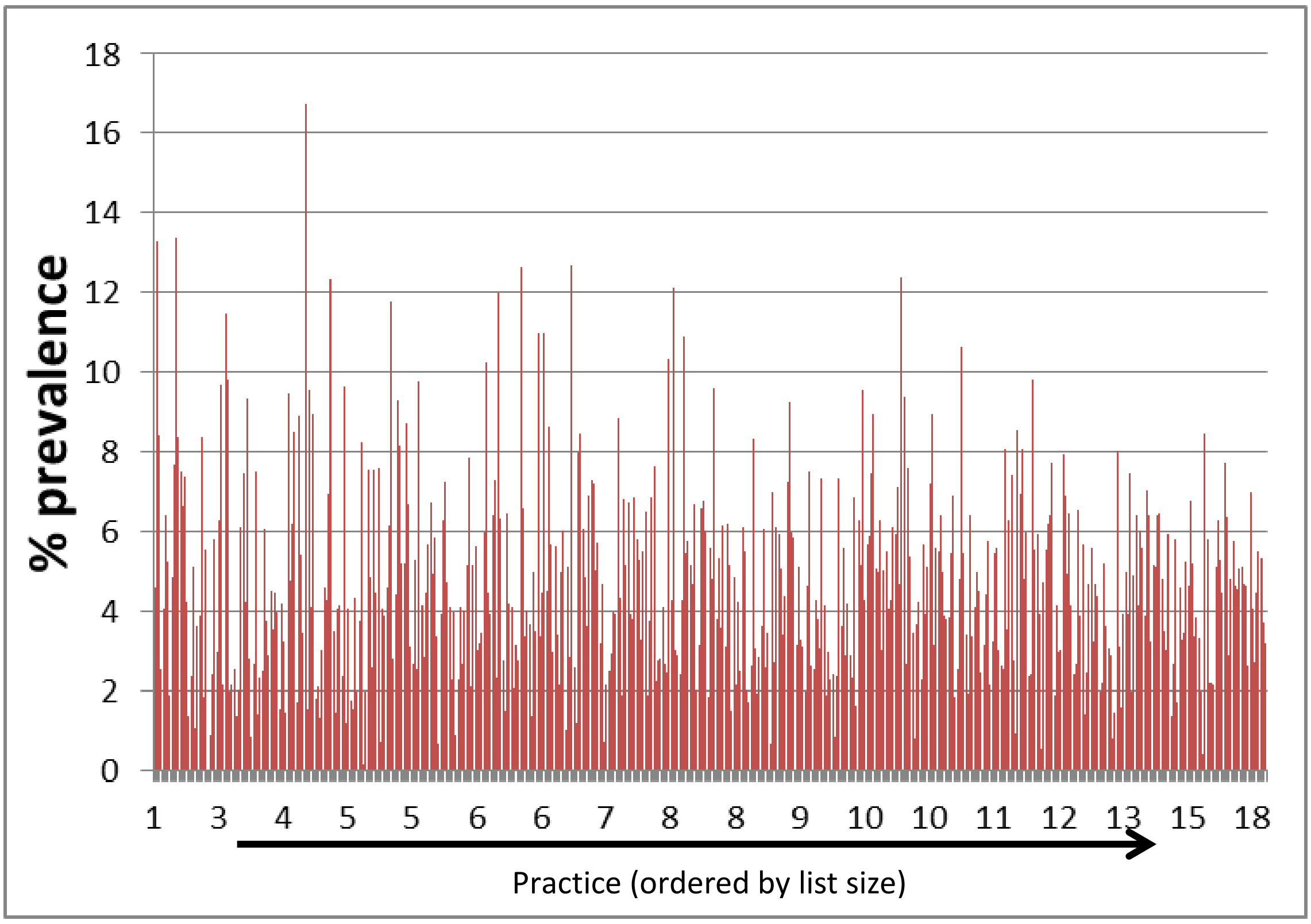

P14. CKD stage 3B, 4 or 5 and prescribed ACEI & NSAID

Denominator – group at risk	Numerator – increased risk identified
Diagnosis of CKD stage 3B, 4 or 5 and prescribed an ACEI	Prescribed NSAID within the 3 months leading up to the audit date

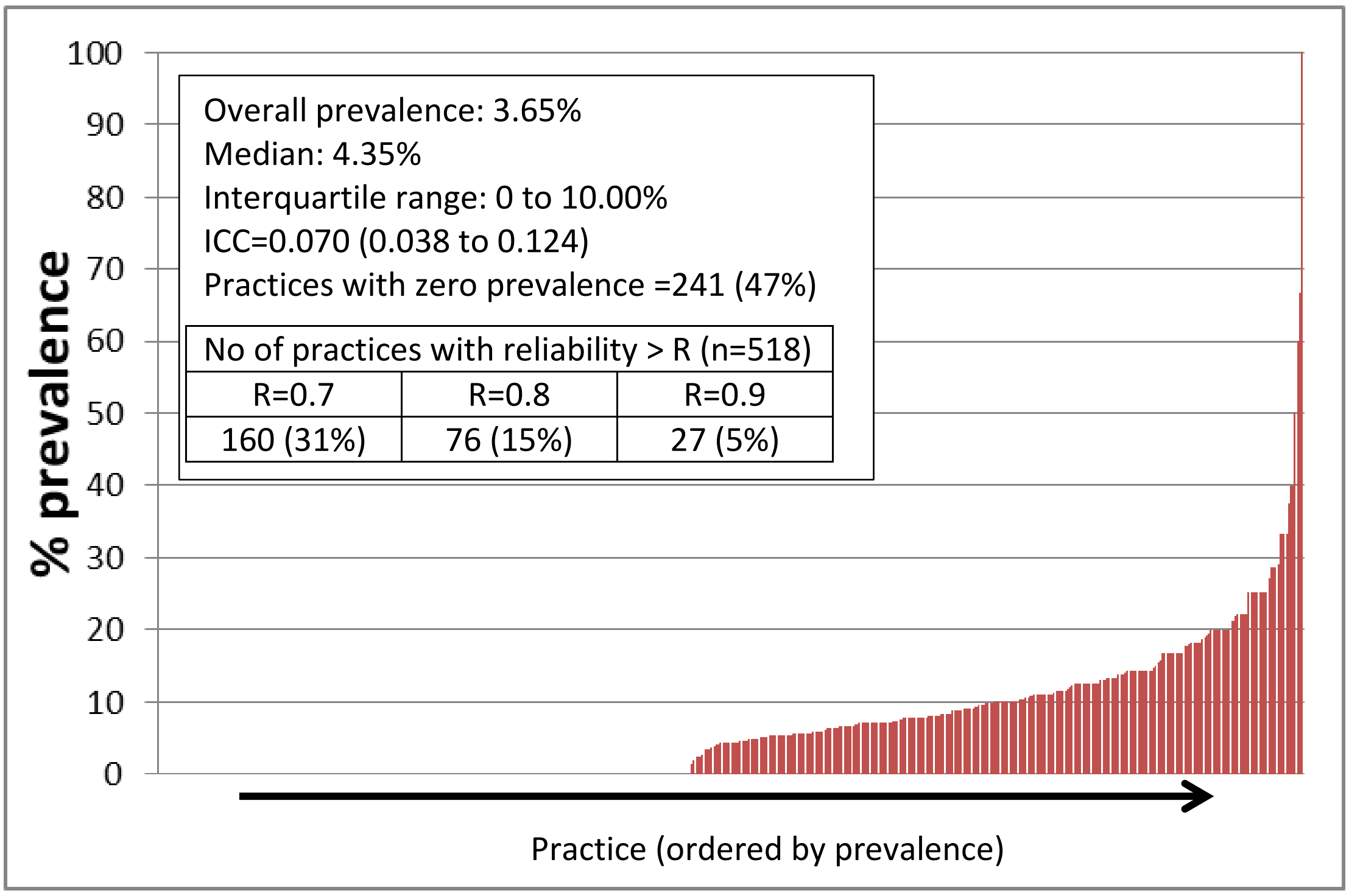

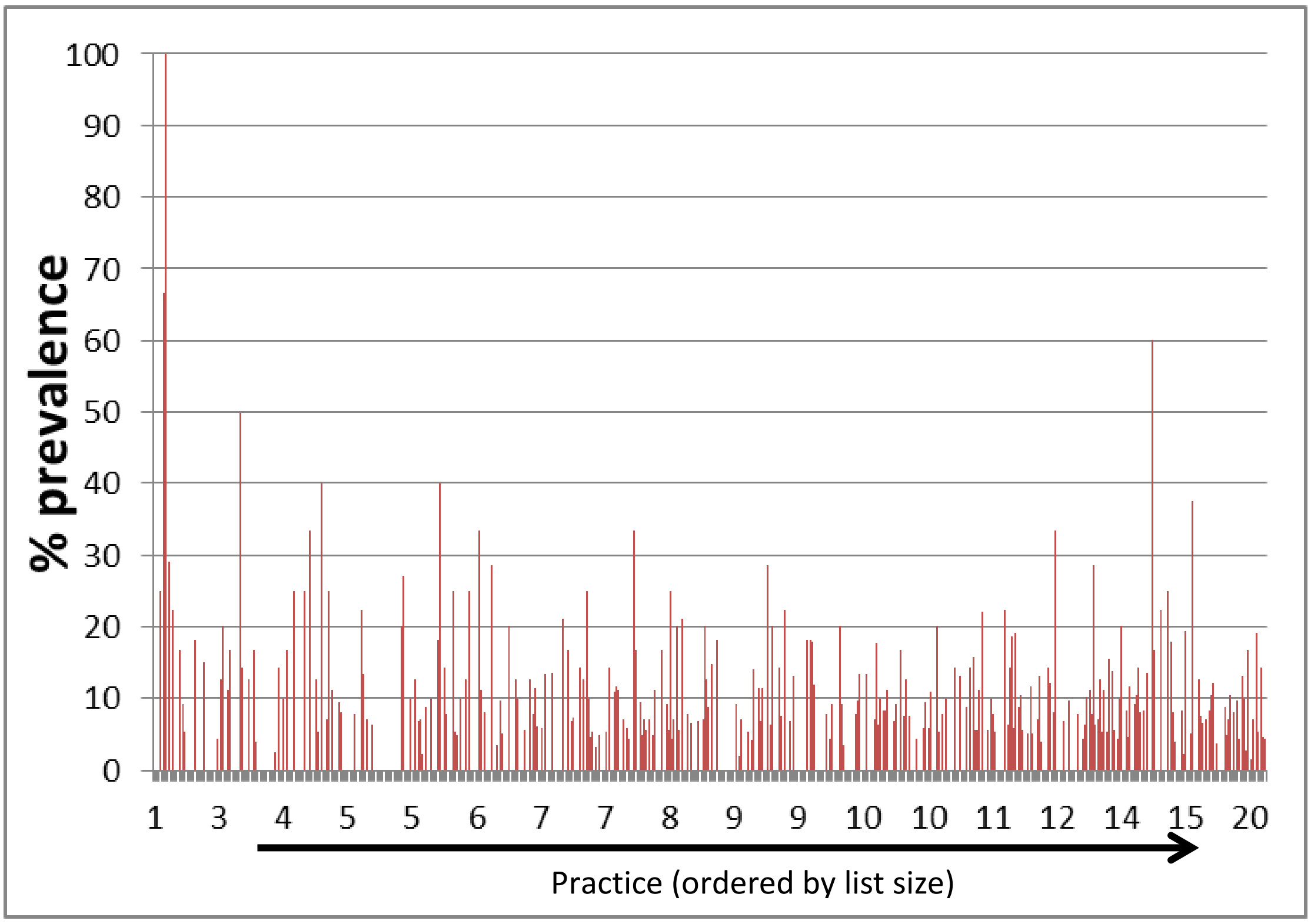

P15. CKD stage 3B, 4 or 5 and prescribed ACEI & loop diuretic & NSAID

Denominator – group at risk	Numerator – increased risk identified
Diagnosis of CKD stage 3B, 4 or 5 and prescribed an ACEI and a loop diuretic	Prescribed NSAID within the 3 months leading up to the audit date

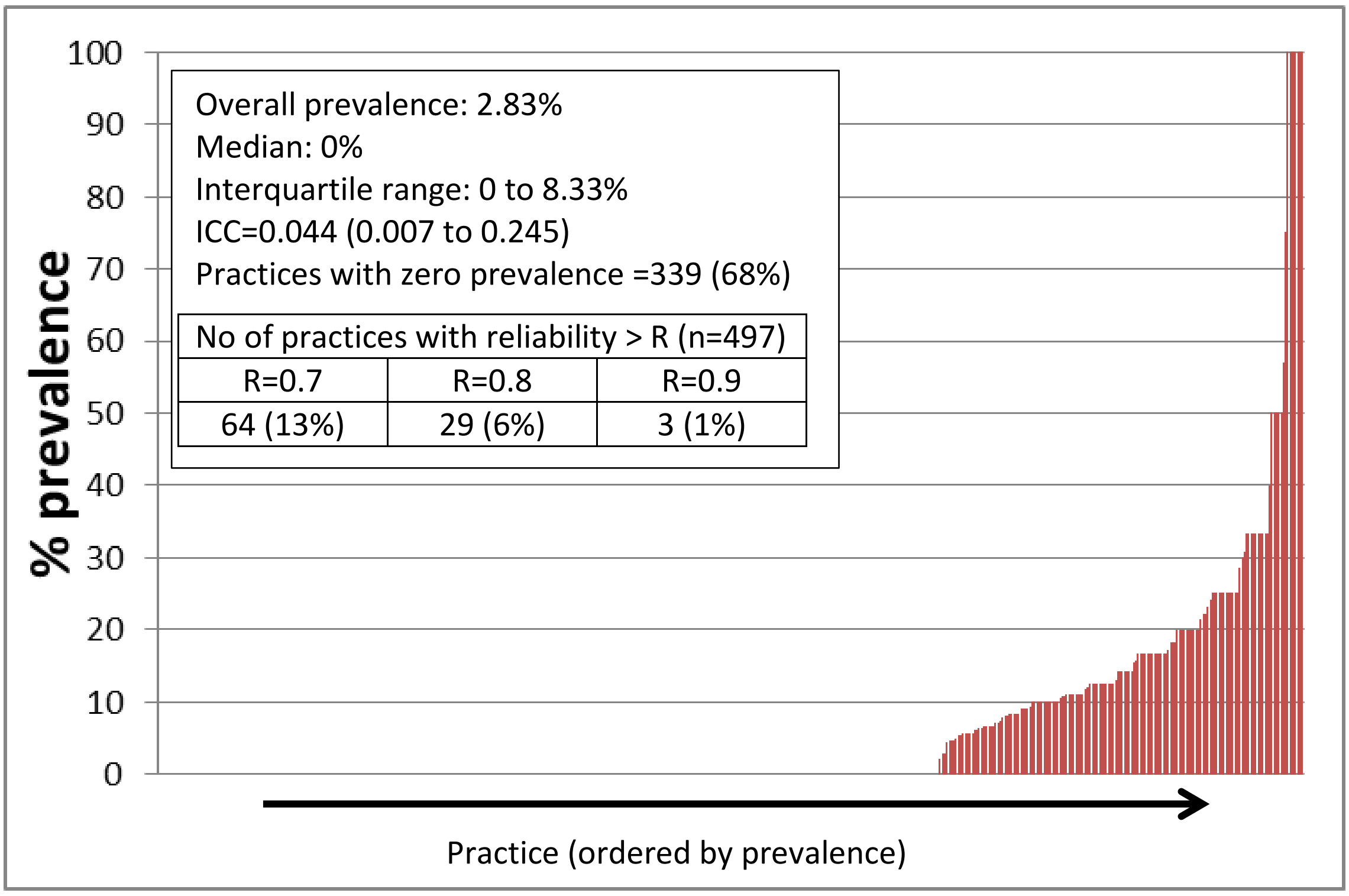

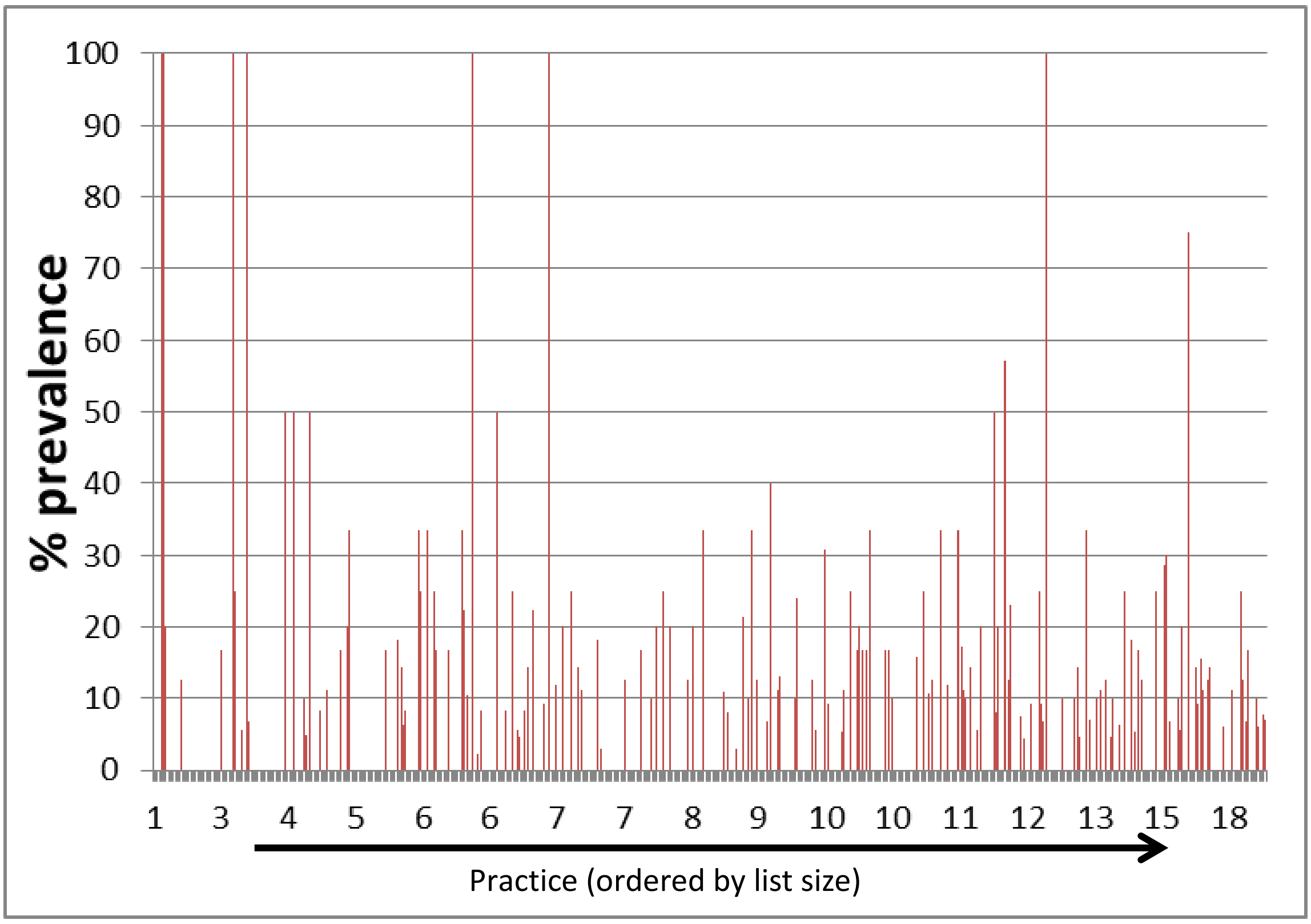

P16. Female, history of thrombosis & prescribed CHC

Denominator – group at risk	Numerator – increased risk identified
Female and has read code for venous or arterial thrombosis at least 6 months before audit date	Prescribed CHC within the 6 months leading up to the audit date

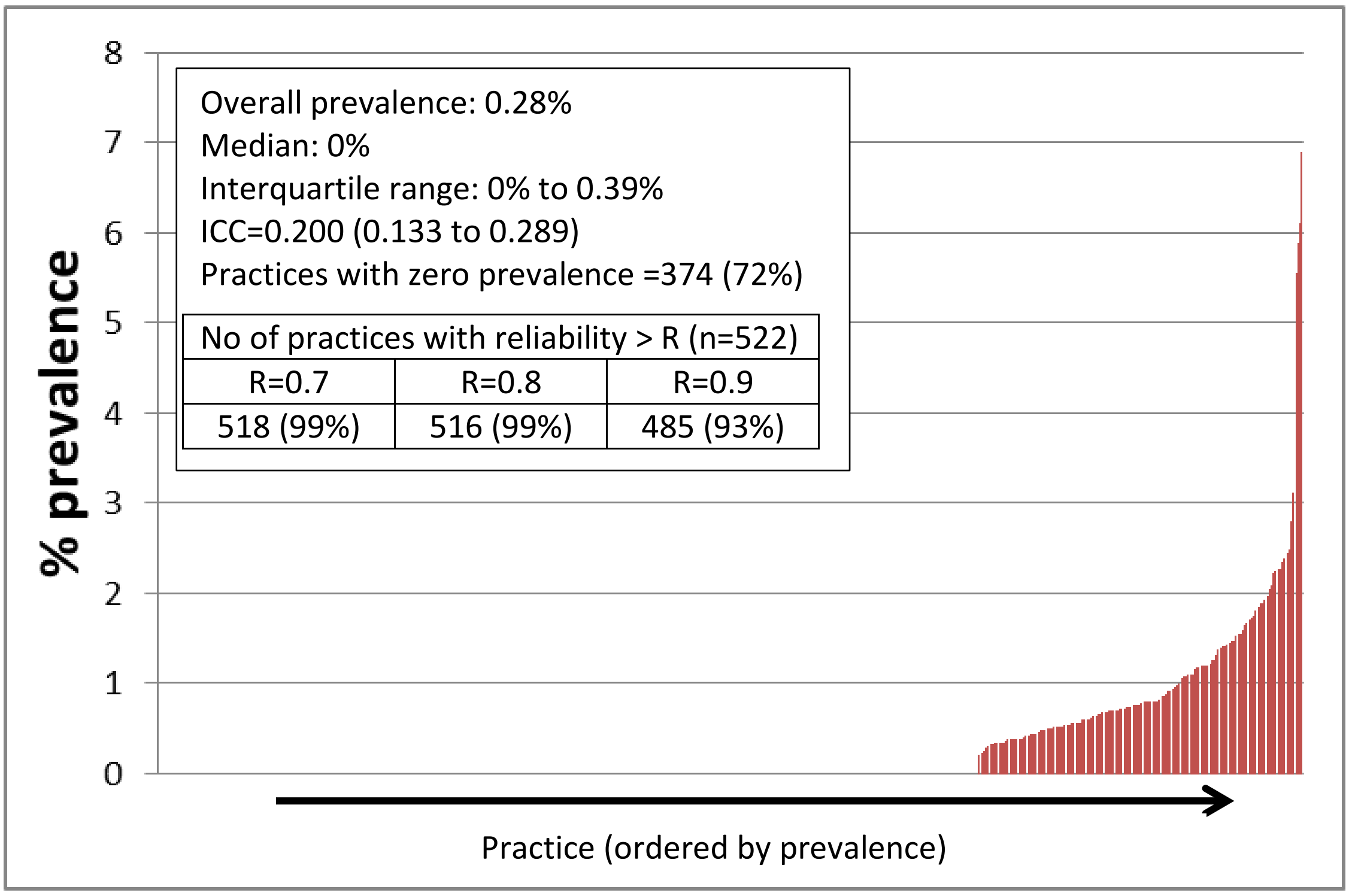

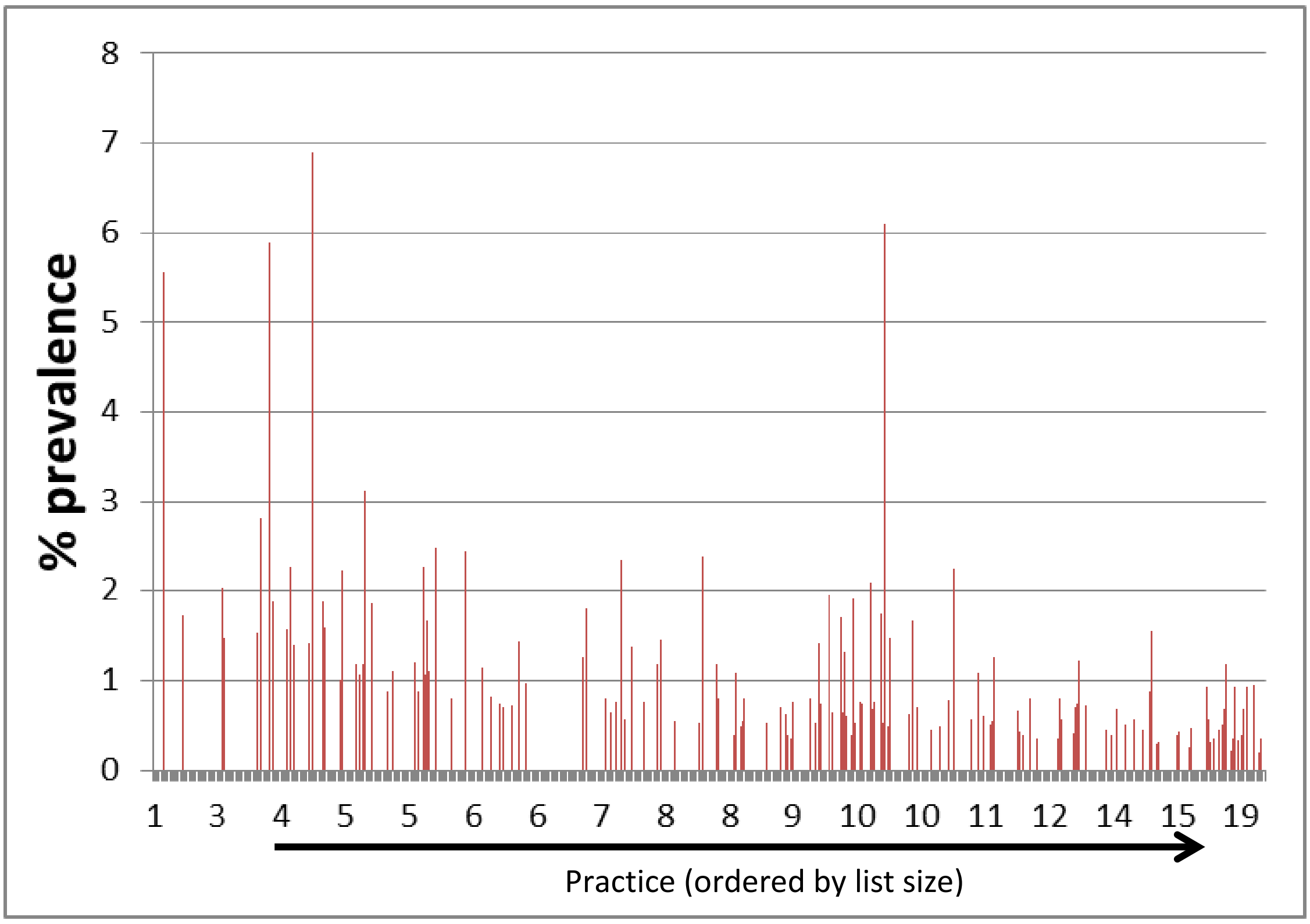

P17. Female, BMI>40 & prescribed CHC

Denominator – group at risk	Numerator – increased risk identified
Female and read code for BMI>40 between 66 and 6 months prior to the audit date and without a more recent read code for BMI<40	Prescribed CHC within the 6 months leading up to the audit date

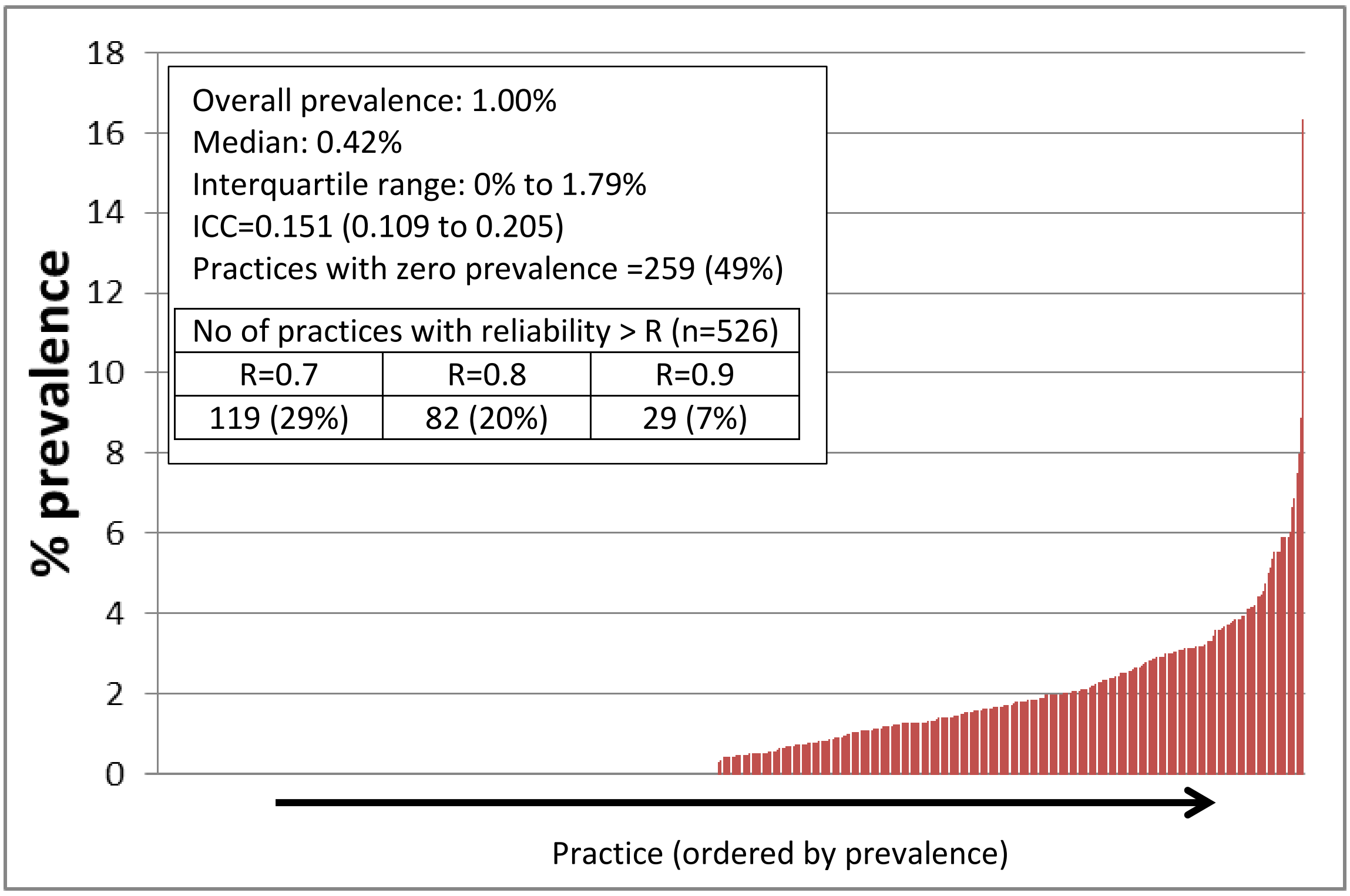

Overall prevalence: 1.00%
Median: 0.42%
Interquartile range: 0% to 1.79%
ICC=0.151 (0.109 to 0.205)
Practices with zero prevalence =259 (49%)

No of practices with reliability > R (n=526)		
R=0.7	R=0.8	R=0.9
119 (29%)	82 (20%)	29 (7%)

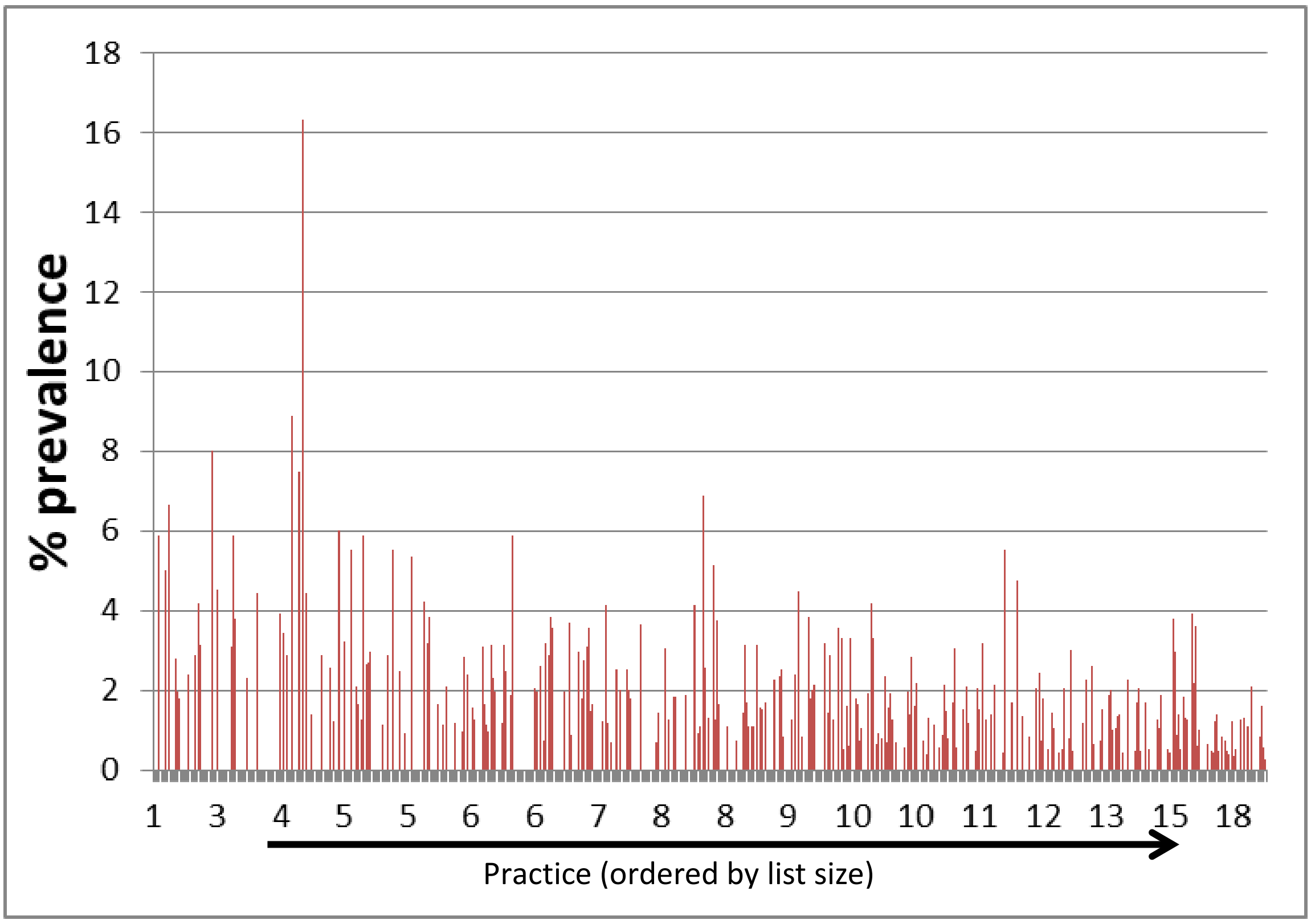

P18. Female, smoker & age>35 & prescribed CHC

Denominator – group at risk	Numerator – increased risk identified
Female, aged over 35 and most recent read code for smoking status is current smoker at least 6 months before audit date	Prescribed CHC within the 6 months leading up to the audit date

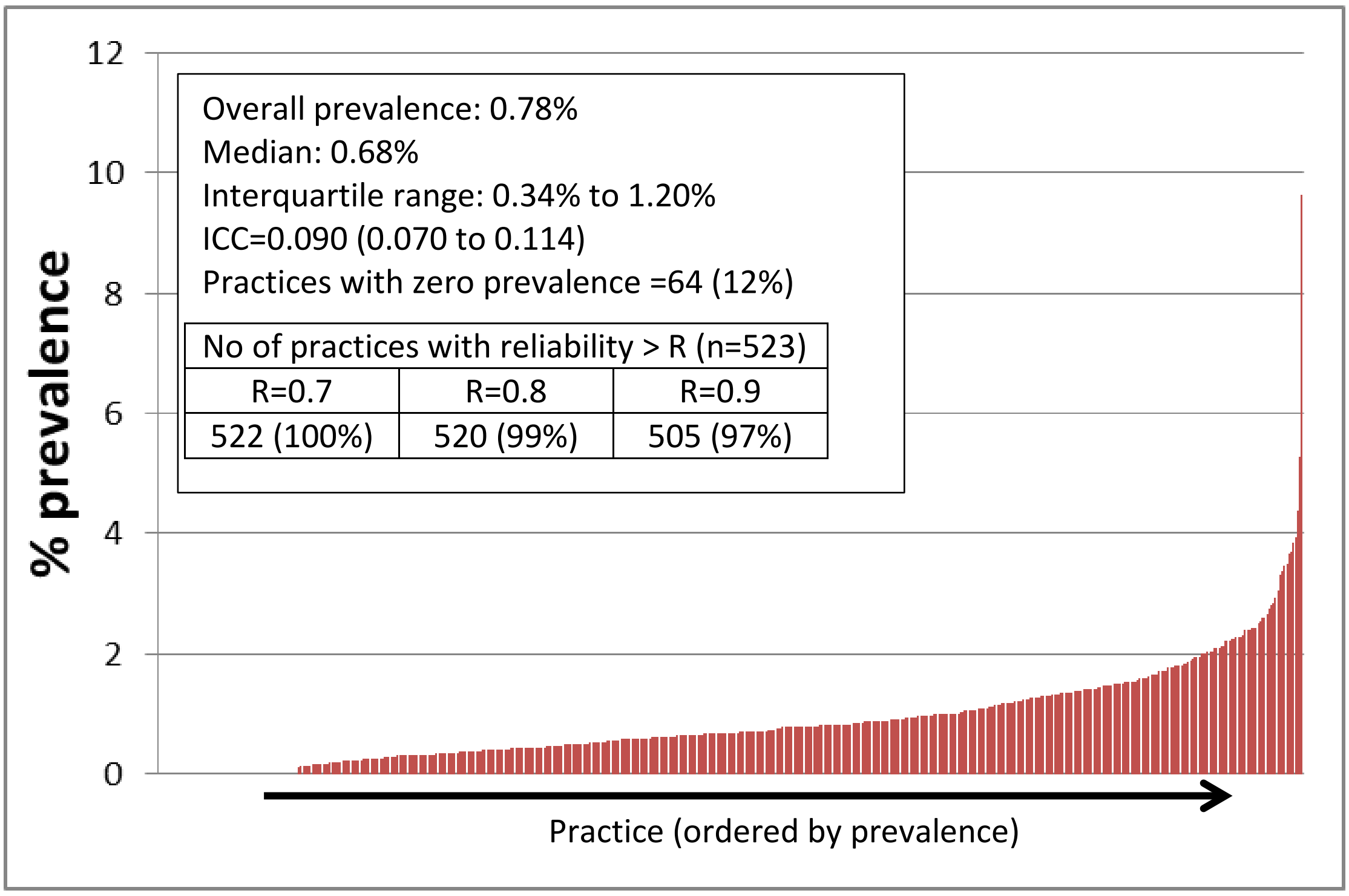

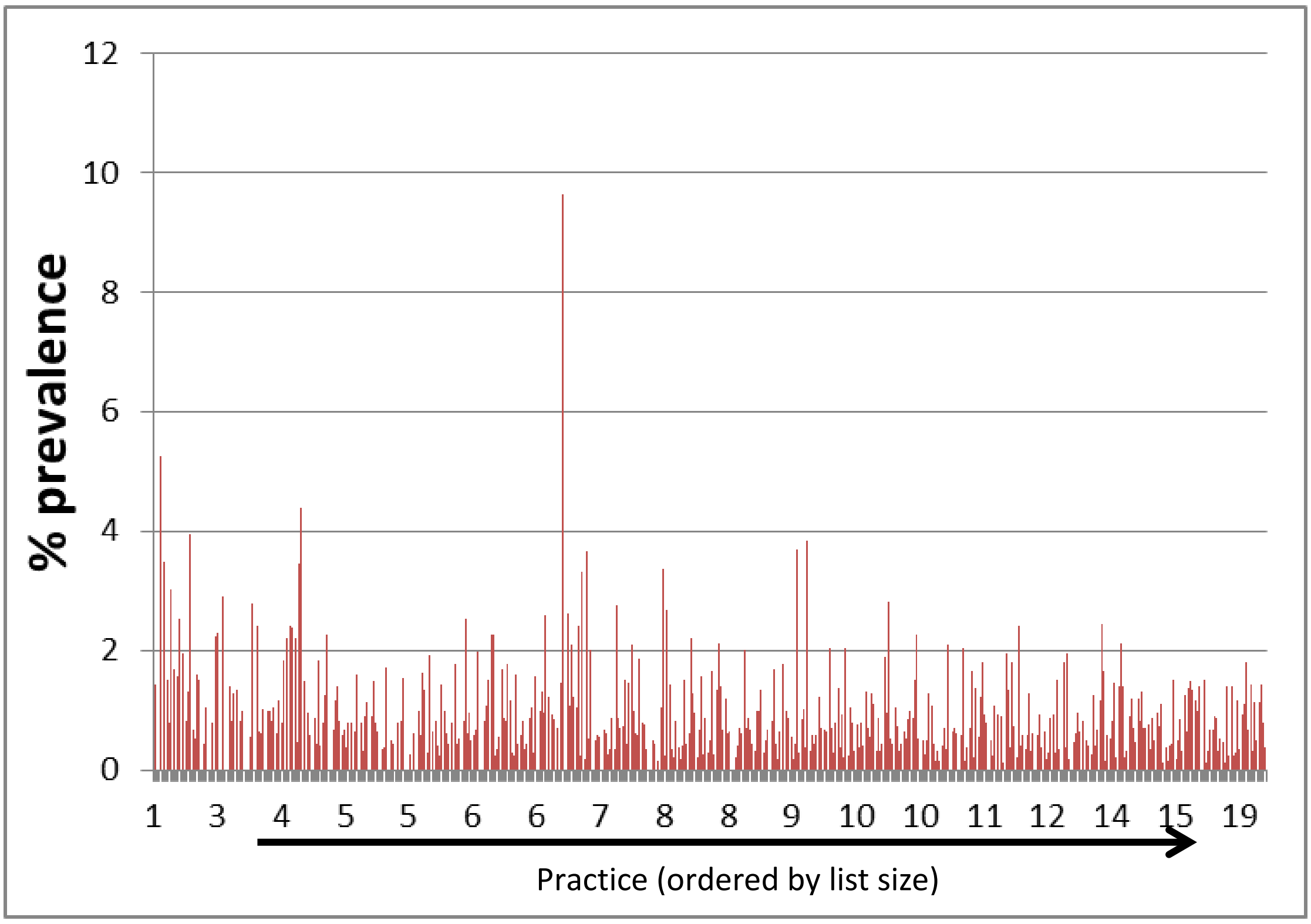

P19. Female, history of breast cancer & prescribed CHC

Denominator – group at risk	Numerator – increased risk identified
Female and has read code for breast cancer at least 6 months before audit date	Prescribed CHC or HRT within the 6 months leading up to the audit date

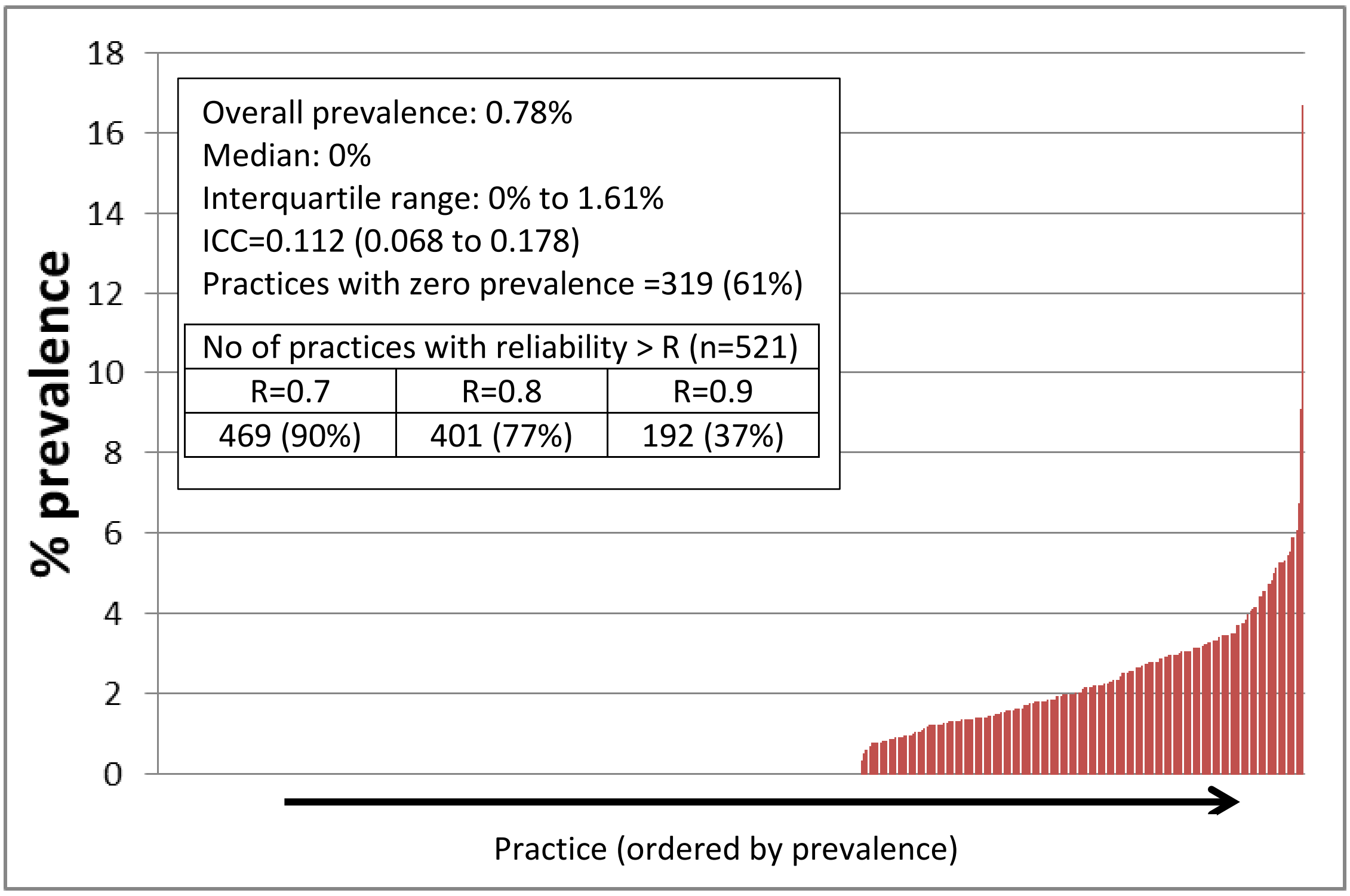

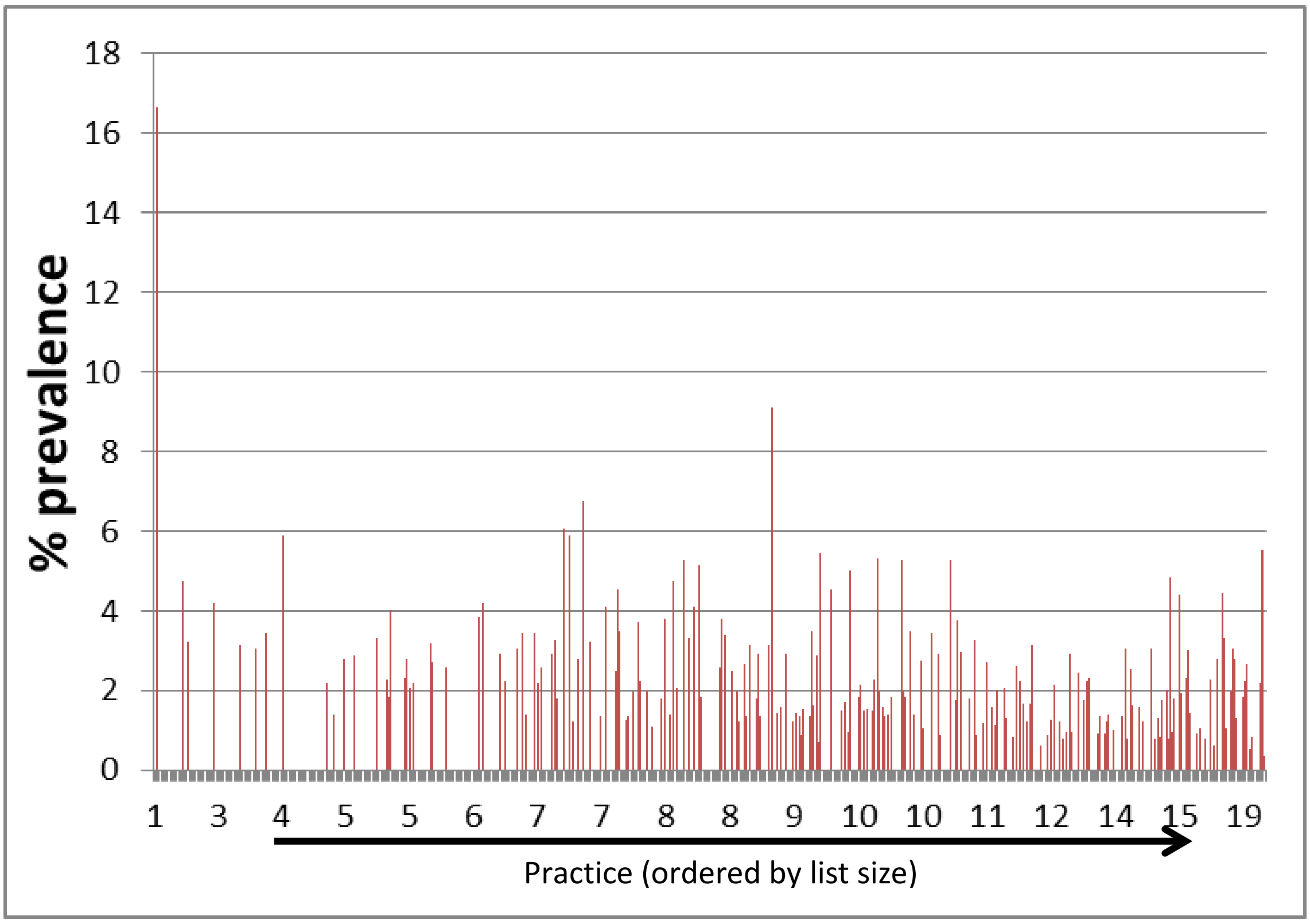

M1. Aged over 75 & prescribed repeat ACEI without U+E

Denominator – group at risk	Numerator – increased risk identified
Aged 75 or over and prescribed an angiotensin converting enzyme inhibitor (ACEI) or a loop diuretic at least 15 months before audit date and again within the 6 months leading up to the audit date	Have NOT had a renal function test nor an electrolytes test in the 15 months leading up to the audit date

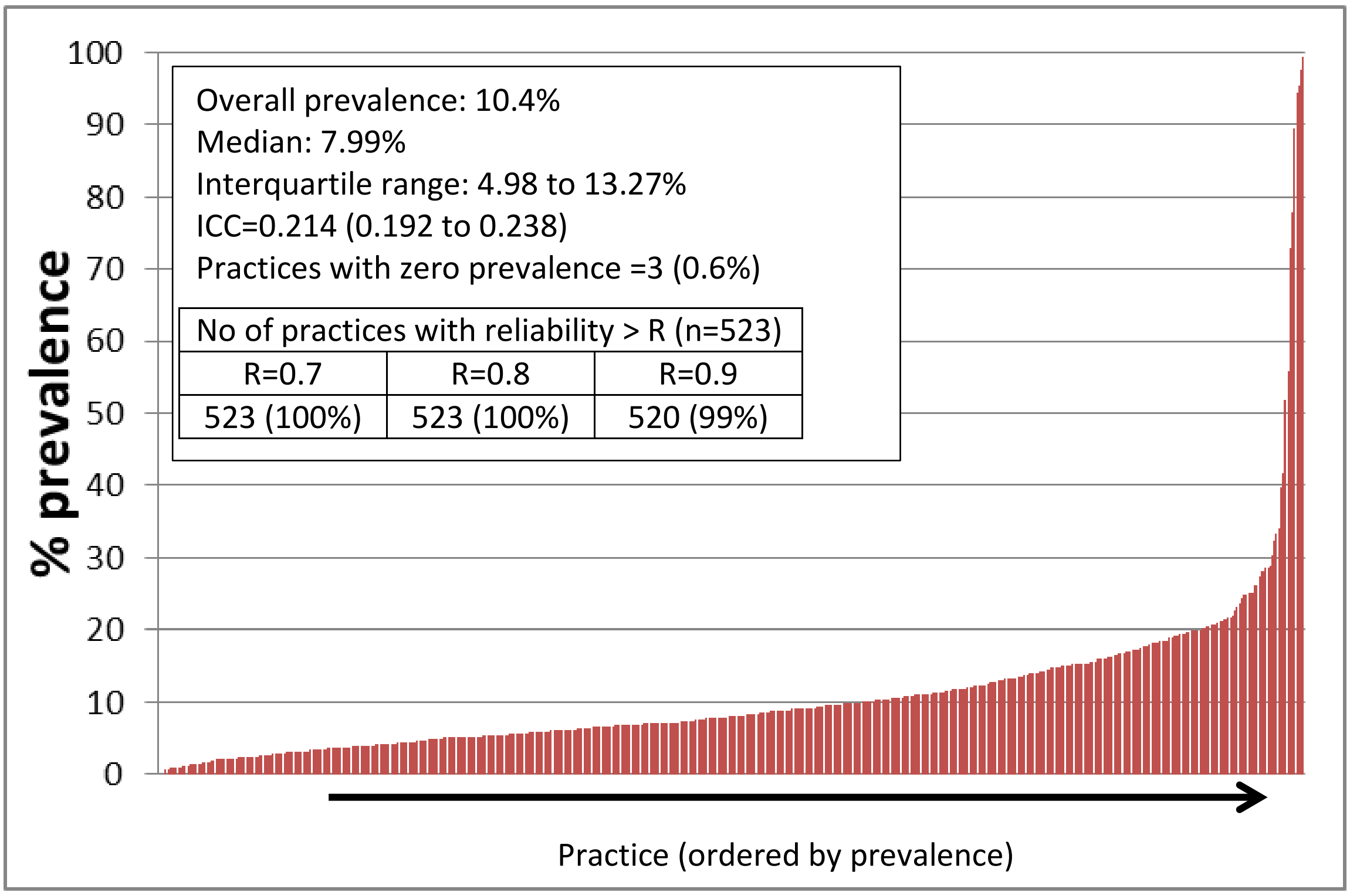

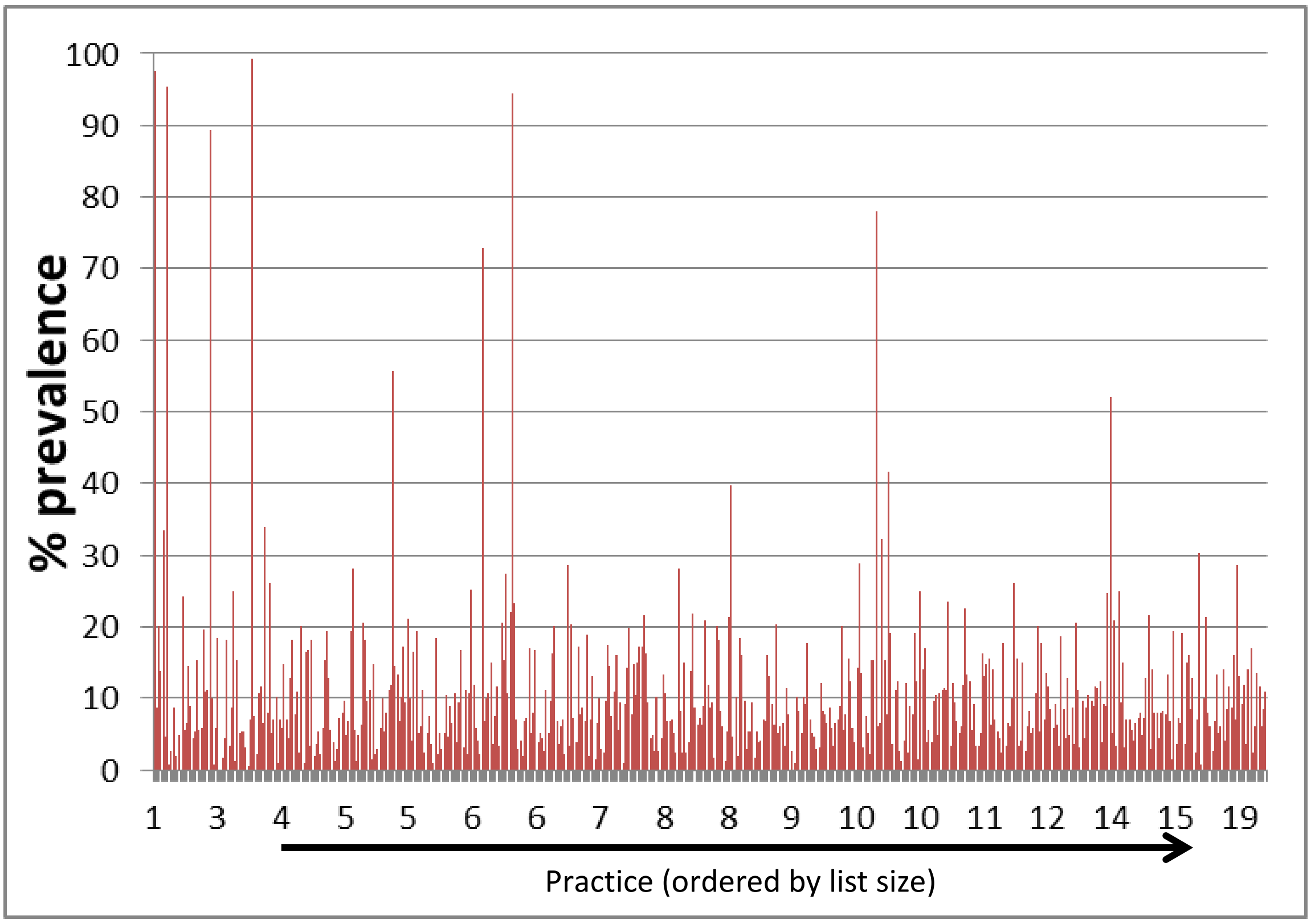

M2. Prescribed repeat amiodarone without thyroid function test

Denominator – group at risk	Numerator – increased risk identified
Has prescription for amiodarone between 6 and 12 months before the audit date and again within the 6 months leading up to the audit date	Have NOT had a thyroid function test in the 6 months leading up to the audit date

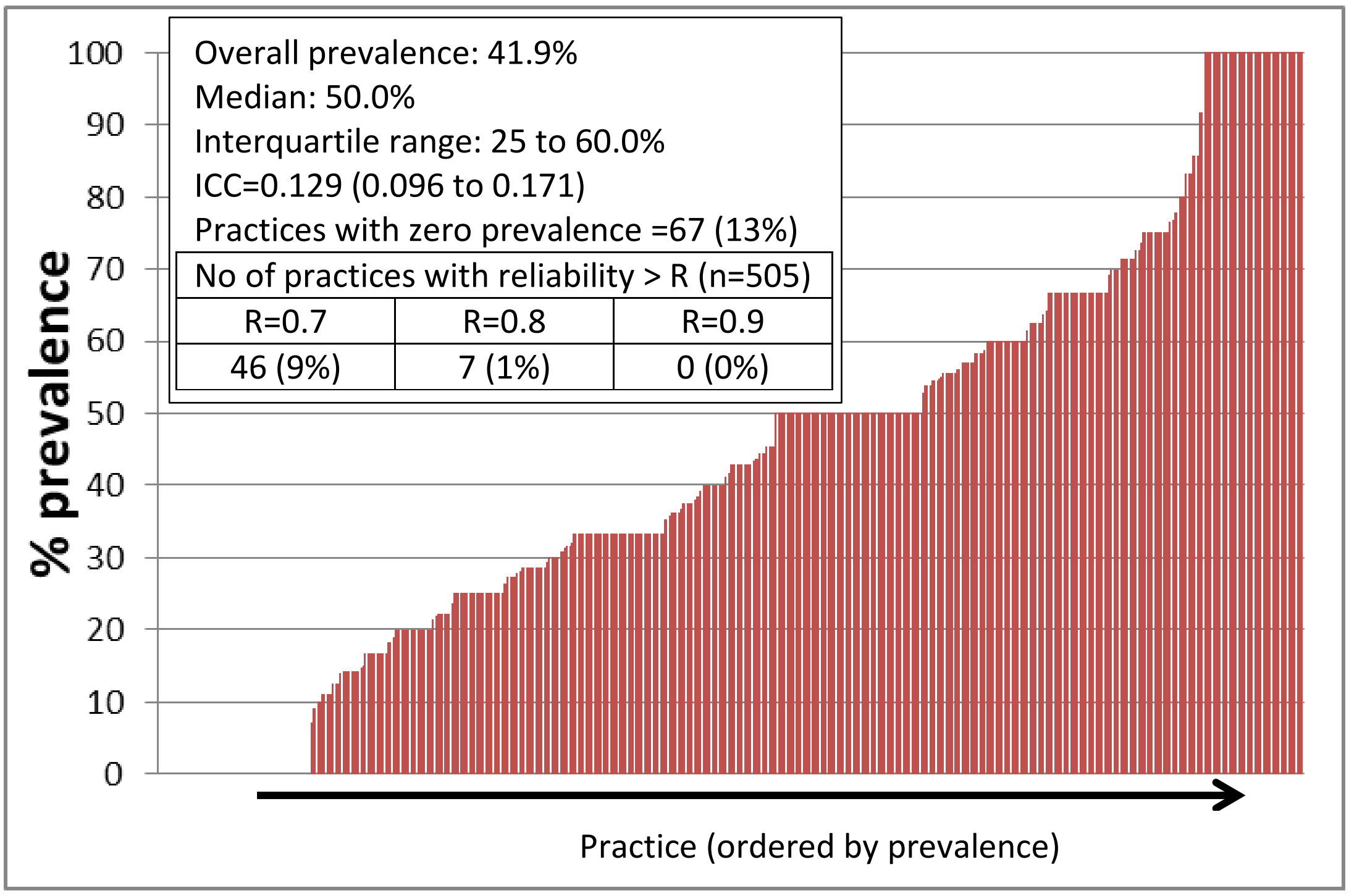

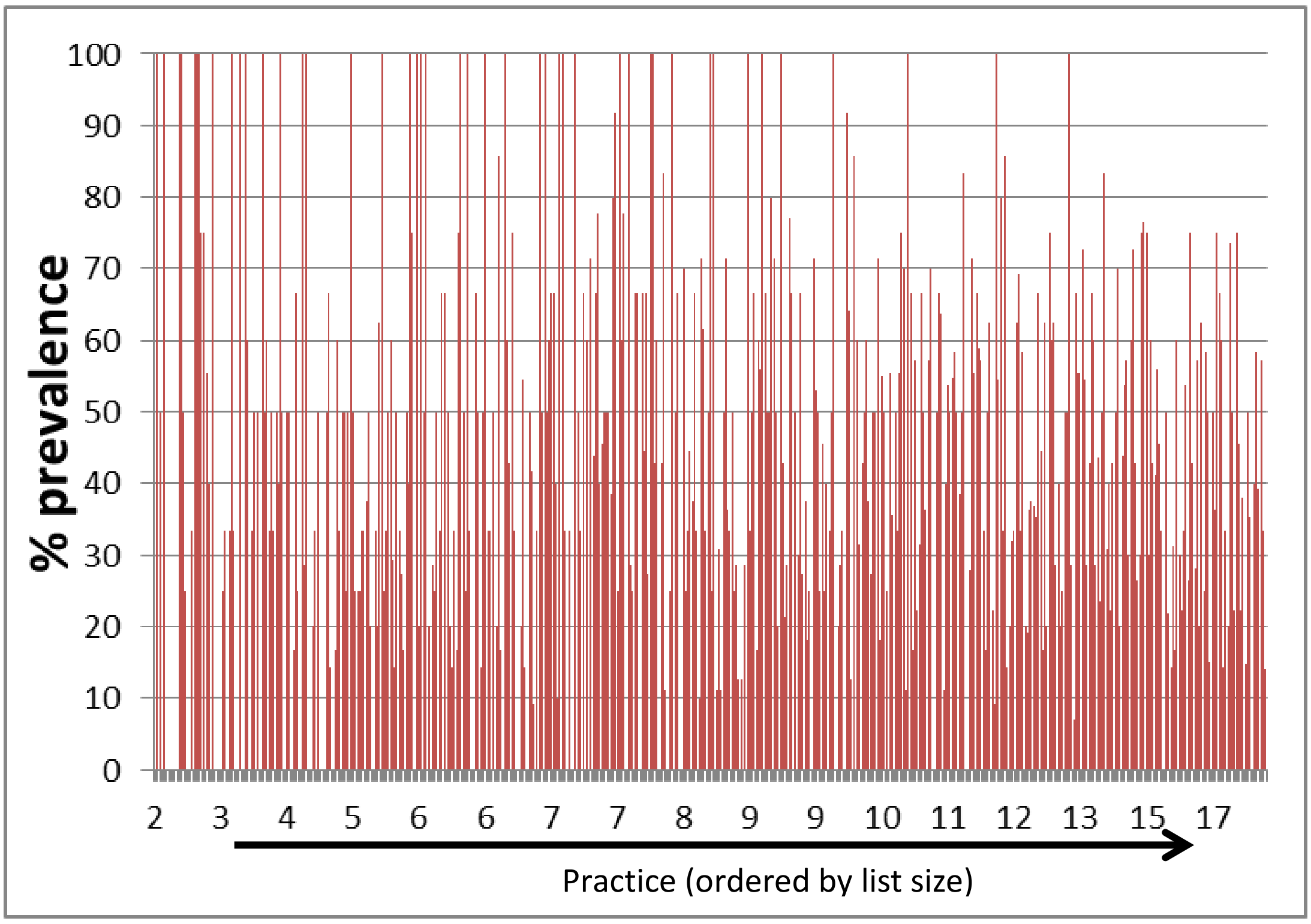

M3. Prescribed repeat methotrexate without liver function test or full blood count

Denominator – group at risk	Numerator – increased risk identified
Has prescription for methotrexate between 3 and 6 months before the audit date and again within the 3 months leading up to the audit date	Have NOT had a liver function test or full blood count in the 3 months leading up to the audit date

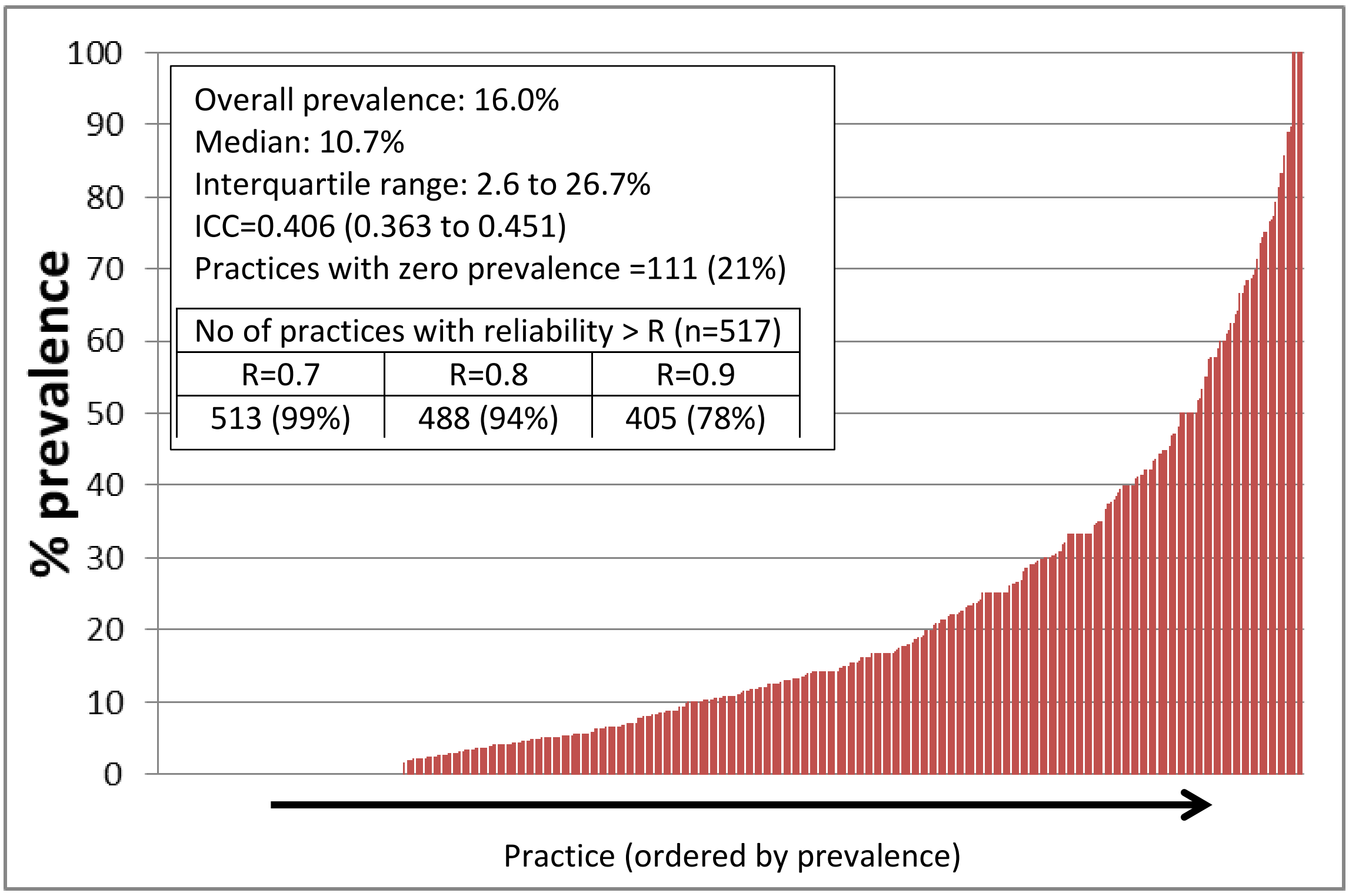

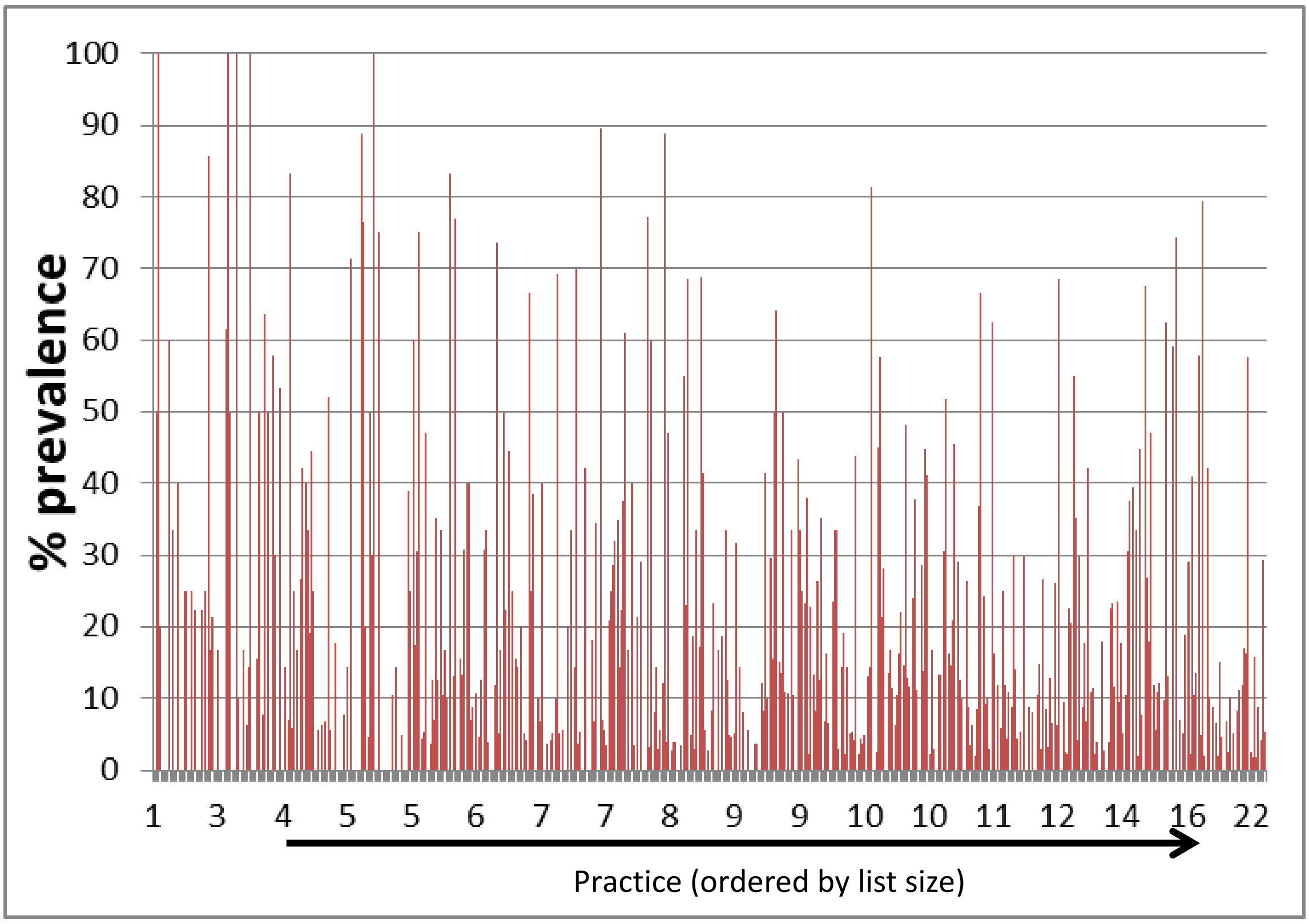

M4. Prescribed repeat lithium without a lithium level test

Denominator – group at risk	Numerator – increased risk identified
Has prescription for lithium between 3 and 6 months before the audit date and again within the 3 months leading up to the audit date	Have NOT had a lithium level test in the 3 months leading up to the audit date

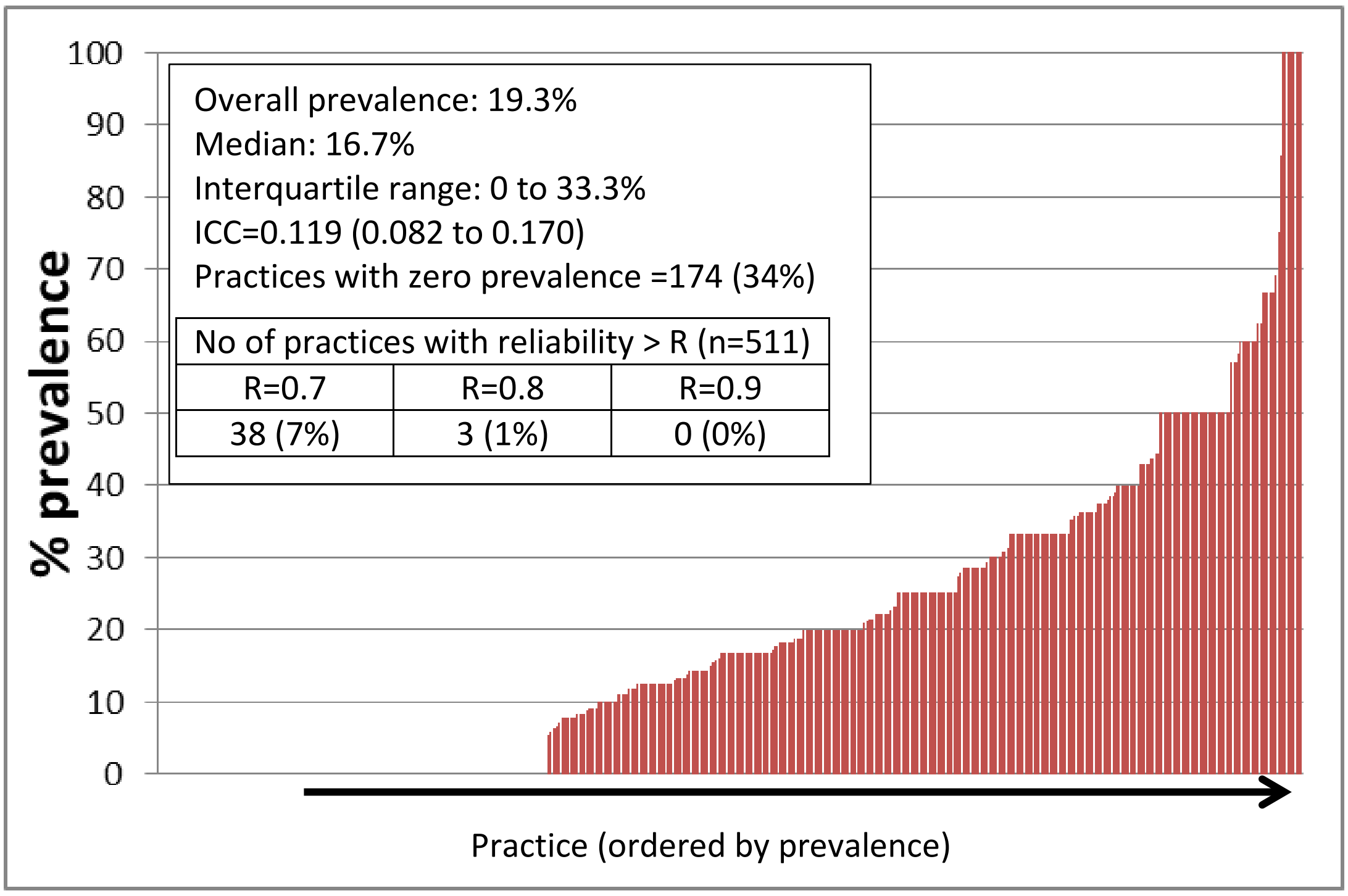

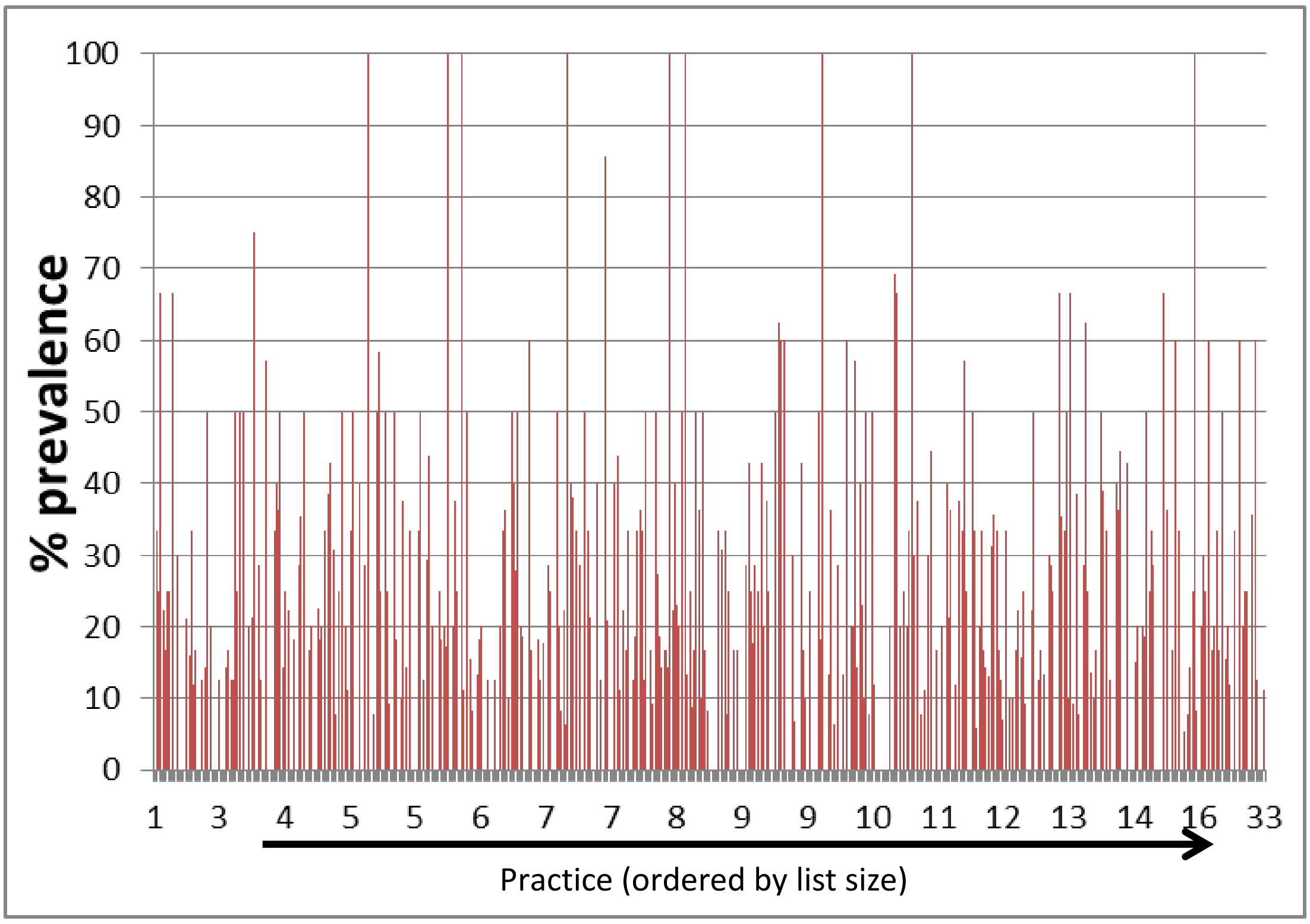

M5. Prescribed repeat warfarin without international normalised ratio (INR) recorded

Denominator – group at risk	Numerator – increased risk identified
Has prescription for warfarin between 3 and 6 months before the audit date and again within the 3 months leading up to the audit date	Have NOT had a check of their international normalized ratio in the 3 months leading up to the audit date

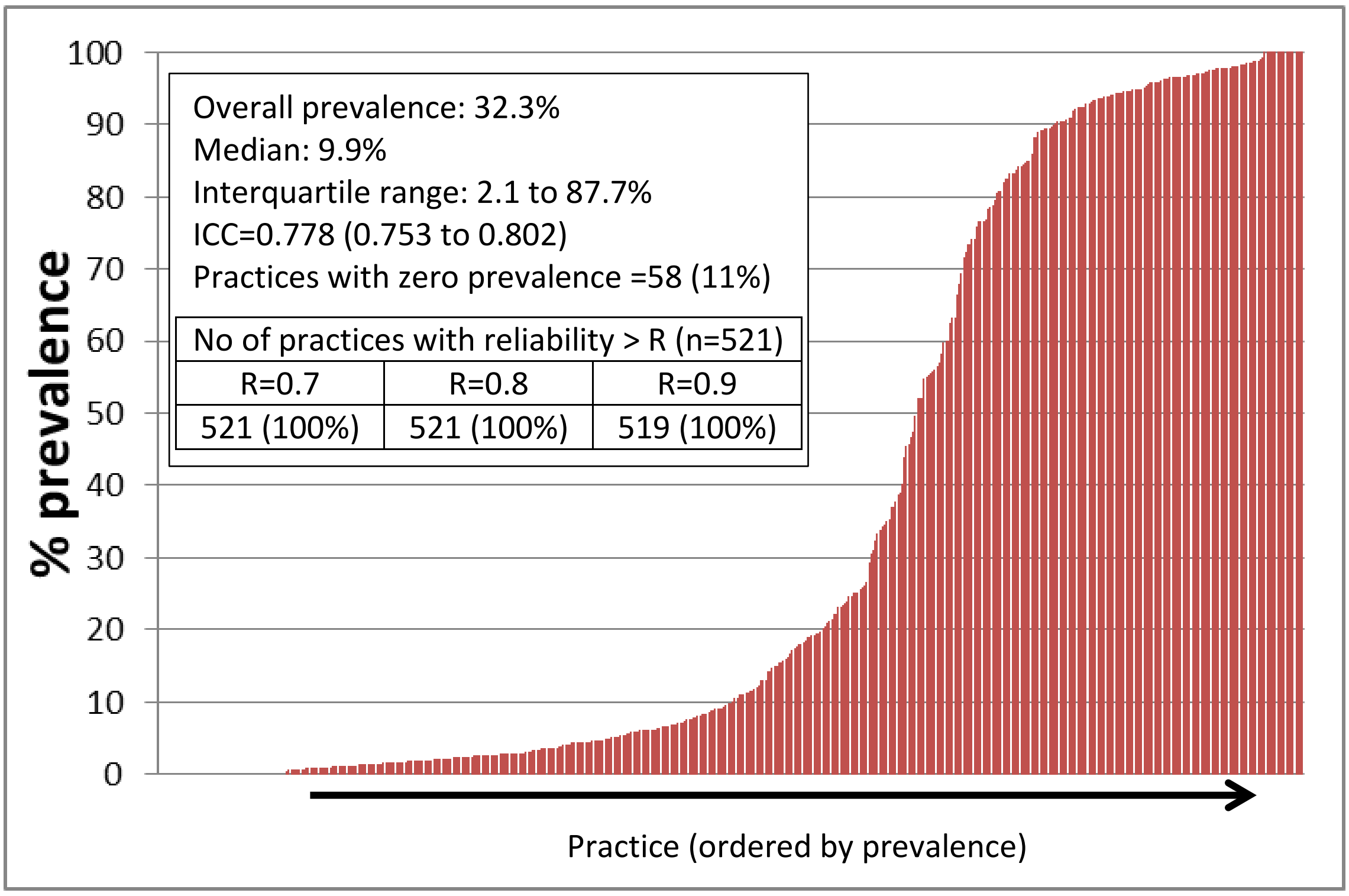

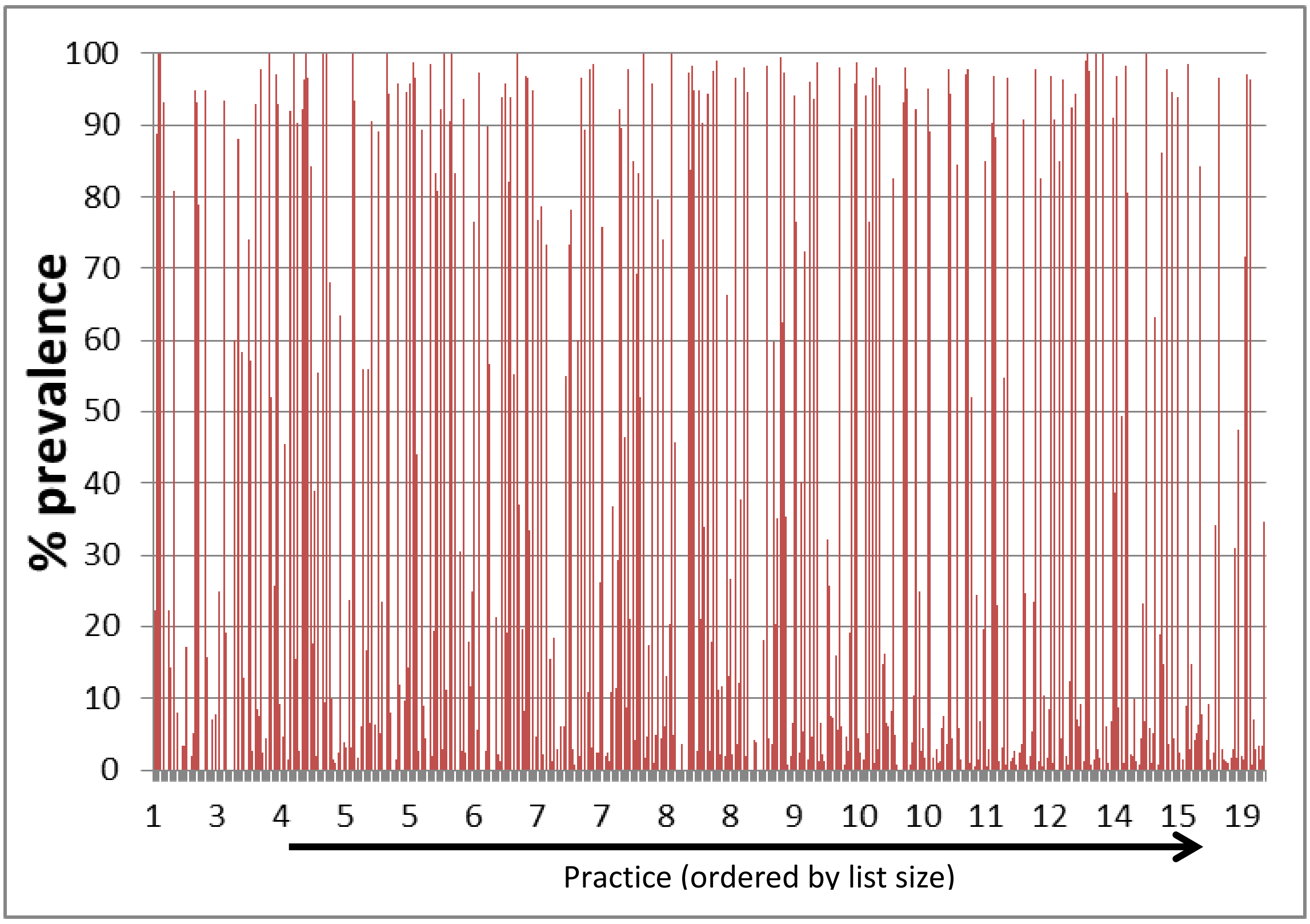

Supplement: Supplementary file 1 [file stos026001.ww1_default.pdf]
