# Supplementary material for: Examining variations in prescribing safety in UK general practice: cross sectional study using the Clinical Practice Research Datalink
Source: BMJ. 2015 Nov 3;351:h5501. doi: 10.1136/bmj.h5501 (PMC4632209; doi:10.1136/bmj.h5501)
Supplement: Supplementary file 2 [file stos026001.ww2_default.pdf]

Appendix 2: Supplementary tables [posted as supplied by author]

Table A. Prevalence of patients receiving at least one high risk prescribing indicator and multilevel adjusted and unadjusted odds ratios – P1-P13 & P16-P19

Variable (number at risk)	% Prevalence (95% CI)	Unadjusted		Adjusted	
		OR	95% CIs	OR	95% CIs
Age					
18-50 (313147)	1.74 (1.69 to 1.79)	1	-	1	-
51-60 (121039)	2.27 (2.19 to 2.36)	1.31	1.25 to 1.37	0.92	0.88 to 0.97
61-70 (264614)	6.29 (6.19 to 6.38)	3.90	3.78 to 4.03	2.54	2.46 to 2.62
71-80 (271421)	6.61 (6.52 to 6.71)	4.14	4.01 to 4.27	2.26	2.19 to 2.33
>80 (225187)	4.39 (4.31 to 4.48)	2.79	2.70 to 2.89	1.41	1.36 to 1.46
No of medications on repeat prescription in last 12 months					
0-1 (457547)	1.25 (1.22 to 1.29)	1	-	1	-
2-4 (291207)	4.19 (4.12 to 4.26)	3.38	3.28 to 3.49	3.08	2.98 to 3.18
5-7 (191501)	6.14 (6.04 to 6.25)	5.09	4.93 to 5.26	4.35	4.21 to 4.49
8-10 (115370)	7.97 (7.81 to 8.13)	6.76	6.53 to 6.99	5.82	5.63 to 6.03
>10 (139783)	9.86 (9.71 to 10.02)	8.58	8.31 to 8.85	7.57	7.32 to 7.82
Gender (18 missing)					
Male (431280)	5.46 (5.39 to 5.53)	1	-	1	-
Female (764110)	3.81 (3.77 to 3.85)	0.68	0.67 to 0.70	0.75	0.73 to 0.76
List size (quartiles)					
<5700 (126844)	4.60 (4.48 to 4.72)	1	-	1	-
5701 to 8600 (233740)	4.78 (4.69 to 4.86)	1.04	0.94 to 1.14	1.02	0.94 to 1.11
8601 to 12100 (336853)	4.45 (4.38 to 4.52)	0.97	0.89 to 1.07	0.98	0.90 to 1.07
>12100 (497971)	4.15 (4.10 to 4.21)	0.91	0.83 to 1.00	0.97	0.89 to 1.06
Practice level index of multiple deprivation					
1-least (228090)	4.37 (4.29 to 4.46)	1	-	1	-
2 (238786)	4.26 (4.18 to 4.34)	1.01	0.91 to 1.12	0.99	0.90 to 1.09
3 (249135)	4.59 (4.51 to 4.67)	1.09	0.98 to 1.21	1.05	0.96 to 1.16
4 (251110)	4.42 (4.34 to 4.50)	1.06	0.95 to 1.18	1.01	0.92 to 1.11
5-most (228287)	4.38 (4.30 to 4.47)	1.03	0.93 to 1.15	0.97	0.88 to 1.07
Region					
North West (144202)	4.82 (4.71 to 4.93)	1	-	1	-
North East (18566)	4.09 (3.81 to 4.38)	0.76	0.58 to 1.00	0.77	0.56 to 1.00
Yorkshire/Humber (13952)	6.45 (6.05 to 6,87)	1.33	0.97 to 1.83	1.35	1.01 to 1.82
East Midlands (7769)	4.08 (3.66 to 4.54)	0.80	0.53 to 1.20	0.78	0.54 to 1.17
West Midlands (111065)	4.48 (4.36 to 4.60)	0.92	0.81 to 1.06	0.93	0.82 to 1.05
East of England (84750)	4.19 (4.06 to 4.33)	0.84	0.72 to 0.97	0.90	0.78 to 1.04
South West (118313)	4.23 (4.12 to 4.35)	0.83	0.72 to 0.95	0.85	0.74 to 0.96
South Central (145906)	4.38 (4.27 to 4.48)	0.88	0.77 to 1.00	0.95	0.84 to 1.07
London (127150)	3.69 (3.59 to 3.79)	0.73	0.65 to 0.82	0.81	0.72 to 0.90
South East Coast (134203)	4.08 (3.98 to 4.19)	0.82	0.72 to 0.93	0.87	0.77 to 0.98
Northern Ireland (38529)	5.79 (5.73 to 6.21)	1.20	1.01 to 1.43	1.10	0.94 to 1.30
Scotland (134245)	4.34 (4.23 to 4.45)	0.88	0.78 to 0.99	0.88	0.79 to 0.99
Wales (116758)	4.73 (4.61 to 4.85)	0.97	0.85 to 1.10	0.90	0.80 to 1.02

Table B. Prevalence of patients receiving at least one high risk prescribing indicator and multilevel adjusted and unadjusted odds ratios – P1-P12

Variable (number at risk)	% Prevalence (95% CI)	Unadjusted		Adjusted	
		OR	95% CIs	OR	95% CIs
Age					
18-50 (197180)	1.54 (1.48 to 1.59)	1	-	1	-
51-60 (54147)	4.87 (4.70 to 5.06)	3.27	3.10 to 3.45	1.64	1.55 to 1.73
61-70 (56337)	8.73 (8.50 to 8.97)	6.09	5.81 to 6.38	2.19	2.08 to 2.30
71-80 (58233)	11.58 (11.32 to 11.84)	8.35	7.99 to 8.73	2.46	2.34 to 2.59
>80 (64108)	9.62 (9.40 to 9.85)	6.89	6.59 to 7.21	2.01	1.91 to 2.11
No of medications on repeat prescription in last 12 months					
0-1 (146654)	0.53 (0.50 to 0.57)	1	-	1	-
2-4 (94718)	2.66 (2.56 to 2.76)	5.17	4.77 to 5.61	4.41	4.06 to 4.79
5-7 (64654)	6.77 (6.58 to 6.97)	13.84	12.81 to 14.95	9.44	8.72 to 10.23
8-10 (48289)	10.59 (10.32 to 10.87)	22.66	21.00 to 24.46	14.30	13.19 to 15.50
>10 (75690)	14.15 (13.90 to 14.40)	31.63	29.39 to 34.05	19.42	17.95 to 21.00
Gender (12 missing)					
Male (194924)	5.76 (5.65 to 5.86)	1	-	1	-
Female (235069)	5.22 (5.14 to 5.32)	0.90	0.88 to 0.92	0.79	0.77 to 0.82
List size (quartiles)					
<5700 (44902)	5.83 (5.62 to 6.05)	1	-	1	-
5701 to 8600 (85905)	5.88 (5.73 to 6.04)	1.00	0.92 to 1.09	1.02	0.95 to 1.10
8601 to 12100 (126904)	5.44 (5.31 to 5.56)	0.91	0.83 to 0.99	0.96	0.89 to 1.04
>12100 (172294)	5.18 (5.08 to 5.29)	0.88	0.81 to 0.96	0.96	0.89 to 1.03
Practice level index of multiple deprivation					
1-least (85889)	5.41 (5.26 to 5.57)	1	-	1	-
2 (84237)	5.20 (5.05 to 5.35)	1.03	0.94 to 1.14	0.96	0.89 to 1.04
3 (86679)	5.54 (5.39 to 5.69)	1.11	1.01 to 1.22	1.00	0.92 to 1.08
4 (86451)	5.60 (5.45 to 5.75)	1.13	1.03 to 1.24	0.98	0.91 to 1.07
5-most (86749)	5.57(5.42 to 5.72)	1.11	1.01 to 1.22	0.98	0.91 to 1.07
Region					
North West (55687)	6.32 (6.12 to 6.52)	1	-	1	-
North East (7044)	5.45 (4.95 to 6.01)	0.83	0.66 to 1.04	0.84	0.68 to 1.03
Yorkshire/Humber (5534)	6.83 (6.20 to 7.53)	1.06	0.82 to 1.37	1.12	0.89 to 1.41
East Midlands (3016)	4.61 (3.92 to 5.42)	0.69	0.49 to 0.98	0.74	0.54 to 1.01
West Midlands (43428)	5.37 (5.16 to 5.59)	0.86	0.77 to 0.96	0.88	0.79 to 0.97
East of England (31059)	5.06 (4.82 to 5.31)	0.78	0.68 to 0.89	0.89	0.79 to 1.01
South West (41448)	5.17 (4.96 to 5.39)	0.81	0.72 to 0.91	0.81	0.73 to 0.90
South Central (56110)	5.24 (5.06 to 5.43)	0.80	0.72 to 0.90	0.94	0.85 to 1.04
London (45009)	4.46 (4.27 to 4.66)	0.71	0.63 to 0.79	0.84	0.76 to 0.93
South East Coast (42596)	4.87 (4.67 to 5.08)	0.76	0.68 to 0.84	0.83	0.75 to 0.92
Northern Ireland (14654)	8.63 (8.18 to 9.09)	1.30	1.12 to 1.51	1.15	1.01 to 1.32
Scotland (42734)	5.75 (5.54 to 5.98)	0.91	0.82 to 1.01	0.86	0.78 to 0.94
Wales (41686)	5.49 (5.28 to 5.71)	0.86	0.77 to 0.97	0.76	0.69 to 0.84

Table C. Prevalence of patients aged over 65 and prescribed NSAID without gastroprotection; multilevel unadjusted and adjusted odds ratios – P13

Variable (number at risk)	% Prevalence (95% CI)	Unadjusted		Adjusted	
		OR	95% CIs	OR	95% CIs
Age					
65-70 (190875)	6.21 (6.10 to 6.32)	1	-	1	-
71-80 (235233)	4.91 (4.83 to 5.00)	0.78	0.76 to 0.80	0.67	0.65 to 0.68
>80 (185495)	2.14 (2.08 to 2.21)	0.34	0.33 to 0.36	0.28	0.27 to 0.29
No of medications on repeat prescription in last 12 months					
0-1 (203688)	1.84 (1.78 to 1.90)	1	-	1	-
2-4 (156870)	5.64 (5.53 to 5.75)	2.99	2.87 to 3.11	3.04	2.93 to 3.17
5-7 (121527)	6.03 (5.90 to 6.17)	3.21	3.08 to 3.34	3.67	3.52 to 3.83
8-10 (68479)	6.14 (5.96 to 6.32)	3.26	3.12 to 3.42	4.04	3.85 to 4.23
>80 (61039)	5.34 (5.16 to 5.52)	2.80	2.66 to 2.94	3.68	3.50 to 3.86
Gender (6 missing)					
Male (278468)	4.61 (4.53 to 4.69)	1	-	1	-
Female (333129)	4.37 (4.30 to 4.44)	0.95	0.92 to 0.97	1.02	0.99 to 1.05
List size (quartiles)					
<5700 (64311)	4.61 (4.45 to 4.78)	1	-	1	-
5701 to 8600 (116655)	4.92 (4.79 to 5.04)	1.09	0.94 to 1.26	1.06	0.93 to 1.21
8601 to 12100 (165308)	4.59 (4.49 to 4.69)	1.03	0.89 to 1.19	1.03	0.90 to 1.18
>12100 (265329)	4.18 (4.11 to 4.26)	0.95	0.83 to 1.10	1.03	0.90 to 1.18
Practice level index of multiple deprivation					
1-least (125316)	4.05 (3.94 to 4.16)	1	-	1	-
2 (125002)	4.33 (4.21 to 4.44)	1.03	0.87 to 1.21	1.03	0.89 to 1.20
3 (132178)	4.73 (4.61 to 4.84)	1.14	0.97 to 1.34	1.14	0.98 to 1.32
4 (127058)	4.63 (4.51 to 4.74)	1.15	0.98 to 1.36	1.11	0.96 to 1.29
5-most (102049)	4.68 (4.55 to 4.81)	1.12	0.95 to 1.32	1.04	0.89 to 1.21
Region					
North West (68645)	4.75 (4.60 to 4.92)	1	-	1	-
North East (8993)	3.89 (3.51 to 4.31)	0.65	0.42 to 1.02	0.68	0.45 to 1.03
Yorkshire/Humber (6699)	7.81 (7.19 to 8.47)	1.71	1.04 to 2.82	1.67	1.04 to 2.67
East Midlands (3654)	4.65 (4.02 to 5.38)	0.97	0.51 to 1.84	0.96	0.52 to 1.76
West Midlands (55535)	4.42 (4.25 to 4.59)	0.90	0.72 to 1.11	0.92	0.75 to 1.12
East of England (44950)	4.16 (3.98 to 4.35)	0.87	0.68 to 1.10	0.93	0.74 to 1.17
South West (64516)	4.25 (4.09 to 4.41)	0.82	0.66 to 1.01	0.86	0.70 to 1.05
South Central (74905)	4.39 (4.24 to 4.54)	0.87	0.71 to 1.07	0.94	0.77 to 1.14
London (65035)	3.72 (3.58 to 3.87)	0.72	0.59 to 0.87	0.78	0.65 to 0.94
South East Coast (75809)	4.20 (4.06 to 4.34)	0.83	0.68 to 1.02	0.90	0.74 to 1.09
Northern Ireland (17249)	5.51 (5.18 to 5.86)	1.10	0.83 to 1.46	1.06	0.81 to 1.38
Scotland (65926)	4.73 (4.57 to 4.90)	0.98	0.81 to 1.18	1.02	0.85 to 1.22
Wales (59687)	5.11 (4.94 to 5.29)	1.07	0.87 to 1.32	1.05	0.86 to 1.27

Table D. Prevalence of patients receiving repeat prescription of ACEI or loop diuretics without urea and electrolyte monitoring; multilevel unadjusted and adjusted odds ratios – M1

Variable (number at risk)	% Prevalence (95% CI)	Unadjusted		Adjusted	
		OR	95% CIs	OR	95% CIs
Age					
75-80 (64002)	9.30 (9.08 to 9.53)	1	-	1	-
>80 (100628)	11.07 (10.88 to 11.27)	1.24	1.19 to 1.28	1.29	1.25 to 1.34
No of medications on repeat prescription in last 12 months					
0-1 (1069)	25.16 (22.65 to 27.85)	1	-	1	-
2-4 (20577)	17.94 (17.42 to 18.47)	0.62	0.54 to 0.73	0.63	0.54 to 0.73
5-7 (41195)	12.32 (12.00 to 12.64)	0.39	0.33 to 0.45	0.39	0.33 to 0.45
8-10 (39441)	9.49 (9.20 to 9.78)	0.28	0.24 to 0.33	0.28	0.24 to 0.32
>10 (62348)	6.92 (6.73 to 7.12)	0.19	0.17 to 0.23	0.19	0.16 to 0.22
Gender					
Male (68131)	8.84 (8.63 to 9.05)	1	-	1	-
Female (96499)	11.47 (11.27 to 11.68)	1.36	1.31 to 1.41	1.35	1.31 to 1.40
List size					
<5700 (18358)	12.05 (11.59 to 12.53)	1	-	1	
5701 to 8600 (33249)	10.74 (10.41 to 11.07)	0.97	0.76 to 1.23	0.96	0.75 to 1.23
8601 to 12100 (47444)	10.06 (9.80 to 10.34)	0.91	0.71 to 1.16	0.88	0.69 to 1.13
>12100 (65579)	9.96 (9.74 to 10.20)	0.95	0.74 to 1.21	0.99	0.77 to 1.26
Practice level index of multiple deprivation (quintiles)					
1 least deprived (31506)	9.56 (9.24 to 9.89)	1	-	1	-
2 (35228)	10.68 (10.36 to 11.00)	1.15	0.87 to 1.51	1.19	0.90 to 1.57
3 (35788)	11.05 (10.73 to 11.37)	1.29	0.98 to 1.70	1.46	1.11 to 1.92
4 (34148)	10.84 (10.52 to 11.18)	1.20	0.91 to 1.58	1.32	1.00 to 1.74
5 most deprived (27960)	9.53 (9.19 to 9.88)	1.09	0.82 to 1.43	1.31	0.99 to 1.75
Region					
North West (18639)	9.47 (9.06 to 9.90)	1	-	1	-
North East (2523)	4.24 (3.52 to 5.10)	0.36	0.17 to 0.78	0.36	0.16 to 0.78
Yorkshire/Humber (1791)	9.66 (8.38 to 11.12)	1.08	0.46 to 2.57	1.06	0.44 to 2.55
East Midlands (1069)	13.75 (11.82 to 15.95)	0.90	0.30 to 2.72	0.92	0.30 to 2.83
West Midlands (16275)	8.22 (7.80 to 8.65)	0.90	0.63 to 1.30	0.89	0.61 to 1.28
East of England (11494)	8.92 (8.41 to 9.45)	1.00	0.66 to 1.50	0.97	0.64 to 1.48
South West (17074)	7.71 (7.32 to 8.12)	0.75	0.52 to 1.09	0.68	0.47 to 0.99
South Central (20326)	13.15(12.69 to 13.62)	1.41	1.00 to 2.00	1.39	0.97 to 2.00
London (16662)	11.72 (11.24 to 12.21)	1.33	0.97 to 1.83	1.27	0.92 to 1.76
South East Coast (20144)	10.14 (9.73 to 10.56)	1.08	0.77 to 1.52	1.00	0.70 to 1.41
N Ireland (5377)	9.32 (8.57 to 10.12)	1.04	0.65 to 1.67	1.16	0.72 to 1.89
Scotland (16950)	14.14 (13.63 to 14.67)	1.14	0.83 to 1.59	1.16	0.83 to 1.62
Wales (16306)	10.17 (9.71 to 10.64)	0.95	0.66 to 1.36	0.95	0.66 to 1.37

Table E. Prevalence of patients receiving repeat prescription of amiodarone, methotrexate or lithium with appropriate monitoring; multilevel unadjusted and adjusted odds ratios – M2-M4

Variable (number at risk)	% Prevalence (95% CI)	Unadjusted		Adjusted	
		OR	95% CIs	OR	95% CIs
Age					
18-50 (3979)	22.12 (20.85 to 23.43)	1	-	1	
51-60 (4070)	19.88 (18.68 to 21.13)	0.83	0.74 to 0.93	0.82	0.73 to 0.93
61-70 (5317)	19.00 (17.96 to 20.07)	0.79	0.70 to 0.88	0.76	0.68 to 0.85
71-80 (4743)	20.81 (19.68 to 21.99)	0.85	0.76 to 0.95	0.82	0.73 to 0.92
>80 (3013)	27.31 (25.75 to 28.93)	1.31	1.16 to 1.47	1.25	1.11 to 1.42
No of medications on repeat prescription in last 12 months					
0-1 (324)	27.47 (22.89 to 32.57)	1	-	1	-
2-4 (3636)	20.49 (19.21 to 21.83)	0.68	0.51 to 0.89	0.72	0.55 to 0.96
5-7 (4894)	20.76 (19.65 to 21.92)	0.69	0.52 to 0.91	0.76	0.57 to 1.00
8-10 (4474)	20.38 (19.23 to 21.59)	0.67	0.51 to 0.89	0.74	0.56 to 0.97
>80 (7794)	22.41 (21.50 to 23.35)	0.76	0.58 to 1.00	0.80	0.61 to 1.06
Gender					
Male (8450)	25.86 (24.94 to 26.80)	1	-	1	-
Female (12672)	18.34 (17.68 to 19.02)	0.62	0.58 to 0.66	0.62	0.57 to 0.66
List size (quartiles)					
<5700 (2368)	21.58 (19.97 to 23.28)	1	-	1	-
5701 to 8600 (4110)	22.24 (20.99 to 23.54)	1.13	0.87 to 1.46	1.07	0.86 to 1.34
8601 to 12100 (6088)	22.73 (21.70 to 23.80)	1.12	0.87 to 1.44	1.03	0.82 to 1.28
>12100 (8556)	19.87 (19.04 to 20.73)	1.00	0.78 to 1.29	0.94	0.75 to 1.17
Practice level index of multiple deprivation					
1-least deprived (4036)	19.52 (18.33 to 20.78)	1	-	1	-
2 (4489)	20.78 (19.62 to 22.00)	1.19	0.90 to 1.57	1.02	0.80 to 1.30
3 (4560)	20.79 (19.64 to 21.99)	1.10	0.84 to 1.45	1.14	0.89 to 1.44
4 (4447)	21.92 (20.73 to 23.16)	1.35	1.03 to 1.78	1.33	1.05 to 1.69
5-most deprived (3590)	24.09 (22.72 to 25.52)	1.36	1.03 to 1.80	1.37	1.07 to 1.75
Region					
North West (2261)	19.11 (17.54 to 20.78)	1	-	1	-
North East (263)	21.29 (16.78 to 26.64)	0.92	0.47 to 1.77	0.90	0.46 to 1.75
Yorkshire/Humber (214)	34.58 (28.53 to 41.17)	2.56	1.26 to 5.21	2.32	1.13 to 4.77
East Midlands (153)	13.73 (9.16 to 20.07)	0.75	0.29 to 1.93	0.84	0.33 to 2.19
West Midlands (1830)	32.40 (30.30 to 34.58)	2.24	1.64 to 3.07	2.30	1.68 to 3.15
East of England (1483)	24.28 (22.16 to 26.52)	1.41	1.00 to 2.00	1.56	1.10 to 2.23
South West (1882)	13.23 (11.77 to 14.84)	0.67	0.48 to 0.93	0.68	0.48 to 0.94
South Central (2753)	19.07 (17.65 to 20.58)	1.08	0.80 to 1.46	1.18	0.86 to 1.61
London (2002)	36.66 (34.58 to 38.80)	2.66	2.01 to 3.53	2.78	2.09 to 3.69
South East Coast (2541)	25.54 (23.88 to 27.27)	1.60	1.19 to 2.14	1.68	1.24 to 2.27
Northern Ireland (1023)	13.88 (11.90 to 16.13)	0.67	0.44 to 1.02	0.68	0.45 to 1.04
Scotland (2672)	15.38 (14.06 to 16.80)	0.79	0.59 to 1.06	0.82	0.61 to 1.10
Wales (2045)	12.86 (11.48 to 14.38)	0.56	0.40 to 0.78	0.56	0.41 to 0.79
